# Frontal sinuses and human evolution

**DOI:** 10.1126/sciadv.abp9767

**Published:** 2022-10-21

**Authors:** Antoine Balzeau, Lou Albessard-Ball, Anna Maria Kubicka, Andréa Filippo, Amélie Beaudet, Elena Santos, Thibault Bienvenu, Juan-Luis Arsuaga, Antonis Bartsiokas, Lee Berger, José María Bermúdez de Castro, Michel Brunet, Kristian J. Carlson, Joan Daura, Vassilis G. Gorgoulis, Frederick E. Grine, Katerina Harvati, John Hawks, Andy Herries, Jean-Jacques Hublin, Jiaming Hui, Rachel Ives, Josephine A. Joordens, Yousuke Kaifu, Mirsini Kouloukoussa, Baptiste Léger, David Lordkipanidze, Ann Margvelashvili, Jesse Martin, María Martinón-Torres, Hila May, Aurélien Mounier, Anton du Plessis, Todd Rae, Carolin Röding, Montserrat Sanz, Patrick Semal, Dominic Stratford, Chris Stringer, Mirriam Tawane, Heiko Temming, Evangelia Tsoukala, João Zilhão, Bernhard Zipfel, Laura T. Buck

**Affiliations:** ^1^ UMR 7194 Histoire Naturelle de l’Homme Préhistorique, CNRS, PaleoFED Team, Département Homme et Environnement, Muséum national d’Histoire naturelle, Paris, France.; ^2^ Department of African Zoology, Royal Museum for Central Africa, Tervuren, Belgium.; ^3^ PalaeoHub, Department of Archaeology, University of York, York, UK.; ^4^ Department of Zoology, Poznań University of Life Sciences, Poznań, Poland.; ^5^ Department of Archaeology, University of Cambridge, Cambridge, UK.; ^6^ School of Geography, Archaeology and Environmental Studies, University of the Witwatersrand, Johannesburg, South Africa.; ^7^ Institut Català de Paleontologia Miquel Crusafont, Universitat Autònoma de Barcelona, Barcelona, Spain.; ^8^ Centro Mixto UCM-ISCIII de Evolución y Comportamiento Humanos, Departamento de Paleontología, Facultad Ciencias Geológicas, Universidad Complutense de Madrid, 28040 Madrid, Spain.; ^9^ Cátedra de Otoacústica Evolutiva y Paleoantropología (HM Hospitales - Universidad de Alcalá), Departamento de Ciencias de la Vida, Universidad de Alcalá, Alcalá de Henares, Spain.; ^10^ Centro Nacional de Investigación sobre la Evolución Humana (CENIEH), Paseo de la Sierra de Atapuerca 3, 09002 Burgos, Spain.; ^11^ Department of Anthropology and Anthropological Museum, University of Zurich, CH-8052 Zurich, Switzerland.; ^12^ Department of History and Ethnology, Democritus University of Thrace, Komotini, Greece.; ^13^ Centre for the Exploration of the Deep Human Journey, University of the Witwatersrand, WITS, Johannesburg 2050, South Africa.; ^14^ Anthropology Department, University College London, London, UK.; ^15^ Chaire de Paléoanthropologie Humaine, Collège de France, Paris, France.; ^16^ UMR 7262 CNRS, Université de Poitiers, Poitiers, France.; ^17^ Evolutionary Studies Institute, University of the Witwatersrand, Palaeosciences Centre, Wits, Johannesburg 2050, South Africa.; ^18^ Department of Integrative Anatomical Sciences, Keck School of Medicine, University of Southern California, Los Angeles, CA 90089, USA.; ^19^ Departament d’Història i Arqueologia, Facultat de Geografia i Història, Universitat de Barcelona, c/Montalegre 6, 08001 Barcelona, Spain.; ^20^ Centro de Arqueologia da Universidade de Lisboa (UNIARQ), Faculdade de Letras de Lisboa, Universidade de Lisboa, Alameda da Universidade, 1600-214 Lisboa, Portugal.; ^21^ Department of Histology and Embryology, Medical School, National and Kapodistrian University of Athens, Athens, Greece.; ^22^ Biomedical Research Foundation of the Academy of Athens, Athens, Greece.; ^23^ Ninewells Hospital and Medical School, University of Dundee, Dundee, UK.; ^24^ Division of Cancer Sciences, School of Medical Sciences, Faculty of Biology, Medicine and Health, University of Manchester, Manchester M20 4GJ, UK.; ^25^ Departments of Anthropology and Anatomical Sciences, Stony Brook University, Stony Brook, NY 11794, USA.; ^26^ Senckenberg Center for Human Evolution and Paleoenvironment and Institute for Archaeological Sciences, Eberhard Karls Universität Tübingen, Rümelinstr. 23, 72070 Tübingen, Germany.; ^27^ University of Wisconsin-Madison, Madison, WI 53706, USA.; ^28^ Department of Archaeology and History, La Trobe University, Bundoora, VIC 3086, Australia.; ^29^ Palaeo-Research Institute, University of Johannesburg, Gauteng, South Africa.; ^30^ Department of Human Evolution, Max Planck Institute for Evolutionary Anthropology, D-04103 Leipzig, Germany.; ^31^ Chaire de Paléoanthropologie, Collège de France, 75005 Paris, France.; ^32^ Centre for Human Evolution Research, History Museum, London, UK.; ^33^ Naturalis Biodiversity Center, Leiden, Netherlands.; ^34^ Faculty of Science and Engineering, Maastricht University, Netherlands.; ^35^ The University Museum, The University of Tokyo, Hongo 7-3-1, Bunkyo-ku, Tokyo 113-0033, Japan.; ^36^ Museum of Anthropology, Medical School, National and Kapodistrian University of Athens, Athens, Greece.; ^37^ Columbia University, 116 Street & Broadway, New York, NY 10027, USA.; ^38^ Georgian National Museum, Purtseladze Str. 3, 0105 Tbilisi, Georgia.; ^39^ Ivane Javakhishvili Tbilisi State University, Chavchavadze Av. 1, 0179 Tbilisi, Georgia.; ^40^ Palaeoscience, Department of Archaeology and History, La Trobe University, Bundoora, VIC 3086, Australia.; ^41^ Department of Anatomy and Anthropology, Sackler Faculty of Medicine, Tel Aviv University, Post Office Box 39040, Tel Aviv 6997801, Israel.; ^42^ Shmunis Family Anthropology Institute, Dan David Center for Human Evolution and Biohistory Research, Sackler Faculty of Medicine, Tel Aviv University, Tel Aviv 6997801, Israel.; ^43^ Physics Department, Stellenbosch University, Stellenbosch, South Africa.; ^44^ Centre for Research in Evolutionary Anthropology, Department of Life Sciences, Roehampton University, Holybourne Avenue, London SW15 4JD, UK.; ^45^ Paleoanthropology, Senckenberg Centre for Human Evolution and Palaeoenvironment, Eberhard Karls University of Tübingen, Tübingen, Germany.; ^46^ Grup de Recerca del Quaternari (GRQ-SERP), Departament d’Història i Arqueologia, Universitat de Barcelona, Carrer Montalegre, 6, 08001 Barcelona, Spain.; ^47^ Royal Belgian Institute of Natural Sciences, Brussels 1000, Belgium.; ^48^ School of Geography, Archaeology and Environmental Studies, University of the Witwatersrand, WITS, Johannesburg 2050, South Africa.; ^49^ Ditsong National Museum of Natural History, Pretoria, South Africa.; ^50^ Laboratory of Geology and Palaeontology, School of Geology, Aristotle University of Thessaloniki, 54124 Thessaloniki, Greece.; ^51^ UNIARQ-Centro de Arqueologia da Universidade de Lisboa, Faculdade de Letras, Universidade de Lisboa, 1600-214 Lisbon, Portugal.; ^52^ Catalan Institution for Research and Advanced Studies, 08010 Barcelona, Spain.; ^53^ Department of History and Archaeology, University of Barcelona, 08007 Barcelona, Spain.; ^54^ Evolutionary Studies Institute, University of the Witwatersrand, Johannesburg, South Africa.; ^55^ Research Centre for Evolutionary Anthropology and Palaeoecology, School of Biological and Environmental Sciences, Liverpool John Moores University, Liverpool, UK.

## Abstract

The frontal sinuses are cavities inside the frontal bone located at the junction between the face and the cranial vault and close to the brain. Despite a long history of study, understanding of their origin and variation through evolution is limited. This work compares most hominin species’ holotypes and other key individuals with extant hominids. It provides a unique and valuable perspective of the variation in sinuses position, shape, and dimensions based on a simple and reproducible methodology. We also observed a covariation between the size and shape of the sinuses and the underlying frontal lobes in hominin species from at least the appearance of *Homo erectus*. Our results additionally undermine hypotheses stating that hominin frontal sinuses were directly affected by biomechanical constraints resulting from either chewing or adaptation to climate. Last, we demonstrate their substantial potential for discussions of the evolutionary relationships between hominin species.

## Teaser

Variation in frontal sinus shape and dimensions has high potential for phylogenetic discussion when studying human evolution.

## INTRODUCTION

Sinus presence and morphology have been used in systematics in phylogenetically disparate taxa (*1*, *2*), and there is some evidence that pneumatic variation may be diagnostic in Middle and Late Pleistocene hominins (*3*–*6*). Relationships between hominins remain far from clear and potentially phylogenetically informative characters such as frontal sinus morphology that may be useful in elucidating them. Among extant primates, ethmoidally derived frontal sinuses are present only in *Gorilla*, *Pan*, and *Homo* (*7*, *8*), and we have recently characterized morphology in these genera (*9*). Knowledge of the variation in size and shape of frontal pneumatization during human evolution, however, is limited. In original specimens, pneumatization is usually only directly observable when they are fragmented. In this context, most of the evidence resides in brief descriptions that appear occasionally as part of detailed presentations of fossil individuals. The few comparative studies of hominin frontal sinuses to date have been based on restricted samples and focused either on comparing *Homo neanderthalensis* to *Homo sapiens* or on the distinctive sinuses of Middle Pleistocene *Homo* (*3*, *6*, *10*, *11*). Moreover, frontal sinus shapes show great complexity and extreme levels of variation within and between taxa. Imaging and quantification of such variation are difficult, and most studies have therefore focused on quantitative analysis of size. Here, we apply a simple, repeatable method for quantifying and comparing sinus shape and position and additionally quantify and compare frontal sinus size in a large sample of extinct hominins and extant nonhuman African apes.

In addition to uncertainty regarding the taxonomic patterning of frontal sinus variation, debate continues over their potential function. Many varied explanations have been proposed for sinus function (*12*–*14*), from aquatic adaptation (*15*) to acoustic adaptations (*16*). Two hypotheses of enduring popularity are that sinuses are a thermoregulatory adaptation [e.g., (*17*)] or that they serve to disperse masticatory strain [e.g., (*18*)]. An alternative is that sinuses have no function at all and are evolutionary spandrels in the sense described by Gould and Lewontin (*19*). The different hominin sinus types (frontal, maxillary, sphenoidal, and ethmoidal) are not functionally or developmentally homologous (*6*, *20*); thus, we focus here solely on the frontal sinuses. Although we do not explicitly test functional hypotheses, our results from comparisons of frontal sinus morphology among hominin and nonhuman primate taxa are informative in the context of this debate.

The present study aims to (i) quantify variation in frontal sinus shape and size for each available hominin species, using linear and volumetric measurements, and to compare these results to large samples of extant hominines; and (ii) to describe and quantify patterns of bilateral variation in the frontal sinuses to investigate the possible relationship between sinus form and the position of the underlying frontal lobes of the brain (as reflected by the endocast) following patterns suggested by our previous investigation of extant taxa (*9*).

Our results bring original and previously unknown insights to understanding the origin of hominin frontal pneumatization by highlighting the phylogenetic importance of this character and its relationship with other aspects of the cranium. Our study also contributes strongly to the characterization of hominin cranial anatomy in different taxa.

## RESULTS

### Sinus size and shape variation

Descriptive information illustrating variation in frontal sinus morphology between and within a total of 21 hominin species (69 fossil hominin specimens) is given in the Supplementary Materials (Supplementary Text and figs. S2 to S70 in frontal, lateral, and superior orientations) and summarized in Fig. 1. We also detail information on the available imaging dataset and comment on preservation of the relevant anatomical area for each fossil hominin individual (table S2). Morphometric data for pneumatization of the frontal bone for each individual are presented in table S3, and an illustration of the measurements taken is shown in Fig. 2. This detailed information is provided for future comparative purposes.

**Fig. 1.**
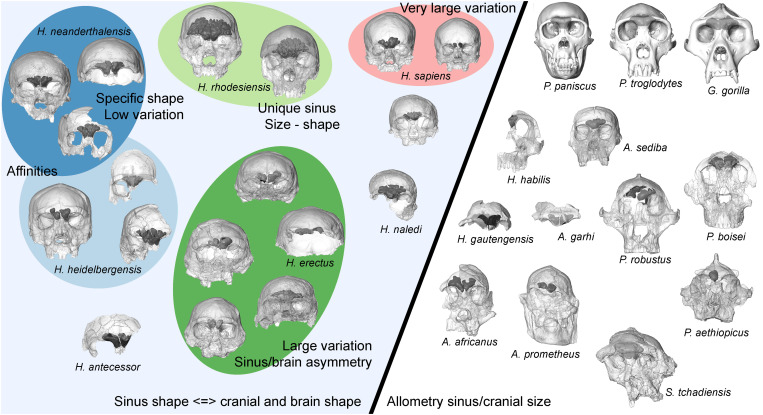
Schematic diagram summarizing variation among taxa and evolutionary changes in hominin frontal sinus morphology. The diagonal line divides taxa that seem to show different constraints on sinus morphology (specimens are not shown to scale; they are globally organized chronologically from base to top). Weak constraint on sinus development from surrounding anatomical structures and large frontal superstructures providing potential space for expansion give the sinuses the opportunity to develop isometrically with endocranial size (Fig. 3) in genera *Pan*, *Gorilla*, *Sahelanthropus*, *Australopithecus*, and *Paranthropus* (see right). In later hominins (see left), integration between the cranium, brain, and sinuses appears to influence sinuses expansion. Within later *Homo*, characteristics of sinus morphology are indicated by different colored ellipses (color code corresponds to that used inFigs. 3 to 5). Our results support the existence of separate groups within Middle Pleistocene hominins. On the basis of the frontal sinuses, there appears to be an evolutionary relationship between *H. neanderthalensis* and one group, which may be called *H. heidelbergensis*, while the group containing Broken Hill 1, and so reasonably called *H. rhodesiensis*, has a unique morphology that supports a distinct status. Covariation between the size and shape of the sinuses on both sides and the underlying frontal lobes has existed since at least *H. erectus* and was present among subsequent hominin species.

**Fig. 2.**
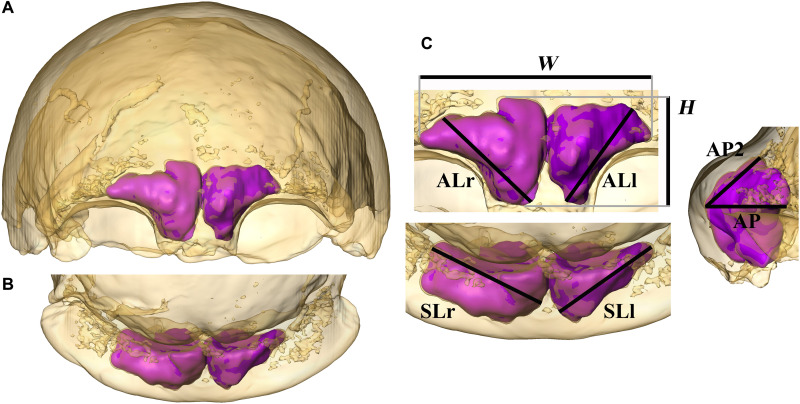
Visualization and quantification of the frontal sinuses. The skull of the type specimen of *H. neanderthalensis*, Feldhofer 1, in anterior (**A**), superior (**B**), and detailed views (**C**). Bone is rendered transparent, and sinuses are shown as a virtual solid in magenta. Dimensions of the frontal sinuses are measured as 2D projections in different orientations and are shown as follows: maximal lateral extension (*W*), maximal height (*H*), and maximal length of the left and right frontal sinuses [anterior length (AL): ALl and ALr] measured from the most medial and inferior point of the sinus to the more distant point of the extension of the sinus vertically and laterally measured in anterior view; maximal medio-lateral extension of the left and right sinus [superior length (SL): SLl and SLr] measured in superior view; length from the most anteriorly protruding point of the sinus to the most posterior point in a horizontal direction (AP) and length from the most anterior point to the maximal supero-posterior extension of the sinuses (AP2) measured in left lateral view (see also fig. S1).

**Fig. 3.**
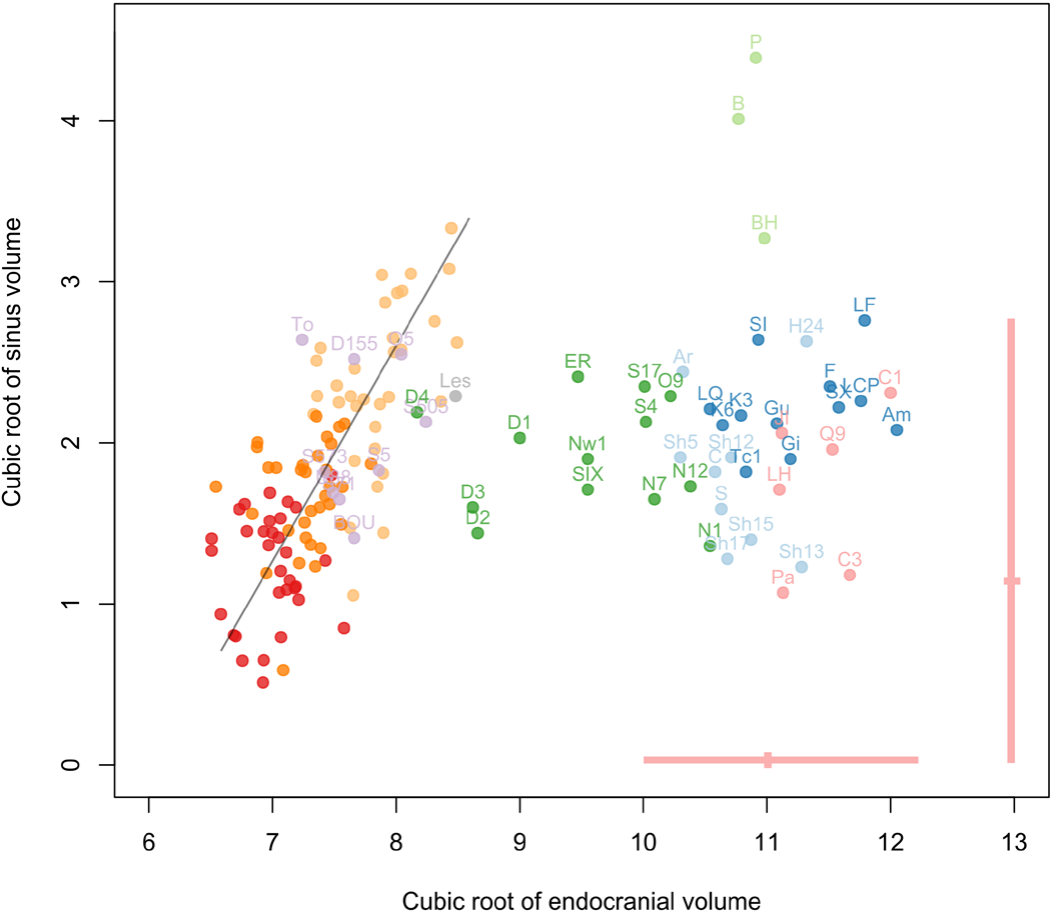
Bivariate plot of the cube root of the volume of the frontal sinuses relative to the cube root of the endocranial volume. Individual volume data (both in centimeters) in *Pan* and *Gorilla* (red: *P. paniscus*; dark orange: *P. troglodytes*; light orange: *G. gorilla*) and fossils hominins (purple: *Sahelanthropus*, *Australopithecus*, and *Paranthropus*; gray: *H. naledi*; green: *H. erectus s.l.*; light blue: *H. heidelbergensis*; light green: *H. rhodesiensis*; dark blue: *H. neanderthalensis*; pink: fossil *H. sapiens*); the pink lines show the variation and the mean for recent *H. sapiens*. The black line shows the regression of both variables in *Pan* and *Gorilla* (slope = 1.36, *r* = 0.72, *P* = 10 × 10^−16^). Labels for fossil hominins are as follows: TM 266-01-060-01 (Toumaï), To; Sts 5, S5; Stw 505, S505; Sts 71, S71; Stw 573, S573; UW 88-50, U88; BOU-VP-12/130, BOU; SK 48, S48; DNH 155, D155; OH 5, O5; Stw 53, S53; KNM-ER 3883, ER; OH 9, O9; D2280, D1; D2282, D2; D3444, D3; D4500, D4; Trinil 2, T; Sambungmacan 4, S4; Sangiran 17, S17; Skull IX, SIX; Ngandong 1, N1; Ngandong 7, N7; Ngandong 12, N12; Ngawi 1, Nw1; Lesedi 1, Les; La Ferrassie 1, LF; La Quina H5, LQ; Guattari, Gu; Forbes’ Quarry 1, Gi; Krapina 3, K3; Krapina 6, K6; La Chapelle aux Saints, LCP; Spy 1, SI; Spy 10, SX; Feldhofer, F; Amud, Am; Tabun C1, Tc1; Aroeira, Ar; HK 87, H87; H1024, H24; Sima de los Huesos Skull 5, Sh5; SHS12, Sh12; SHS13, Sh13; SHS17, Sh17; Ceprano, C; Petralona, P; Broken Hill 1, BH; Bodo, B; Zuttiyeh, Z; Steinheim, S; Jebel Irhoud 1, JI; LH 18, LH; Qafzeh 9, Q9; Cro-Magnon 1, C1; Cro-Magnon 2, C2; Cro-Magnon 3, C3; Pataud, Pa.

The relationship between the cube root of the volume of the frontal sinuses and the cube root of the endocranial volume is informative regarding global variation in frontal sinus size (Fig. 3). There was a significant correlation between sinus size and endocranial size when the complete sample for *Pan* and *Gorilla* is considered [slope = 1.36, correlation coefficient (*r*) = 0.72, *P* = 10 × 10^−16^]. We observed a nonsignificant relationship and large variation in sinus size independently of endocranial size (slope = 2.17, *r* = 0.089, *P* = 0.53) when fossil hominins were included with nonhuman great apes. The variation in hominin sinus volume fell within the range of variation observed in *Pan* and *Gorilla*, with a few exceptions (Fig. 3). Broken Hill 1 is at the upper extreme of variation, such that only one *Gorilla gorilla* specimen has larger sinuses, while Bodo and Petralona have the largest sinuses in the entire sample. Fossil hominins with small brain sizes plot within the endocranial variation observed for *Pan* and *Gorilla*, which tend to show a linear relationship between brain volume and sinus size. This is the case for *Sahelanthropus tchadensis* (TM 266-01-060-1), *Australopithecus africanus* (Sts 5 and 71, StW 505), *Australopithecus prometheus* (StW 573), *Australopithecus garhi* (Bou-VP-12/130), *Australopithecus sediba* (U.W. 88), *Paranthropus robustus* (DNH 155), and *Paranthropus boisei* (OH 5). Two *Homo* individuals also plot within this variation: the *Homo naledi* individual Lesedi 1 and D4500 from Dmanisi. Early *Homo* fossils would probably also plot in this distribution if we could measure both their endocranial size and the volume of their frontal sinuses. Other hominin species, with larger brains, do not follow this pattern and plot outside of this distribution. *H. sapiens* (that includes large samples of recent individuals and several fossil individuals with all the characteristic features of our species) has a particular position in relation with restricted pneumatization compared to other species, in terms of both the absolute extension and the proportion of unpneumatized individuals.

**Fig. 4.**
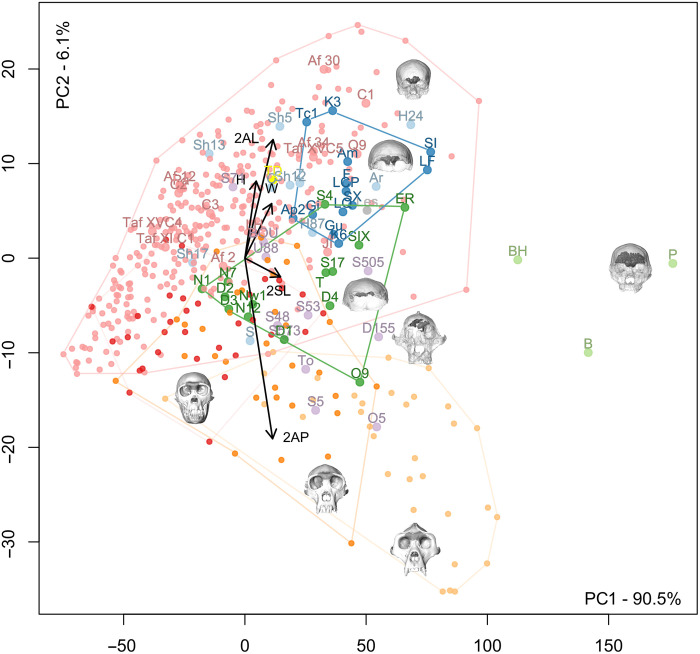
PCA of absolute measurements of frontal pneumatization in all directions. Red: *P. paniscus*; dark orange: *P. troglodytes*; light orange: *G. gorilla*; small pink dots: extant *H. sapiens*; purple: *Sahelanthropus*, *Australopithecus*, and *Paranthropus*; gray: *H. naledi*; dark green: *H. erectus s.l.*; light blue: *H. heidelbergensis*; light green: *H. rhodesiensis*; dark blue: *H. neanderthalensis*; large pink dots: fossil *H. sapiens*. *H*, height of sinuses; *W*, width of sinuses; 2AL, combined medio-lateral extension of sinuses in anterior view; 2AP, combined anterior projection of sinuses; 2SL, combined medio-lateral extension of sinuses in superior view (the lines represent the direction and the loadings of these measurements along the two components; see Fig. 1 for the visualization of these measurements). Labels for fossil hominins are as follows: TM 266-01-060-01 (Toumaï), To; Sts 5, S5; Stw 505, S505; Sts 71, S71; Stw 573, S573; UW 88-50, U88; BOU-VP-12/130, BOU; SK 48, S48; DNH 155, D155; OH 5, O5; Stw 53, S53; KNM-ER 3883, ER; OH 9, O9; D2280, D1; D2282, D2; D3444, D3; D4500, D4; Trinil 2, T; Sambungmacan 4, S4; Sangiran 17, S17; Skull IX, SIX; Ngandong 1, N1; Ngandong 7, N7; Ngandong 12, N12; Ngawi 1, Nw1; Lesedi 1, Les; La Ferrassie 1, LF; La Quina H5, LQ; Guattari, Gu; Forbes’ Quarry 1, Gi; Krapina 3, K3; Krapina 6, K6; La Chapelle aux Saints, LCP; Spy 1, SI; Spy 10, SX; Feldhofer, F; Amud, Am; Apidima 2, Ap2; Tabun C1, Tc1; TD6-15, TD (yellow dot); Aroeira, Ar; HK 87, H87; H1024, H24; Sima de los Huesos Skull 5, Sh5; SHS12, Sh12; SHS13, Sh13; SHS17, Sh17; Ceprano, C; Petralona, P; Broken Hill 1, BH; Bodo, B; Zuttiyeh, Z; Steinheim, S; Jebel Irhoud 1, JI; LH 18, LH; Qafzeh 9, Q9; Cro-Magnon 1, C1; Cro-Magnon 2, C2; Cro-Magnon 3, C3; Pataud, Pa.

**Fig. 5.**
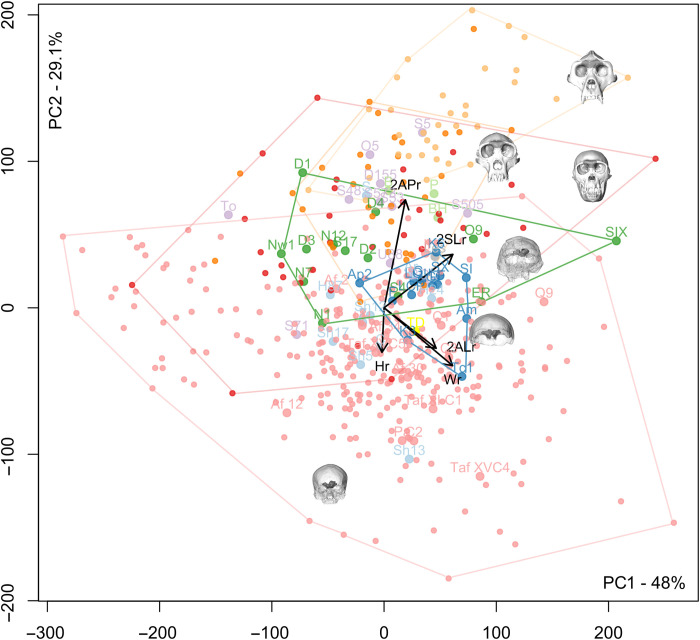
PCA of relative measurements of frontal pneumatization in all directions. Red: *P. paniscus*; dark orange: *P. troglodytes*; light orange: *G. gorilla*; small pink dots: extant *H. sapiens*; purple: *Sahelanthropus*, *Australopithecus*, and *Paranthropus*; gray: *H. naledi*; dark green: *H. erectus s.l.*; light blue: *H. heidelbergensis*; light green: *H. rhodesiensis*; dark blue: *H. neanderthalensis*; large pink dots: *H. sapiens*. Hr, relative height of sinuses; Wr, relative width of sinuses; 2ALr, relative combined medio-lateral extension of sinuses in anterior view; 2APr, relative combined anterior projection of sinuses; 2SLr, relative combined medio-lateral extension of sinuses in superior view (the lines represent the direction and the loadings of these measurements along the two components; see Fig. 1 for the visualization of these measurements; the cube root of the volume of the pneumatization was used for trait size correction). Labels for fossil hominins are as follows: TM 266-01-060-01 (Toumaï), To; Sts 5, S5; Stw 505, S505; Sts 71, S71; Stw 573, S573; UW 88-50, U88; BOU-VP-12/130, BOU; SK 48, S48; DNH 155, D155; OH 5, O5; Stw 53, S53; KNM-ER 3883, ER; OH 9, O9; D2280, D1; D2282, D2; D3444, D3; D4500, D4; Trinil 2, T; Sambungmacan 4, S4; Sangiran 17, S17; Skull IX, SIX; Ngandong 1, N1; Ngandong 7, N7; Ngandong 12, N12; Ngawi 1, Nw1; Lesedi 1, Les; La Ferrassie 1, LF; La Quina H5, LQ; Guattari, Gu; Forbes’ Quarry 1, Gi; Krapina 3, K3; Krapina 6, K6; La Chapelle aux Saints, LCP; Spy 1, SI; Spy 10, SX; Feldhofer, F; Amud, Am; Apidima 2, Ap2; Tabun C1, Tc1; TD6-15, TD (yellow dot); Aroeira, Ar; HK 87, H87; H1024, H24; Sima de los Huesos Skull 5, Sh5; SHS12, Sh12; SHS13, Sh13; SHS17, Sh17; Ceprano, C; Petralona, P; Broken Hill 1, BH; Bodo, B; Zuttiyeh, Z; Steinheim, S; Jebel Irhoud 1, JI; LH 18, LH; Qafzeh 9, Q9; Cro-Magnon 1, C1; Cro-Magnon 2, C2; Cro-Magnon 3, C3; Pataud, Pa.

A principal components analysis (PCA) of all the linear measurements computed on the data before adjustment for size illustrates the strong influence of sinus size on distinguishing extant and fossil groups. Separation on the first axis is mainly due not only to size (Fig. 4) but also to shape, particularly in the antero-posterior and lateral dimensions, which separate out *Gorilla* from the other taxa, and in the supero-inferior dimensions, which separate out recent *H. sapiens*. *Pan* and fossil hominins have an intermediate position on the first axis. Broken Hill 1, Petralona, and Bodo have large values on the first axis and are isolated on this axis. A PCA computed on relative (size-adjusted) data distinguishes well on the second axis between recent/fossil *H. sapiens* and *Pan*/*Gorilla*, while, again, fossil hominin species have intermediate positions (Fig. 5).

In the analyses of both absolute and relative measurements, the early hominin individuals, which include *Sahelanthropus*, *Australopithecus*, and *Paranthropus*, plot together and fall between *Pan* and *Gorilla* on the one hand and the later hominins on the other. *H. naledi* plots in the center of the distribution of *Homo erectus* for both the absolute and relative size analyses. *H. erectus s.l.* shares with *H. sapiens* a higher degree of variation in sinus extension. The *H. neanderthalensis* and most European Middle Pleistocene hominin distributions overlap partly in both analyses. A group composed of Broken Hill 1, Bodo, and Petralona plots outside of the distribution of all other hominin samples due to their much larger sinuses (Fig. 4). In terms of relative dimensions, these three fossils plot at the intersection of early hominins and *H. erectus* because of their relatively large antero-posterior sinus dimensions compared to other hominin species (Fig. 5). The individual attributed to *Homo antecessor* plots well within the distribution of other Middle Pleistocene hominins for both absolute and relative analyses. TD6-15 has absolutely smaller sinuses, possibly relating to its individual age, than the *H. neanderthalensis* individuals (table S3, Fig. 4, and fig. S34) but falls within the *H. neanderthalensis* range of variation for the relative data (Fig. 5). Fossil and recent *H. sapiens* have relatively small sinuses of distinctively great height (supero-inferior dimensions). These morphometric results complement the visible variation in sinus shape (Supplementary Text and table S4).

### Morphometric trends between *H. sapiens* populations

When a linear discriminant analysis of the different geographic samples of recent *H. sapiens* was computed on the dataset of absolute measurements, the resulting confusion matrix showed a proportion of correctly classified individuals of 33.9%. This low level of correct classification illustrates the large variation observed within samples and a lack of geographic partitioning in sinus size/shape. A multivariate analysis of variance, however, shows some significant differences between pairs of samples that were investigated through additional analyses. The resulting squared Mahalanobis distances (table S5) highlight some closer affinities and differences between groups, but there is no clear geographic patterning. Last, we tested for a potential correlation between sinus dimensions and geography. To do so, a PCA was calculated on sinus dimensions of the different samples of extant *H. sapiens*. PC1 accounts for 91.95% of the variation in this extant human sample. Spatial autocorrelation was not observed in the dataset (Mantel test, *P* = 0.1662), which means that the frontal sinus measurements in one population are not more similar to those of geographic neighbors than they are to groups at a greater distance. We also calculated a generalized linear model (table S6) to see whether individuals from various regions differed in the measurements of their frontal sinuses. These results illustrate that, although the Mantel tests showed that the dimensions of the frontal sinuses are not spatially autocorrelated, they differed significantly between geographic regions (see *P* values in table S6).

### Bilateral variation in sinus and brain anatomy

Within the recent *H. sapiens* sample, among 345 individuals, 41 do not have sinuses (aplasia), 24 have no sinus on the right side, and 9 have no sinus on the left side. There is a tendency toward greater sinus extension toward the left, resulting in significant directional asymmetry (DA). The mean values right minus left (R-L) for anterior length (AL) and superior length (SL) are −2.23 and −2.25 mm (*t* = −4.3113, *P* = 2.2 × 10^−05^; *t* = −3.806, *P* = 0.0002, respectively). This shows that frontal sinuses in *H. sapiens* tend to be larger on the left side, as mean values (R-L) for AL and SL are negative and significantly different from zero. Asymmetrical sinus development in recent *H. sapiens* has been shown to be associated with right petalias (frontal lobe expansion in the brain) (*9*). On the left side, it appears that sinus development takes advantage of the greater space available, resulting in greater pneumatization. In *Pan* and *Gorilla*, no DA was detected for all the dimensions of the frontal sinuses (*9*), while petalias in these taxa do show some degree of asymmetry (*21*).

To identify potential relationships between sinus and cranial or brain morphology in fossil hominin taxa, we investigated several features at the individual level among our sample, including bilateral variation in sinus dimensions (relative to their preservation), the shape of the supraorbital torus (as directly observed for each individual), and the position of the underlying frontal lobes of the brain as reflected by the endocranial cast (including the petalia, i.e., the relative extension of respectively the right and left frontal poles). It was necessary to consider specimens individually due to poor preservation in many individuals precluding sample-wide analysis. In Sts 5 and StW 505 (*A. africanus*), there appears to be a left frontal petalia of the brain associated with a smaller sinus on the left side. In U.W. 88 (*A. sediba*), a right frontal petalia is associated with a larger left sinus. We observe a tendency among our sample of Asian *H. erectus* for the left sinus to be larger than the right sinus, and most of these fossils show a right frontal petalia. The exception is Sangiran 17, which has a left frontal petalia. In Middle Pleistocene hominins, the left sinus tends to be larger than the right sinus, and a right frontal petalia is observed in all individuals that allow evaluation of this trait. In *H. neanderthalensis*, there is no clear asymmetric tendency in the sinuses at the scale of the sample, which might result from the incomplete preservation in many individuals. Nevertheless, a right frontal petalia is the most common pattern and appears to be paired with qualitatively greater extension of the left sinuses (R petalia and L > R sinus) in La Ferrassie 1, Gibraltar 1, Krapina 3, and Spy 1.

## DISCUSSION

### Frontal bone pneumatization and phylogenetic implications

With regard to the relationship between sinus size and endocranial size, early hominins, including *Sahelanthropus* and various species of *Australopithecus* and *Paranthropus*, plot comfortably within the range of variation observed for *Pan* and *Gorilla* and at some distance from the distribution of *Homo* individuals (Fig. 3). This pattern is maintained in multivariate analyses of sinus dimensions (Figs. 4 and 5). The underlying frontal lobes do not appear to influence sinus shape and expansion in *Pan*, *Gorilla*, and early hominins, while the large frontal superstructures in these taxa give the sinuses the opportunity to develop isometrically relative to brain size, in contrast to the condition observed among *Homo* species. This morphology, shared not only between *Pan* and *Gorilla* (*9*) but also with *Sahelanthropus*, *Australopithecus*, and *Paranthropus*, could be seen as a primitive trait in contrast with the different conditions observed in *Homo* individuals. Nevertheless, with the potential exception of Stw 53, which has been tentatively attributed to *Homo gautengensis* (*22*) but more conventionally to *Australopithecus* sp. (*23*) or *Homo* cf. *habilis* (*24*, *25*) and shows the primitive frontal sinus size and shape, we feel that this finding cannot be used to clarify the taxonomic attribution of disputed early hominin fossils. With regard to Stw 53, its primitive sinus morphology may support its exclusion from the genus *Homo.*

In contrast to the relative homogeneity of sinus morphology in nonhuman apes and early hominins, we observe differences in frontal sinus size and shape between *H. erectus s.l.*, *H. neanderthalensis*, Middle Pleistocene hominins, and fossil *H. sapiens*. Our results suggest that frontal pneumatization develops in *Homo* species in relation to new and variable constraints related to factors such as the integration between the vault and the upper face, brain, and frontal sinuses. These groups of *Homo* share a reduced antero-posterior extension of the sinuses compared to early hominins and show variation in the extension of the sinuses in the lateral and vertical directions, depending on taxon (table S4). These differences may be an indirect consequence of the differences in cranial morphology between taxa, as already suggested [e.g., (*6*, *10*)], and of different evolutionary trajectories. However, that does not prevent their potential utility in taxonomic analyses.

Despite its relatively small brain size, *H. naledi* does not follow the pattern of frontal pneumatization seen in other small-brained hominins but is in the center of the range of variation observed for *H. erectus s.l.* both for multivariate analyses of absolute and relative data. This is a previously unknown and important observation for this species and supports its inclusion in the genus *Homo* notwithstanding many primitive aspects of its morphology (*26*).

Both *H. erectus s.l.* and *H. sapiens* show relatively great variation in the size and shape of the frontal sinuses (table S4), including a sizeable proportion of aplasia. The Zhoukoudian, Ngandong, and Sambungmacan individuals tend to have small sinuses, and several exhibit aplasia (tables S2 and S3). Sinuses are larger in the more ancient Indonesian and African *H. erectus* individuals. The five Dmanisi individuals constitute an interesting illustration of the level of sinus morphology variation that can be observed in *H. erectus s.l*. They show a very high degree of intraspecific sinus morphology variation, although they come from a unique stratigraphic layer from the same site. This is congruent with the elevated craniofacial variability observed in this sample (*27*). High levels of variation in craniodental anatomy in *H. erectus* as a species have been noted [e.g. (*28*, *29*)] and have caused some to argue that the taxon should be divided (*30*, *31*). However, many see this level of variation as commensurate with what should be expected in a long-lived, geographically widespread primate species (*28*, *32*).

High levels of variation in sinus size and shape are visible among Middle Pleistocene hominins, particularly because of the huge pneumatization of Bodo, Broken Hill 1, and Petralona. These individuals are unique in terms of the size and shape of their sinuses, which might support their grouping in a separate taxon that could be called *Homo rhodesiensis* due to the presence of the holotype of the species in the group (i.e., Broken Hill 1). Regarding the debated taxonomic position of Zuttiyeh [(*33*) versus (*34*)], the individuals’ frontal pneumatization shares more affinities with *H. neanderthalensis* than with *H. erectus s.l.* The other European Middle Pleistocene specimens (to the exclusion of Broken Hill 1, Bodo, and Petralona, if grouped into *H. rhodesiensis*) exhibit a coherent morphological pattern of frontal pneumatization, which differs from the other groups. For this reason, these fossils are grouped here into *Homo heidelbergensis* for ease of discussion (but note that not all authors of this paper agree on the use of *H. heidelbergensis* for all specimens within this group). In terms of absolute data, these individuals form a group close to *H. neanderthalensis* except for Sima de los Huesos 13 and 17, in which the sinuses are smaller, and Steinheim, which plots with *H. erectus*. The morphology of the frontal pneumatization of Steinheim may nevertheless reflect taphonomic alteration. When relative dimensions are considered, the *H. heidelbergensis* fossils are slightly further outside the range of *H. neanderthalensis*.

A greater degree of variation is observed in earlier European fossils, but they also share clear affinities in sinus shape with the most recent *H. neanderthalensis*, which exhibit reduced variation in sinus shape and size compared to other fossil populations. The specimens from Sima de los Huesos slightly expand the observed variation in the *H. neanderthalensis* sample if included therein. *H. neanderthalensis* do not have absolutely or relatively larger sinuses compared to other hominins (*6*, *10*, *35*). These observations on frontal sinuses are consistent with a potential phylogenetic relationship between at least some fossils named here *H. heidelbergensis* and *H. neanderthalensis*, as suggested by craniofacial morphological evidence from the Sima de los Huesos, Steinheim, and Ehringsdorf individuals [e.g., (*5*, *30*, *36*, *37*)] and by genetic data from the Sima de los Huesos hominins (*38*). Other members of the group described here as *H. heidelbergensis* may show different taxonomic affinities. Analyses of temporal bone pneumatization have also shown very low levels of morphological variation in *H. neanderthalensis* (*39*). This is interesting in the context of ancient DNA studies, which have demonstrated low levels of genetic diversity and a high frequency of inbreeding within later representatives of *H. neanderthalensis* (*38*, *40*).

### Causes and modalities of expression of the frontal sinuses

Our results illustrate various patterns of sinus variation in *Gorilla*, *Pan*, and hominin species. We see different sinus morphologies between taxa likely undergoing relatively similar masticatory strain regimes, such as *H. erectus s.l.* and Middle Pleistocene hominins (*41*, *42*). On the basis of these observations and previous evidence (*41*, *42*), it is very unlikely that sinus size and shape are driven by masticatory strains in hominins.

Our results, along with multiple strands of evidence in the literature (*6*, *10*, *35*), demonstrate that *H. neanderthalensis* individuals are not hyperpneumatized compared to *H. sapiens*, *H. erectus*, or other hominin samples in terms of absolute or relative frontal pneumatization. In addition, there is no clear support for a functional or a climatic origin of *H. neanderthalensis* pneumatization when all of the evidence (*43*), including observed variation among hominins, is considered. On the basis of this multiple evidence, we, therefore, propose that the long-standing hypothesis that the frontal sinuses of *H. neanderthalensis* are an adaptation to cold climate [e.g., (*17*, *44*, *45*)], should be definitively rejected.

Moreover, our results obtained from large, diverse samples of *H. sapiens* (Supplementary Text and tables S5 and S6) show that the dimensions of the sinuses are not spatially autocorrelated, despite significant differences between geographic regions. In other words, no direct link is observed between geographic origin and the size and shape of frontal sinuses, i.e., individuals from colder climates are not characterized by significantly larger/smaller frontal sinuses than populations from warmer areas. The observed differences within the analyzed sample appear to be related to other factors besides climate. We propose then that climate does not seem to directly explain the development of frontal sinuses in our species. It is nevertheless likely that sinus shape and variation in living populations around the world may reflect some aspects of the recent history of our species including migrations, genetic drift, and local adaptations. Whether these factors exert selective pressures on the sinuses themselves or indirectly via their effects on craniofacial morphology remains to be determined.

### Does frontal lobe shape influence sinus shape?

A more anterior, lateral extension of the right frontal lobe of the brain, as reflected by the endocranial cast, compared to the contralateral side is a general pattern in hominins that becomes consistent in *H. erectus*, *H. heidelbergensis*, *H. neanderthalensis* (*46*, *47*), and *H. sapiens*. This asymmetry is a well-known feature of *H. sapiens*, which has been described in the neuroscientific literature (*48*, *49*, *50*). Our observations of fossil taxa (above and as detailed in the Supplementary Materials) support the suggestion that covariation between the size and shape of the sinuses on both sides and the underlying frontal lobes (*9*) was present in hominin species from at least the appearance of *H. erectus*. Last, a greater extension of the frontal sinuses into the larger space resulting from a contralateral petalia suggests that sinus development is at least, in part, opportunistic (*10*).

### A new perspective on frontal sinuses and human evolution

This research opens original perspectives for the study of frontal sinuses. A limiting factor in this analysis of complex internal anatomical traits during human evolution is the available information for the hominin fossil record. Moreover, the comparison of the different features of bilateral frontal sinus morphology among fossil hominins is complicated. Several fossils do not have fully preserved bilateral pneumatization, and taphonomic alteration may alter the shape and size of the sinuses on each side, complicating the analyses of subtle bilateral differences. The small sample size for fossil hominins also prevents large-scale analyses of directional and fluctuating asymmetry. This is why we have considered here several features at the individual level among our fossil hominin sample, including the bilateral variation of the dimensions of the sinuses in relation to their preservation, the shape of the torus, and the position of the underlying frontal lobes to identify potential relationships between anatomical features and repeated patterns among hominin samples. More generally, fossil preservation and relatively low resolution for imaging datasets are problematic for paleoanthropological research. Data access is another issue. Fortunately, we had here access to a unique database to study the variation and evolution of the hominin anatomy, yet there are still taxa that we were unable to access. The sample for this study is more complete and diverse, in terms of hominin species and fossil individuals included, than any previous study on paranasal pneumatization. The internal preservation of the crania and the capacity of the imaging data to allow visualization of the features studied have to be considered, nevertheless. Our simple and pragmatic protocol allows for a large, precise, and detailed study of this complex fossil record. In this context, we revise previous incomplete or erroneous characterizations of sinus morphology for some fossil individuals or species and obtain original information on the majority of the material (see Supplementary Text).

We propose a simple, repeatable methodology for the anatomical description and quantification of sinus size and shape, as well as a global comparative morphometric and anatomic framework for nearly all the identified hominin species. We hope that this will encourage authors of future descriptions of key hominin skulls to report detailed information about the morphology and dimensions of the sinuses. This does not prevent researchers from doing additional comparative analyses of their individuals but provides the paleoanthropological community with basic knowledge of a potentially important area of anatomy in fossil hominins.

On the basis of the available evidence, we conclude that large frontal superstructures induce weak constraints related to the position of the face and the brain and give the sinuses the opportunity to expand allometrically in all directions into the available space in the genera *Gorilla*, *Pan*, *Sahelanthropus*, *Australopithecus*, and *Paranthropus*. In later hominins, new and variable constraints related to developmental integration between the cranium, brain, and frontal sinuses as well as the timing of growth and development of all these structures result in limitations in the opportunistic expansion of the sinuses into the osseous structures of the frontal bone. This different condition results in a lower antero-posterior extension of the sinuses compared to early hominins and *Pan*/*Gorilla*. However, differences in sinus shape and size are also observed among later *Homo* species, and these may have some implications for phylogenetic discussions and open original perspectives for specific studies to better interpret the origin of these different patterns (e.g., to investigate the role of the face). Future research on extant species should compare the shape and size of the skull, the face, and the base together with the observed variations for frontal sinuses, but application to the fossil record will be, in essence, difficult. Concerning the causes and modalities of the expression of the sinuses, our results are in agreement with the assertion that sinus size and shape are not driven by adaptation to masticatory strains in hominins nor due to climatic adaptation.

## MATERIALS AND METHODS

Our materials consist of imaging datasets, including a large number of fossil hominins (*N* = 94; table S2) that are separated into different geographic and/or chronological groups as follows (species names that have been proposed are also mentioned): early hominins: TM 266-01-060-1 (*S. tchadensis*), Taung, Sts 5, Sts 71, StW 505 (*A. africanus*), StW 573 (*A. prometheus*), BOU-VP-12/130 (*A. garhi*), U.W. 88 (*A. sediba*), KNM-WT 17000 (*Paranthropus aethiopicus*), DNH 7, DNH 155, SK 46, SK 48 (*P. robustus*), and OH 5 (*P. boisei*); early *Homo*: Stw 53 (*H. gautengensis*) and SK 847 (*H.* cf. *habilis*); *H. erectus s.l*.: KNM-ER 3883; KNM-WT 15000; OH 9 (Africa); D 2280, 2282, 2700, 3444, and 4500 (*H. erectus/georgicus*); Trinil 2; Mojokerto; Ngandong 1, 2, 7, and 12; Ngawi 1; Sangiran 17; Sambugmacan 1, 3, and 4; and Skull IX (South-East Asia); *H. floresiensis*: LB1; *H. naledi*: DH3 and Lesedi 1; *H. antecessor*: TD6-15; Middle Pleistocene hominins: Aroeira 3 cranium; Bilzingsleben 7573; HK75 199 and 87; Ceprano; Ehringsdorf H1024; H 1025; LH 18; Sima de los Huesos skulls 5, 12, 13, 15, and 17; Steinheim; Zuttiyeh; Bodo; Broken Hill 1; and Petralona; *H. neanderthalensis*: Amud C1, Apidima 2, La Chapelle aux Saints, Feldhofer, La Ferrassie 1, Forbes’ Quarry 1, Guattari, Krapina 3 and 6, La Quina H5, Spy 1 and 10, and Tabun C1; *H. sapiens*: Jebel Irhoud 1 and 2; LH18; Qafzeh 9; Hofmeyr; Cro-Magnon 1, 2, and 3; Mladeč 1; Pataud; Afalou 2, 12, 13, 28, 30, and 34; and Taforalt XI C1, XV C4, XV C5, and XVII C1. Information about resolution, as well as preservation of the individuals and the pneumatization, is listed in table S2.

The recent *H. sapiens* sample comprises 345 adult individuals from different geographic areas: 78 individuals from Alaska; 48 from Greenland; 71 from the Pacific area; 40 from Spain, 63 from Poland, 9 from China, 11 from India, 5 from Peru, 8 from Mexico, and 12 from Liberia. Computed tomography (CT) or micro-CT was obtained from various sources [for details, see (*6*, *36*, *50*, *51*)]. Extant nonhuman primates include 32 (18 females and 14 males) *Pan paniscus*, 33 (19 females and 14 males) *Pan troglodytes*, and 33 (19 females and 14 males) *G. gorilla* from the collections of the Royal Museum of Central Africa. The latter specimens were all wild, adult individuals (*9*, *21*, *52*).

Three-dimensional (3D) models of the frontal sinuses were created in Avizo 7 (FEI, Hillsboro, Oregon) using manual segmentation with the help of customized settings. Only the preserved parts of the sinuses were reconstructed, and no virtual reconstitution was attempted. Eight linear dimensions were measured as 2D projections in anterior, superior, and lateral orientations (Fig. 2, fig. S1, and table S1). These measurements define the maximal extension of the frontal sinuses in all directions, including bilateral data for the right and left sinuses. In anterior view, we measure the maximal lateral extension of the pneumatization (*W*) and its maximal height (*H*) as well as the maximal length of the right and left frontal sinuses (AL: ALl and ALr). This last distance is quantified from the most medial and inferior point of the sinus to the most distant point of the extension of the sinus vertically and laterally. In superior view, we measured the maximal medio-lateral extension of each sinus (SL: SLr and SLl). In left lateral view, we measured the two sinuses’ length from the most anteriorly protruding point of the two sinuses to the most posterior point in a horizontal direction (AP) and the length from the most anterior point to the maximal supero-posterior extension of the sinuses (AP2). We also quantified the volume of the frontal sinuses, directly obtained from the reconstructed 3D model. These variables were selected because they are easy to visualize in 3D and are therefore less likely to be affected by sinus preservation. All measurements were made by the same observer (A.Ba.). The method has already been tested and validated in a previous study (*9*). We also describe and quantify variation in sinus size within each group following a similar pattern as in our previous work [e.g., (*6*, *9*, *11*, *52*, *53*)] to allow for comparisons with independent studies (Supplementary Text).

Absolute data (unscaled dimensions) for the measurements of the sinuses were compared, and the cube root of the volume of the pneumatization was used for trait size correction to obtain relative values. Size-corrected dimensions were calculated to allow for comparisons of variation in shape. The whole dataset was used for multivariate analyses, including PCA (Figs. 4 and 5). We also compared the variation in size, position, and extension of, respectively, the right and left frontal sinuses and the bilateral variation in the shape of the frontal lobes of the brain (petalias). The anteriormost point on each side is described as the right and left frontal poles following previous experience (*9*).

Considering statistical approaches, we used several different procedures conducted with Past 4.05 software (*54*). We explored the whole information expressed by our results, including results that appear to be “nonsignificant” or “negative” and do not only refer to significance thresholds, as suggested by Amrhein *et al.* (*55*). The coefficient of variation (CV = SD/mean) was corrected for a small sample size using the V* parameter, which is calculated as [(1 + 1/4 *N*) × CV] and expressed in percentages (*56*, *57*). Linear regressions were calculated with the reduced major axis algorithm (*58*), which minimizes the errors in both variables (*59*). Figures for the PCA and linear regression were computed in R (R Core Team, 2014).

## Supplementary Materials

### This PDF file includes:

Supplementary Text Figs. S1 to S70 Tables S1 to S6 References

## Notes

View/request a protocol for this paper from *Bio-protocol*.
