# Supplementary material for: Frontal sinuses and human evolution
Source: Sci Adv. 2022 Oct 21;8(42):eabp9767. doi: 10.1126/sciadv.abp9767 (PMC9586476; doi:10.1126/sciadv.abp9767)
Supplement: Supplementary file 1 — Supplementary Text Figs. S1 to S70 Tables S1 to S6 References [file sciadv.abp9767_sm.pdf]

Science Advances
AAAS

Supplementary Materials for

Frontal sinuses and human evolution

Antoine Balzeau *et al.*

Corresponding author: Antoine Balzeau, abalzeau@mnhn.fr

Sci. Adv. **8**, eabp9767 (2022)
DOI: 10.1126/sciadv.abp9767

This PDF file includes:

Supplementary Text
Figs. S1 to S70
Tables S1 to S6
References

Supplementary Text

Introduction

Frontal pneumatization is not a feature shared by all living primates. Ethmoidally-derived frontal sinuses are present only in *Gorilla*, *Pan*, and *Homo* (7, 8). Moreover, the knowledge regarding variation in size and shape in fossil hominins is limited for several reasons. The pneumatization is difficult to identify directly on original fossils, or only when they are fragmented. The shape and extension of the frontal sinuses appear to show a great amount of variation in *Pan*, *Gorilla* and particularly in *H. sapiens* (6, 9, 20), further complicating its study in the hominin fossil record. Most of the published evidence resides in brief descriptions that appear occasionally in complete descriptions of fossil specimens. The few comparative studies on frontal sinuses to date have been based on restricted samples and have focused on comparing Neandertals to *H. sapiens* and related species, or on the distinctive sinuses of Middle Pleistocene *Homo* (3, 6, 10, 11).

Human paranasal sinuses have been of interest for anatomists for more than a millennium (14). However, researchers still debate their potential function and the correlates of their morphology. Many colourful explanations have been proposed for sinus function (for reviews, see 12-14), from flotation devices (15) to acoustic aids (16). Two enduringly popular functional hypotheses are that sinuses are a climatic adaptation (e.g., 17) or that they serve to disperse masticatory strain (e.g., 18). An alternative is that sinuses have no function at all and are evolutionary spandrels in the sense described by Gould and Lewontin (19). However, there are several sinus types (frontal, maxillary, sphenoidal and ethmoidal) and the available evidence tends to show that they are not functionally and developmentally homologous (6, 20). In this context, we focus here on the frontal sinuses and aim to answer questions about their variability and function.

While the available evidence tends to show that the sinuses are highly morphologically variable in hominins, they have also been used as taxonomically informative characteristics. Sinus presence and morphology have been used in systematics in phylogenetically disparate taxa (1, 2) and there is evidence that pneumatic variation may be diagnostic in Mid-late Pleistocene hominins (3-6). Relationships between hominins remain far from clear and any morphological character likely to be informative regarding phylogeny would be useful. One factor limiting the utility of frontal sinuses in systematic work in hominins, however, is that little information is available for australopiths or early *Homo* (60, 61) and even less exists for earlier hominins. No comprehensive study of pneumatization has been undertaken in *H. erectus*, but evidence (6, 20, 62-68) suggests that specimens have frontal sinuses that may reach similar relative sizes to *H. sapiens* and *H. neanderthalensis* and that there is much variation in this feature. In contrast, extremely large (hyperpneumatized), complex frontal sinuses have been proposed as a distinctive trait in at least some Middle Pleistocene specimens often classified as *H. heidelbergensis* (3-6, 11, 20). Since the first discoveries of *H. neanderthalensis* fossils in the 19^{Th} century, *H. neanderthalensis* have been characterised as hyperpneumatized (69-71), a condition used to explain their large supraorbital tori (17, 44). Recent research, shows that *H. neanderthalensis* are not hyperpneumatized compared to *H. sapiens* in terms of the absolute and relative volume of the pneumatization (6, 10, 35). Maxillary sinus volume has been shown to scale isometrically with craniofacial size in hominoids (72). The question of whether frontal sinuses may also fit this pattern in most hominin taxa is still open. In addition to the less than clear taxonomic differences in frontal sinus size, the question of variation in shape between these different species has not yet been properly addressed, due to the complexity of frontal sinus shape and the extremely high levels of variation within as well as between taxa.

We have two main complementary objectives in this study that allow us to address numerous questions about the origin of frontal pneumatization, its utility for phylogenetic interpretations of the hominin fossil record, the interpretation of how the frontal sinuses relate to the rest of the cranium, and therefore about the anatomical characteristics of hominin groups.

The first objective of this study is to describe variation in the shape and size of the frontal sinuses among fossil hominin species. To quantify variation in size, we measured the dimensions of the frontal sinuses in three planes (x, y, and z Fig. S1 and Table S1). We have developed a simple and repeatable methodology, quantifying sinus size globally, to allow for a very large study. We also describe and quantify variation in sinus size within each group. Frontal sinuses vary considerably in shape, size, position and extension, complicating their quantification (9), but the main limitation is that they are virtually never completely preserved in fossils, particularly in their inferior extension. For these two complementary reasons, a complex quantification including multiple characterizations of smaller details was not possible. It would have induced noisy results, without clear information, and would have concerned very small samples, or would have required a multitude of comparisons using few variables. Our adapted methodology was developed in a previous study (9) and allows for analyses of large samples of *Pan paniscus*, *Pan troglodytes*, *Gorilla*, several geographically diverse populations of *H. sapiens*, and numerous specimens from multiple fossil hominin species. Frontal sinus data from fossil species are not always accessible, because of the limited number of specimens available or low preservation, among other limitations. As much as possible, here we give information about the holotype for each species; for several species, this description constitutes the first direct measurement of frontal bone pneumatization. We also detail data about variations within each group.

Our second objective is to describe and quantify patterns of bilateral variation in the frontal sinuses and investigate the potential relationship between sinus size and shape and the position of the underlying frontal lobes. This allows for a discussion of the origin of the sinus morphology and its variation observed in hominine evolution.

In this context, we bring original information to answer to several longstanding questions in paleoanthropology relating first to the evolution of frontal sinuses in primates and second to their origin as anatomical structures and regarding the factors leading to their shape.

In detail, the specific questions that are addressed are:

1. What is the expression of frontal pneumatization in the first hominins?
2. Can we identify taxon-specific morphology for some hominin taxa and describe the polarity (plesio-/apomorphic status relatively to different levels of classifications) of the observed traits?
3. In relation to the unique information collected for most hominin species, what are the implications of the variation in frontal pneumatization for phylogenetic interpretations of taxa?
4. Can we identify specific frontal sinus shape and size among Middle Pleistocene hominins?
5. Is it plausible that the development of the frontal sinus is related to biomechanical demands, as suggested by previous researchers?
6. Is the size and shape of the frontal sinuses related to the anatomy of the adjacent anatomical areas?
7. Is there any correlation between brain asymmetry and bilateral variation in the frontal sinus development?

8. Do Neandertals (It should be noted that some of the authors prefer the spelling "Neanderthal") have relatively large frontal sinuses?
9. Is the proposed relationship between the pneumatized frontal torus of *H. neanderthalensis* and their adaptation to a cold climate still valid?
10. What is the implication of our synthetized results for future research on hominin frontal sinuses?

<u>Material</u>

Our sample consists of imaging datasets, including a large number of fossil hominins (N = 94; Table S2). These fossils may be separated in different geographic and/or chronological groups: Early hominins (n = 14): *Sahelanthropus tchadensis*, *Australopithecus africanus*, '*A. prometheus'*, *A. garhi, A. sediba*, *P. aethiopicus*, *P. robustus*, and *P. boisei*; Early *Homo* (n = 2): '*H. gautengensis'*, *H. habilis*; *H. erectus s.l.* from Africa and Asia and '*H. georgicus'* from Georgia; *H. floresiensis* (n = 1); *H. naledi* (n = 2); *H antecessor* (n = 1); Middle Pleistocene hominins from Europe and Africa (n = 17); *H. neanderthalensis* from Europe (n =13); and fossil *H. sapiens* dating to 300-25 Ka from Africa, Europe, and SW Asia (n = 19).

The analyses of imaging datasets were complemented by observation of the original fossils for which the state of fragmentation allowed the observation of the maximal extension of pneumatization inside the frontal bone. The literature gave additional information but was of limited use because of the wide variation in the methods used for estimation of the extension of the pneumatization and various levels of detail in those descriptions. Information about the imaging datasets, including resolution and repository, as well as about the preservation of the specimens and of the frontal pneumatization, are listed in Table S2. We were not able to reconstruct, visualise or analyse the frontal sinuses based on the available imaging data for several important fossil specimens (KNM-ER 406, 1805, 1813, 3733, Sangiran 27, Skhul 5). Those individuals are listed in Table S2 where we also explain the reason for this.

For comparative purposes, the recent (defined here as from historic and modern times) *H. sapiens* sample comprises a total of 345 adult individuals from different geographic areas. This sample corresponds to 78 individuals from Alaska; 48 individuals from Greenland; 71 from the Pacific area (New Britain, Solomon Island, Busuango Island, Philippines, New Zealand, Australia); 40 from the Oloriz collection in Spain; 63 individuals from the Polish medieval site of Ostrów Lednicki; 9 from China; 11 from India; 5 from Peru; 8 from Mexico; and 12 from Liberia (these five last groups are all from the Morton collection, see 37). CT or micro CT for these specimens were obtained from various sources (for details see 9, 37, 51) and most of the analyzed data is extracted from our previous work (9). Sex is not known for all of these samples and so was not included as a variable.

We included a large sample of extant non-human primates based on imaging CT datasets, namely 33 (18 female, 14 male) *Pan paniscus*, 33 (19 female, 14 male) *Pan troglodytes*, and 32 (19 female, 14 male) *Gorilla* from the collections of the Royal Museum of Central Africa, Belgium. These specimens were all adult, wild individuals (21, 52). Acquisition parameters varied according to cranial size, as a result pixel size and slice thickness both ranged from 0.3 to 0.7 mm. The complete analyzed samples consist in a total of 537 specimens.

Methods

The method is derived from our study of frontal pneumatization in extant species of the genus *Pan*, *Gorilla* and *H. sapiens* (9). 3D models of the frontal sinuses were reconstructed from the imaging datasets for each individual using manual segmentation with the help of customized settings. We used multiple threshold values as a function of the modifications of the grey values of the tissues. Indeed, the sinuses are sometimes filled with sediment or different materials that need to be virtually removed. The segmentation was performed with Avizo 7 software (FEI, Hilsboro, Oregon). The models obtained were used for qualitative and quantitative analyses of sinus size and shape. We decided to reconstruct and analyze the complete extension of the frontal sinuses within the frontal superstructures and the squama in 3D. The inferior extension of the frontal sinus is not easy to delimitate. Indeed, these sinuses are connected with the ethmoid pneumatization (Fig. S1). The connecting areas between frontal and ethmoid sinuses form the frontal sinus drainage pathway (FSDP), which has no well-defined boundary. Moreover, this area is infrequently preserved in fossil hominins. As a result, we use the frontal ostium as the inferior limit for the frontal sinuses (73). This allows for an easier and repeatable determination of the extension of the frontal sinuses.

To measure the sinuses, each 3D model of the skull is positioned in the Frankfurt plane. The segmented sinus volumes are visualized and measured in anterior, superior and lateral orientations and are comparatively described for all fossil specimens. We follow a similar pattern for the description of the sinuses as in our previous work (6, 9, 11, 52, 53) to allow for comparisons with previous and subsequent studies. For the quantitative analyses, 8 linear dimensions were measured in anterior, superior and lateral orientations (Fig. S1, Table S1). These measurements define the maximal extension of the frontal sinuses in all directions, including bilateral data for the right and left sinuses. In anterior view, we measured the maximal lateral extension of the pneumatization (W), its maximal height (H) and the maximal length of the right and left frontal sinuses (Anterior Length: ALl and ALr). This last distance is quantified from the most medial and inferior point of the sinus to the most distant point of the extension of the sinus vertically and laterally. In superior view, we measured the maximal medio-lateral extension of each sinus (Superior Length: SLr and SLl). In left lateral view, we measured the length from the most anteriorly protruding point of the sinus to the most posterior point in a horizontal direction (AP) and the length from the most anterior point to the maximal supero-posterior extension of the sinuses (AP2). These eight linear dimensions were measured as 2D projections in the different orientations. We also quantified the volume of the frontal sinuses with the specific tool of the imaging software. These variables were selected because they are easy to visualize in 3D and are less likely to be affected by sinus preservation. We quantify the sinuses only for specimens that are sufficiently preserved for a precise characterization of the dimensions. Table S2 indicates which samples are included in which morphological and/or morphometric analyses, depending on their preservation. All measurements were made by the same observer (AB). The method has been tested and validated in a previous study, repeatability was shown to be high (9). Measurement error is indeed a great potential limitation in anatomical studies, particularly when bilateral variation is investigated.

Comparisons of the pneumatic extension between samples concerned variables W and H, the lateral extension of the sinus in anterior (ALl and ALr) and superior (SLl and SLr) views, and their anteroposterior lengths. The lateral extensions were measured separately on the left and right side for later bilateral comparisons. For the comparative analyses, the two bilateral measurements were combined together. The maximal length of the two sinuses in anterior and superior views are labelled 2AL (ALr + ALl) and 2SL (SLr + SLl). We also combined the two antero-posterior

dimensions (AP + AP2) in a unique variable defined as 2AP. Absolute data for these five measurements (W, H, 2AL, 2SL, 2AP) were compared. We also report the summary statistics for the cube-root of the volume of the pneumatization (CRV). This variable was used for trait size correction for subsequent analyses. The calculated size-corrected dimensions (relative to volume of the pneumatization) were also used to allow for comparisons of variation in shape of the pneumatization between groups. The whole dataset, including extant and extinct species, was then used for multivariate analyses, including principle components analysis (PCA).

The data were compared to look at variation between species in the extension of the sinuses including bilateral variation. To determine asymmetry within an individual, we quantified values for (R-L) for AL and SL. Moreover, we compared the results from the present study with those from a previous study of extant species, which analyzed patterns in directional and fluctuating asymmetry in frontal sinus size and shape (9). We also compared the variation in size, position and extension of respectively the right and left sinuses and the bilateral variation in the position and extension of the frontal lobes of the brain (petalias). Data for the sinuses are the quantitative data of this study but we also considered qualitative information observed on fossils. About the endocranial data, the anteriormost point on each side are the right and left frontal poles. The quantitative relationship between the relative position of the frontal poles and bilateral variation in the sinuses in *Pan*, *Gorilla* and *H. sapiens* was previously investigated (9). This approach is not employed with the fossil samples here because of instances of taphonomic alteration, and incomplete preservation of the sinuses, endocasts or skulls, which prevent a quantification of all the necessary parameters on large samples. For this reason, we instead characterized R-L relative position of the frontal poles qualitatively, as described by Holloway and de la Coste-Lareymondie (1982) (74), taking into consideration the anterior and lateral extension of the anterior part of the frontal lobes in superior view. These data were compared with the measurements made on the frontal sinuses as well as with the qualitative assignment of their position via the observation of the relationship between sinus position and petalias. The purpose of this is to look at the possible influence of the position of the frontal poles on the expression of the sinuses. In this context, the shape of the frontal superstructures was also observed and considered at the individual level. Endocranial volume was measured on the 3D models or was from the literature.

Considering the statistical approaches, we used several different procedures that were conducted with Past 4.05 software (55). We explored the whole information expressed by our results, including results that appear to be “non-significant” or “negative”, and do not only refer to significance thresholds, as suggested by of Amrhein et al. (2019) (55). The coefficient of variation (CV = SD/mean) was corrected for small sample size using the V* parameter, which is calculated as $[(1 + 1/4N) \times CV]$ and expressed in percentages (56, 57). Linear regressions were calculated with the Reduced Major Axis algorithm (58), which minimizes the errors in both variables (59). Figures for the PCA and linear regression were computed in R (R Core Team, 2014).

A specific analysis was done to test for spatial autocorrelation and geographic diversity among the extant sample of *H. sapiens*. A PCA was calculated on the whole available metric database for this sample. A Mantel test (75) was then used to test for the presence of spatial autocorrelation on PC1 in relation with latitude and longitude. Next, a generalized linear model (GLM) was used, with PC1 as the dependent variable and the geographical region as the predictor, to test whether the measurements of the frontal sinuses differed between geographic regions. The GLM model was checked in terms of homogeneity violation and outliers using visualization methods (e.g. scatter plots, histograms), tests for outliers, Cooks distances and residual check versus fitted values.

Results

Descriptive anatomy of fossil hominin species

We detail below descriptive and detailed information about the type specimen of each fossil hominin species, where available and possible (i.e., if the holotype preserves the corresponding anatomical area), and about other key fossil specimens for each species. In this way we aim to contribute to the knowledge of frontal sinus characteristics in the majority of hominin species (illustrations are available in Figs. S2 to S70 in vertical, lateral and vertical orientations) as detailed data have been lacking until now for most of these taxa. Comment on the preservation of the relevant anatomical area for each individual is detailed in Table S2.

Sahelanthropus tchadensis.

The holotype TM 266-01-060-1 (also named Toumaï) is the only cranial material available for this species. In this individual (Fig. S2), the frontal sinus completely fills the area between the two orbits and propagates into the massive glabellar area, stopping bilaterally at the level of the supraorbital notch. As a result, the pneumatization fills laterally one third of each frontal torus; it extends further laterally on the right side compared to the left. It continues posteriorly in the thick frontal torus and into approximatively the first third of the frontal squama. Seen from above, the pneumatization reaches the level of the strong post-orbital constriction posteriorly, maintaining a regular lateral extension from the torus to the frontal squama. The sinus continues slightly laterally to the temporal line on the right side. The shape of the pneumatization appears globular, but this may be partly due to the difficult work of segmentation, due to sediment infilling and the lack of preservation of the finest detail of the sinus. However, the general extension of the pneumatization is preserved and the right sinus is globally larger than on the contralateral side.

Kenyanthropus platyops.

In the holotype, KNM-WT 40000, the sinuses are incompletely preserved. Indeed, no information on the internal structure of the medial part of the frontal torus is preserved on the right side (Spoor, pers. comm.) and the glabellar area and medial third of the torus on the left side is lacking (76). The medial border of the preserved part of the frontal bone on the left side shows the most lateral extension of the frontal sinuses. As a result, we can estimate that the pneumatization propagated laterally as far as at the level of the middle of the left orbit (Spoor, pers. comm.).

Australopithecus africanus.

In the Taung 1 holotype, the ethmoid cells are clearly visible, but they do not propagate into the frontal bone. This absence of frontal sinuses is likely explained by the young age of this individual, estimated between 3 and 4 years (77). Frontal sinuses are the last to develop in *H. sapiens* and begins to be visible from an age between 4 and 7 years (78). We have also looked at the condition in a few *Pan paniscus* specimens with the M1 erupted (84036M5, 88041M1, 88041M16), making them of a comparable developmental age to Taung 1, and they do not show any sign of frontal pneumatization. The absence of frontal sinuses in Taung 1 is expected based on the pattern of development observed in extant *Pan*, *Gorilla* and in *H. sapiens*, they likely have developed at an older age.

A similar pattern of pneumatization is observed in all the available adult *A. africanus* specimens (Sts 5, Fig. S3, Sts 71, Fig. S4 and StW 505, Fig. S5). Indeed, we observe that the frontal sinuses occupy the complete interorbital area, propagating laterally as far as around the middle of the orbits, following the shape of the frontal torus. As a result, the protruding glabellar and medial extension of the frontal torus, which are much thicker than its lateral extension, are well pneumatized. The frontal sinuses are therefore globular and massive, spreading around the central volume that fills the large glabellar area. The sinuses continue posteriorly into approximatively the anterior third of the frontal squama.

Australopithecus prometheus.

The holotype, MLD 1, does not preserve the sinuses. The skull of StW 573 (Fig. S6) is complete but partly crushed, particularly in the medial area of the frontal bone. The internal structure is altered in this area, with large cracks and fragments of bone that are displaced. However, we were able to identify most of the extension of the frontal sinuses in this fossil. Their inferior extension is difficult to identify but their propagation in the other directions maybe be delimited with confidence. Their position is also slightly altered due to the general deformation of the frontal bone, but this has limited influence on our quantification of their global shape. Based on the available information regarding the medial borders of the orbits, we consider that the pneumatization filled the interorbital area. The glabellar area is also well pneumatized; the sinuses propagate laterally into the first third of the frontal torus. They continue posteriorly in the area of the post-toral depression and their shape is constrained by the post-orbital constriction. The frontal sinuses have a triangular shape in superior view.

Australopithecus garhi.

The holotype BOU-VP-12/130 (Fig. S7) preserves a good part of the frontal bone including most of the extension of the frontal sinuses. The only direction in which they are incomplete concerns the endocranial surface. However, the maximal extension of the sinuses is visible and quantifiable in all the analyzed directions. The sinuses extend mostly throughout the interorbital area and along the medial wall of the orbits. They propagate vertically in a posterior orientation. Indeed, they do not fill the anterior part of the frontal torus. Their shape is quite globular, with postero-lateral cells that propagate on each side above the medial corner of the orbital roof.

Australopithecus sediba.

The skull U.W. 88-50 (MH1) (Fig. S8) is part of the holotype of this species. MH1 is an immature individual, said to be a sub-adult (79). The cavities for the frontal sinuses are filled with sediment and the internal structure of the bone is slightly altered. We were able to identify the extension of the frontal sinuses in all directions; however, the thin plate of bone that separated the two sinuses is not preserved. The frontal pneumatization is well developed. It has a fan shape and is asymmetric towards the right. It completely fills the interorbital and glabellar areas. It continues laterally as far as the orbital notch on the right side and more laterally above the medial third of the left orbit. The right sinus was much smaller that its left counterpart before the destruction of the dividing septum. Posteriorly, the right frontal sinus is restricted to the large medial part of the torus. On the left side, the sinus continues along the post-toral depression. The sinuses are already large in this non-adult individual; yet some additional development in size was certainly still possible.

Paranthropus aethiopicus.

The holotype for this species is a mandible: Omo 18-1967-18. The best-preserved individual, the adult skull KNM-WT 17000 (Fig. S9), is heavily mineralised but it is possible to assess its frontal pneumatization. On the left side, a small cell is visible at the inferior margin of the frontal bone, it corresponds to the uppermost extension of the ethmoid pneumatization or to the frontal sinus drainage pathway. There is apparently no propagation of the sinus in the frontal bone on that side. On the right side, the sinus is ovoid, it fills the main part of the glabellar area as it propagates to the left of the mid-sagittal plane. It continues to the right into the first third of the frontal torus and posteriorly behind the large, flat post-toral depression, going slightly into the frontal squama. We cannot ascertain if the pneumatization extended further in this individual, due to its preservation. More data is mandatory to have precise information about frontal sinus in this species.

Paranthropus robustus.

The frontal area is not preserved in the holotype, TM 1517, but we could observe the frontal sinuses in several specimens (SK 46, Fig. S10), SK 48, Fig. S11, DNH 7, Fig. S12 and DNH 155, Fig. S13). The individual SK 46 (Fig. S10) preserves only the medial and left part of the frontal bone. The pneumatization has a similar position and extension to that of SK 48, filling the large area available in the centre of the frontal torus, whilst being constrained posteriorly by the postorbital constriction. In the better-preserved SK 48 (Fig. S11), the frontal sinuses completely fill the interorbital area, slightly expanding above the orbits into the frontal torus and into the beginning of the frontal squama, behind the large post-toral sulcus. The left sinus is larger than the right one. In DNH 155 (Fig. S13), the incomplete preservation of the frontal bone means that the details of the shape of the sinuses are not preserved. However, we can estimate their position and extension in all direction with better confidence than for the other fossils from this species. The sinuses fill the large central area of the torus in anterior view both antero-posteriorly and vertically, they also fill the complete thickness of the torus in superior view. Laterally they reach the middle of each orbit. Similarly, DNH 7 (Fig. S12) shows an incomplete preservation but a very good state of conservation. The sinuses are absolutely smaller than in DNH 155. They extend laterally slightly over the midpoint of the orbit. The medial expansion in the vertical orientation is more limited than in DNH 155. The main limitation concerning the antero-posterior dimensions of the sinuses in DNH 7 is that resulting from the relatively small development of the frontal superstructures in this individual. We cannot estimate the original preservation above the left orbit due to incomplete preservation but as whole the pneumatization is smaller than in DNH 155.

Paranthropus boisei.

The holotype, OH 5 (Fig. S14), shows very good preservation of the frontal sinuses. They completely fill the glabellar area and extend laterally beyond the supraorbital notch on both sides. They continue posteriorly into the frontal squama and laterally to the two temporal lines. The pneumatization reaches posteriorly to the area of the post-orbital constriction. As a result, the shape of the frontal sinuses is related to the large surface area and thickness of bone that constitute the medial part of the frontal torus and the large, flat supratoral sulcus. The sinuses have a few small digitations in the glabellar area and at their lateral extent. The rest of the pneumatization constitutes a large triangular cell on both sides, when viewed from above.

Homo gautengensis.

StW 53 (Fig. S15), the holotype, preserves a partial frontal bone whose sinuses are partly exposed anteriorly where the bone is broken. Nevertheless, the overall shape of the sinuses is visible, as well as their maximal extension in all directions. The volume is more affected by the incomplete preservation. For this reason, all linear distances are measured on the obtained volume and are considered as the real maximal extension of the pneumatization of the sinuses in all directions. The volume of the incomplete left sinuses induce a bias in the quantification of the total volume of the sinuses. As a result, we have extrapolated the measured volume of 5600 mm^3 as directly measured on the individual to a volume of 7500 mm^3 based on the size and shape of the missing area compared to that of the sinus on the other side. The sinuses are well developed and appear to be composed of a very large cell with some digitations at its extremities. The sinuses fill the glabellar area and reach the middle of the right orbit and around the same area on the left, based on the shape, size and preservation of the pneumatization in this area. The pneumatization continues posteriorly into the region of greatest thickness of the frontal torus and propagates vertically into the squama of the frontal bone at both its lateral extremities and medially.

Homo habilis.

The holotype, OH 7, does not preserve the frontal bone. Available imaging datasets for the well-preserved individuals KNM-ER 1805 and 1813 do not permit the evaluation of the extension of the frontal pneumatization. SK 847 (Fig. S16), however, preserves the left side of the frontal bone and the corresponding pneumatization. On this side, the sinus probably constituted a unique globular cell. This cell fills the glabellar area and propagates laterally to just above the incurved medial part of the orbit. Posteriorly, the sinus fills the complete antero-posterior thickness of the torus and does not continue into the squama.

H. erectus sensu lato

African fossils attributed to *H. ergaster/H. erectus*.

In KNM-ER 3883 (Fig. S17), the frontal sinus are not very well preserved but extend throughout the whole glabellar area and laterally into the anterior-most two thirds of the frontal torus. The sinuses fill the post-toral area posteriorly. It constitutes a large volume because of the massive shape of the central area of the torus and its considerable antero-posterior extension. The immature individual KNM-WT 15000 does not preserve the anterior part of the frontal torus, but the pneumatization is partly visible in its posterior extension. The pneumatization extends laterally nearly as far as the lateral third of the frontal torus. Posteriorly, the sinuses reach the level of the beginning of the frontal squama. As a result, the expansion of the sinuses in this young individual is as large as in the adult KNM-ER 3883. An increase in sinus volume was also clearly possible in this immature specimen. In OH9 (Fig. S18) only the upper most extension of the sinuses is visible. They extend laterally above the orbital notch on both sides. They constitute massive volumes and probably invaded the entire, large glabellar area.

Georgian fossils attributed to *H. georgicus/H. erectus*.

In D2280 (Fig. S19), the frontal bone and the frontal sinuses are well preserved, except for some parts of its infero-posterior extension. The sinus fills the interorbital area and continues into the medial part of the frontal torus, extending particularly below the post-toral depression in the central third of the torus, where its antero-posterior thickness is particularly marked. In D2282 (Fig. S20),

the frontal bone is incomplete inferiorly, exposing the sinuses, whose complete extension is preserved. The sinuses constitute a large globular cell on each side, filling the interorbital area and the antero-posterior extension of the frontal torus in this area. In D2700 (Fig. S21), a small cell propagates just below the inferior part of the frontal bone on the left side, but there is no trace of true propagation of the frontal pneumatization. We also observe the remnant of a metopic suture on 1.5 cm vertically in the glabellar area, as revealed by the imaging dataset. In D3444 (Fig. S22), the frontal sinuses form two large cells that fill the medial part of the frontal torus, as observed in D2280 and 2282. In D4500 (Fig. S23), the frontal sinuses are much larger. In this individual, the sinuses also completely fill the interorbital area and the antero-posterior extension of the very large frontal torus. The right sinus, which is much larger than the left one, continues laterally as far as the mid-orbit and latero-posteriorly in the direction of the area of post-orbital constriction. The left sinus forms an elongated cell. The right sinus is massive, with an uppermost extension that is flat and stretched medially, posteriorly and laterally.

Asian *H. erectus*.

In the Trinil skullcap (Fig. S24), the holotype of *H. erectus*, the frontal sinuses are incomplete and exposed at the inferior border of the fragmented frontal torus. Its lateral propagation corresponds roughly to half the size of the frontal torus on each side, approximately as far as the middle of each orbit. The superior extension of the sinus forms large, globular cells. Posteriorly, the shape of the sinus follows the shape of the endocranial surface closely.

In Mojokerto, the frontal sinus is visible on the left side as a thin space extending from the glabellar area to above the medial extension of the orbit. This individual, aged 2-3 (41, 80) is the unique very young child available for *H. erectus*. Such sinus extension enters the variation observed at the same age in *H. sapiens*.

In Sangiran 17 (Fig. S25), the frontal sinuses fill the glabellar area and extends within the medial third of the frontal torus on both sides. It continues posteriorly and borders the endocranial surface posteriorly but does not propagate into the frontal squama. On the left side, the sinus is a single cell, much larger than the two cells seen on the right side. The sinus has a large volume because of the large area available within the glabella and the thickness of the frontal torus antero-posteriorly, which are extensively pneumatized. In Skull IX (Fig. S26), the frontal sinuses have a similar shape and position but are of smaller size.

In Sambungmacan 1 the bone structure is altered by taphonomic changes, likely effecting the pneumatization. In Sambungmacan 3, the exposed surface at the inferior breakage of the frontal torus and the imaging dataset permit us to conclude that this individual had no frontal sinuses. In Sambungmacan 4 (Fig. S27), the extension of the sinuses is quite considerable, similar to that observed in Sangiran 17. The left sinus is larger than the right one. The left sinus is composed of two connected globular cells, the first one fills the glabellar area and the second one the medial third of the torus. The two sinuses follow the outline of the endocranial surface, filling the thick torus and do not continue into the frontal squama.

The frontal sinuses have a similar shape and size in Ngandong 1 (Fig. S28), Ngandong 7 (Fig. S29) and Ngandong 12 (Fig. S30). They constitute two separate large globular cells, one on each side, filling the medial third of the frontal torus. There is no propagation into the frontal squama. No frontal sinus is yet present in the immature (aged between 6 and 11 years, according to Antón, 1997 (80)) individual Ngandong 2. This lack of pneumatization is compatible with the developmental age of this individual. The Ngawi 1 (Fig. S31) individual shares the same position and extension of the pneumatization as observed in the adults from Ngandong.

The fossils from Zhoukoudian have disappeared but casts and the detailed work of Weidenreich (1943) (62) permit us to detail the characteristics of this sample. Only Skull III (Ckn.E 1 PA. 16) has real frontal sinus cavities. They are globular in shape, relatively large, and located in the peri-glabellar region and medial part of the torus. In most individuals (skulls II, X, XI, XII), the pneumatization is limited to ethmoido-frontal cells and does not spread vertically in the glabellar region.

Homo floresiensis.

Only the skull of LB1, the holotype of the species is available. We have already shown (53) that while the interorbital area is externally damaged, the internal structure of the bone is preserved. It is clear that the ethmoid cells do not continue inside the frontal bone. As a result, frontal sinuses are absent in LB1.

Homo naledi.

The area of the frontal sinuses is not preserved on the DH1 holotype. On DH3 (Fig. S32), only the lateral extension of the pneumatization is visible in the left side of the frontal bone, this side being the only one preserved. The frontal sinuses probably filled the whole glabellar area and the thick antero-posterior extension of the frontal torus in this area. A large cell slightly propagates into the more medial extension of the torus. Medially and posteriorly, another large cell continues into the inferior part of the frontal squama. Based on the size and position of these two preserved large cells, the left sinus was propagating on the right side, and was probably larger than the right sinus. In Lesedi 1 (Fig. S33), the complete extension of the frontal sinuses is globally well preserved. The pneumatization is larger than in DH3. The sinuses invade the intraglabellar area and propagates laterally as far as the centre of the orbits on each side. It fills the antero-posterior extension of the torus and continues into the frontal squama. On both sides the sinus is composed of a large cell divided at its extremities into large digitations.

Homo rhodesiensis.

The frontal pneumatization is particularly developed in the Broken Hill 1 individual (Fig. S34) (3, 10, 11), holotype of the species. It forms two large volumes of cells, separated medially by a thin septum. The frontal sinuses fill the large supraorbital torus and extend laterally to beyond the mid-point of both orbits. The sinuses continue posteriorly far into the sloping frontal squama towards bregma. The overall shape of the frontal sinuses is fan-like, but is made up of many small globular, coral-like extensions filling the entire depth of the bone thickness. Posteriorly, the shape and orientation of the pneumatization follows the global shape of the endocranial surface. The lateral and latero-posterior extension is slightly larger on the right side than on the left side. Moreover, the septum that separates the two sinuses deviates toward the right in the supero-posterior part of the pneumatization. As a result, the right sinus is larger than the left one. For an in-depth investigation of the internal cranial morphology of Broken Hill 1, including the sinuses, see 11.

In Bodo (Fig. S35), the frontal pneumatization is extensive, completely filling the glabellar area, reaching the lateral third of the torus on the right side and the middle of the orbit on the left side. It continues posteriorly into the frontal squama. It has a general shape that is similar to that observed in Broken Hill. The sinus is very asymmetric, the left side being much larger than the

right one. Moreover, it propagates medially and toward the right, a cell of the left sinus expanding above the smaller right frontal sinus. This may further extend the reduced development of the pneumatization on the right side.

The frontal sinuses of Petralona (Fig. S36) constitute the largest volume observed in our samples, including all fossil hominins and the genera *Pan* and *Gorilla*. The sinuses completely fill the supraorbital torus. The pneumatization has large lateral and antero-posterior extensions into the considerable thickness of the torus in these orientations. On each side, a large cell fills the glabellar region. Other cells continue in all orientations, giving a fan-like shape to the pneumatization. The sinuses continue into the frontal squama, reaching the center of the bone on the left side. The overall shape is similar to that of Broken Hill and Bodo.

Homo antecessor.

The holotype is a fragmentary mandible. TD6-15 (Fig. S37) is an incomplete frontal bone where the pneumatization is exposed and whose left extension is probably incomplete. However, most of the shape and extension of the frontal sinuses may be described with confidence. The sinuses form one large globular and elongated cell on each side. On the complete right side, the sinus reaches the middle of the orbit laterally. On this side, the shape of the sinus follows the shape of the torus. The pneumatization fills the antero-posterior extension of the frontal torus. It extends slightly into the frontal squama, where the medial extension of the left sinus also forms a large vertical digitation. The sinuses are already age at this developmental stage in *H. sapiens*, but further developmental would have certainly remained possible in this fossil individual.

Middle Pleistocene *Homo*.

The fossils usually attributed to this group show a great variation in the expression of their frontal sinuses (3, 4, 6, 10, 11). We detail here the features observed in individuals that are grouped in this group in a loose sense (with the notable exception of Broken Hill 1, the holotypes for *H. rhodesiensis*, as well as Petralona and Bodo that were described previously as they share a particular morphology of the frontal sinuses), with some documented cranial differences and originating from different geographical areas.

Bilzinglseben 7573 (Fig. S38) preserves a fragment of the frontal bone. The left frontal sinus is exposed medially due to the incompleteness of the bone. It has a large medial cell that is probably located in the medial third of the frontal torus. It is elongated posteriorly and continues slightly into the frontal torus. A small lateral cell propagates along the internal surface of the frontal bone. Based on this preservation, we suggest that the sinus on this side was fan-shaped and well developed in the glabellar area. The frontal fragment HK 87 preserves the right side of the bone, including the torus. The pneumatization is visible at the medial preserved extension of this fragment. It corresponds to a sinus extending just above the medial wall of the orbit. These two fragments might belong to the same individual.

The individual with the best preservation for the observation of the frontal sinuses is HK75 199 (Fig. S39). This fragment corresponds to the medial part of the torus. It is highly pneumatized but the sinuses are very asymmetric. The left frontal sinus forms a unique cell, slightly propagating vertically into the frontal bone. However its lateral extension is not preserved, it is likely that it continued above the orbit inside the torus. The right sinus is much larger, filling the glabellar area

and propagating in the medial part of the torus on both sides. The pneumatization is massive, with several digitations at all extremities and no apparent structure. A large lateral cell is isolated on the right side, propagating from the base of the frontal bone.

In Ceprano (Fig. S40), the frontal pneumatization forms two globular clusters of a few cells on each side that are restricted to the glabellar area, slightly expanding into the medial part of the torus only on the left side.

The preserved areas of Ehringsdorf H1024 (Fig. S41) border the extension of the frontal sinuses, even if they are partly exposed on the exocranial surface on the right side. The right sinus is much larger than the left one. It propagates until the center of the torus on the right side and only to its medial border on the left. It fully fills the glabellar area. The pneumatization continues slightly in the frontal squama. Ehringsdorf H1025 is a fragment that includes the lateral half of the frontal torus. The frontal sinus did not extend laterally as far as the preserved area.

In Steinheim (Fig. S42), the internal structure of the frontal bone is altered by some cracks and the presence of sediment. Due to those diagenetic modifications, the extension of the frontal sinuses is not clearly identifiable. Their lateral limit is exposed on the left side by the incompleteness of the frontal torus. For this reason, we cannot estimate the lateral propagation of the sinus. It completely fills the interorbital area and continues on both sides above the medial border of the orbit. Posteriorly, the sinuses are contained within the frontal torus and do not continue into the frontal squama.

In Zuttiyeh (Fig. S43), the sinuses form a globular pneumatized area of a cell with some large apical digitations. The right sinus propagates medially while the left sinus continues posteriorly into a smaller cell. The left sinus has an anterior large cell, with a fan shape and two lateral digitations, and a posterior cell dissociated from the most inferior extension of the sinus. The shape of the anterior cell follows the shape of the frontal torus. The lateral digitation continues into the medial part of the torus while the medial digitation follows the shape of the depression in the interorbital area, above the torus. The pneumatization continues laterally until the level of the orbital notch on both sides. It fills the glabellar area and does not continue into the frontal squama.

In Aroeira (Fig. S44), the frontal sinuses are large, filling the complete interorbital area and propagating above the orbits. The left sinus propagates medially but has a more restricted lateral extension above the medial third of the orbit, while the right frontal sinus extends to the mid-orbit. The sinuses fill the antero-posterior thickness of the torus. The sinuses are massive with several large digitations at their vertical and lateral extensions.

In the la Sima de los Huesos sample, 5 adult individuals are available and exhibit some variation in frontal pneumatization. The frontal sinuses are larger in skulls 5 (Fig. S45), 12 (Fig. S46) and probably 15 (Fig. S48), and smaller in skulls 13 (Fig. S47) and 17 (Fig. S49). In all the specimens the right and left sinuses are well separated. They propagate along the medial wall of the orbit and stop vertically slightly above the upper limit of the orbit in skulls 13 and 17, filling only the medial and inferiorly inclined portion of the frontal torus. In these two individuals the glabellar area is not pneumatized. In skulls 5, 12 and probably 15, the sinuses fill the glabellar area, the antero-posterior extension of the frontal torus and approximately the central third of the frontal torus as considered from one side to the other. The more pneumatized individuals show the presence of the sinuses under the supratoral sulcus, but none exhibit propagation into the frontal squama. Globally, the features observed in the Sima de los Huesos skulls resemble what is seen in *H. neanderthalensis*, although the Sima de los Huesos sinuses are more separated right and left than some of the later.

Homo neanderthalensis.

In the Feldhofer holotype (Fig. S50), the frontal bone pneumatization is completely preserved. The two sinuses enlarge above the frontal sinus drainage pathway. They fully fill the glabellar area. Laterally, the right sinus continues along the first half of the orbit while the left sinus propagates only into approximately the first third of the orbit. Posteriorly, the pneumatization does not reach the frontal squama. Its postero-lateral extension follows the shape of the endocranial surface.

Our *H. neanderthalensis* sample shows limited variation in terms of shape, size and volume of the sinuses. Amud 1 (Fig. S51), Apidima 2 (Fig. S52), La Ferrassie 1 (Fig. S53), La Quina H5 (Fig. S54), Forbes' Quarry 1 (Fig. S55), Guattari 1 (Fig. S56), Krapina 3 (Fig. S57) and 6 (Fig. S58), La Chapelle-aux-saints (Fig. S59), Spy 1 (Fig. S60) and Spy 10 (Fig. S61) and Tabun C1 (Fig. S62) share the general pattern of frontal pneumatization described for Feldhofer. The sinuses fill the large space available in the medial half of the large frontal torus. Frontal sinuses invade this medial area of the torus antero-posteriorly and have a cauliflower shape in anterior view. The sinuses may posteriorly slightly extend in the frontal squama, such as in La Ferrassie 1 or Spy 1.

Homo sapiens.

There is no holotype for *H. sapiens*. High levels of variation in frontal pneumatization are documented in the medical and forensic literature (81-85). Here, we have observed, described and measured the sinuses of 345 recent *H. sapiens* individuals from different origins. 41 (12%) do not have sinuses. Aplasia is defined as the absence of cavities above the frontal ostium that is considered as the inferior limit for the sinuses. 9.6% have a unilateral pneumatization (24 have no sinus on the right side and 9 no sinus on the left side), and in most cases the sinus on the contralateral side has a position and size that partly compensates for the absence of the other sinus. Some variation between geographic samples is visible in terms of extension of the pneumatization.

We were also able to study several key fossils attributed to *H. sapiens*. Jebel Irhoud 2 has no frontal pneumatization. Jebel Irhoud 1 (Fig. S63) has large frontal sinuses that fill the glabellar area and continue on both sides above the medial third of the orbits. The pneumatization continues posteriorly into the frontal superstructures and slightly into the frontal squama. LH 18 (Fig. S64) has two well-separated and quite small sinuses that constitute a globular cell on each side propagating along the medial wall of the orbits. In Qafzeh 9 (Fig. S65), the sinuses are very large, particularly laterally as they invade the well-developed frontal superstructures. However, the sinuses have a limited antero-posterior extension, being limited by the shape of the frontal bone as the frontal superstructures are not very developed anteriorly. In Hofmeyr (Fig. S66), the sinuses form two small cavities in the medial part of the supraorbital area. In this individual, the anterior part of the glabella presents a remnant of the metopic suture. In Cro-Magnon 1 (Fig. S67), the sinuses are large, the left being larger. They extend on both sides laterally above the orbital roof and posteriorly into the frontal squama. They are fan-shaped from their base towards their uppermost extension where the cells become individualized. Their shape remains simple, with few larger apical cells. The cells are separated by very thin septa, as are the two sinuses. CM 2 (Fig. S68) has much smaller sinuses, which are well-separated and restricted to the glabellar area. The pattern is similar in CM 3 (Fig. S69) and in Pataud (Fig. S70) for the size and extension of the sinuses. Mladeč 1 does not have any propagation of the pneumatization in the frontal bone. Among our Epipaleolithic sample, which comprises 10 individuals, Taforalt XI C1 and T. XV C4 have small sinuses and Afalou 13, Af. 28 and T. XVIIC1 no frontal sinuses.

Concerning other hominin species, several do not preserve any known frontal bone to date. In this context, no information is available for *Orrorin tugenensis*, *Ar. kadabba*, *A. bahrelghazali*, *A. deyiremeda* and *H. luzonensis* for the moment. We were not able to access the existing fossil material for *Ar. ramidus, A. anamensis* or *afarensis*.

Synthesis about *Pan* and *Gorilla*

A synthesis of descriptive information is also provided for the extant samples of *Pan paniscus, P. troglodytes* and *Gorilla*. Detailed information is presented in a previous study (9).

Pan paniscus.

In the holotype, RG 9338, there is great bilateral variation in the expression of the frontal pneumatization. The left frontal sinus is small, with a volume of 1.6 cm^3. Its extension is restricted to the interorbital area along the medial wall of the orbit. This sinus extends slightly into the frontal torus, just near the endocranial surface. This limited extension is related to the considerable development of the right frontal sinus, which has a volume of 7.4 cm^3. It fills the glabellar region and extends into the anterior part of the torus on both sides. Laterally, the sinus nearly reaches the center of the right orbit and goes into the medial third of the left orbit. The right sinus is composed of one large cell, divided in its posterior extension by small bony walls. The resulting smaller digitations propagate slightly into the frontal squama. Overall, in the complete sample of *Pan paniscus*, the sinuses are mainly located in the glabellar area, quite globular with a triangular shape seen from above, but some variation is visible in the shape and size of the sinuses.

Pan troglodytes.

The sinuses are in most cases quite simple in shape, with only a few rare additional cells that derive from a unique large cell. Occasionally cells form at the extremity of the sinuses in specimens with large pneumatization. Across the species, the sinus shape is triangular in anterior view, with a large cell expanding vertically and laterally. The shape is also triangular in superior view and quite globular in lateral view. The pneumatization fills the large space available in the frontal torus. It expands into the glabellar area, continues along the medial third of the torus laterally and into the area of the post-orbital sulcus posteriorly. When the sinuses are large, their shape is less globular, but more fan shaped, forming a thinner and flatter extension posteriorly. In the few individuals with very large sinuses, anterior and postero-lateral cells are separated from one another.

Gorilla gorilla.

Gorillas have the largest sinuses among extant samples in terms of absolute volume and size in all directions. Gorillas are also remarkable for the lateral and antero-posterior extension of their pneumatization. The sinuses on both sides form a large cavity that is globally flat and elongated in anterior view, compact and antero-posteriorly elongated in lateral view and of triangular shape in superior view. This morphology is related to the shape of the frontal superstructures. A large available area allows the antero-lateral expansion of the sinuses into the torus. Laterally the sinuses continue as far as the middle of the torus, with some variation. Posteriorly, postorbital constriction constrains the sinuses. Globular cells tend to individualize at the extremities of the sinus, particularly in the specimens with larger pneumatization. When the pneumatization is very large, it tends to separate into a larger antero-medial cell that continues laterally and posteriorly into another large cell. Gorillas also present considerable pneumatization of the ethmoid bone that is connected to the frontal sinuses, forming a huge pneumatized area in their medial face.

General morphometric comparisons of the complete sample

The relationship between the cube-root of the volume of the frontal sinuses and the cube-root of the endocranial volume is informative regarding global variation in size (Fig. 2). We observe a significant correlation between frontal sinus size and endocranial size within the complete *Pan* and *Gorilla* sample analyzed here (slope = 1.36, r = 0.72, p = 10e-16), whereas the correlation is not significant when recent *H. sapiens* is included. The pattern within each species is different as only gorillas show a significant correlation between those dimensions (slope = 1.16, r = 0.45, p = 0.009). When fossil hominins are considered, we observe a border-line significant relationship between frontal sinus and endocranial volume and wide variation in sinus size independently of brain size (slope = 2.17, r = 0.089, p = 0.53). Among fossils hominins, the relationship is not significant (slope = 2.27, r = 0.095, p = 0.51). The variation in the volume of the frontal sinuses in hominins encompasses the variation observed in *Pan* and *Gorilla* with a few exceptions. Broken Hill 1 is at the upper extreme of variation seen in *Pan* and *Gorilla*, only one *Gorilla* specimen has larger sinuses, while Bodo and Petralona are the two individuals with the largest sinuses in the entire sample. In this context, fossil hominins with small brain size plot within the variation observed for *Pan* and *Gorilla*. This is the case for TM 266-01-060-1 (*Sahelanthropus tchadensis*), Sts 5 and 71, StW 505 (*A. africanus*), StW 573 (*A. prometheus*), Bou-VP-12/130 (*A. garhi*), U.W. 88 (*A. sediba*), DNH 155 (*P. robustus*), OH 5 (*P. boisei*). Two *Homo* individuals also plot within this variation, LES 1 and D4500. This would be also the case of early *Homo* fossils if we could measure both their endocranial size and the volume of their frontal sinuses.

We also present individual values for the fossil hominin individuals analyzed (Table S3). Table S4 provides a morphometric comparison between the extant samples analyzed and our previous study (9). Principle components analysis (PCA) shows that there is good separation between the different groups. This is related first to size, but also to shape (Table S4), particularly in the antero-posterior and lateral dimensions, which separate out gorillas from the other samples and in the vertical direction, which separates out *H. sapiens* from the other samples. *Pan* and fossil hominins have intermediate positions. As a result, this PCA computed on the absolute data for all the variables illustrates the strong influence of frontal sinus size. PC1 and 2 represent respectively 90.53% and 6.11% of the variance and all variables have high positive loadings on the first axis.

A PCA computed on relative (size-adjusted) data discriminates well between *H. sapiens* and *Pan* and *Gorilla*; while again fossil hominin species have intermediate positions. The distribution of each extant group and fossil hominin species partly overlap each other but some differences are visible. A closer look at the fossil hominin sample gives interesting results. Early hominins, including Toumaï (*Sahelanthropus tchadensis*), plot at the intersection of the distribution of *H. sapiens* and of non-human primates. *H. erectus s.l.* are distributed closer to the *H. sapiens* sample. *Homo neanderthalensis* and *H. heidelbergensis* have respective distributions that only partly overlap and both are subsumed inside the variation observed for *H. sapiens*. Three individuals have a very particular position due to the large size of their pneumatization. Broken Hill, Bodo and Petralona plot outside the distribution of all the other individuals on the first axis.

The position of individual fossil specimens in these analyses needs to be considered with caution. Indeed, incomplete preservation or alteration of the shape of the sinuses may influence the measurements and influence where they plot. That is why we have considered above the position of hominin samples by taxon. However, we can consider the individual positions of key specimens

while taking account their preservation. It is also useful to look at individual specimens because of taxonomic uncertainty, e.g., middle Pleistocene hominins potentially contains several taxa. We have for example split in two this sample based simply on the morphology of frontal sinuses.

Interestingly we had access to several early hominins with a sufficient preservation to describe and compare their pneumatization. Toumaï, the holotype for *S. tchadensis*, plots comfortably with other early hominins and is at the extreme of variation on PC1 for the entire fossil hominins sample for the relative size PCA in relation to a relatively larger antero-posterior extension of the sinuses and a more restricted vertical and lateral extension. StW 573, *A. prometheus*, is well within the distribution of the rest of the early hominin specimens, including the *A. africanus* sample for the multivariate analyses. The *A. sediba* individual, U.W.88, also plots within this variation. Lesedi 1, attributed to *H. naledi*, is in the center of the distribution of *H. erectus* for both the analyses with absolute and relative data. The *H. antecessor* individual, TD6-15 plots well within the distribution of *H. heidelbergensis* for both absolute and relative data analyses. This individual has smaller sinuses than the *Homo neanderthalensis* individuals, according to its position in the PCA obtained from absolute data, while it is within the range of their variation for relative data. This variation in size maybe related to its developmental stage and possible remaining increase in sinus volume during growth.

Morphometric trends between *H. sapiens* populations

When an LDA is computed on the dataset of absolute measurements for the different geographic samples of recent *H. sapiens*, the resulting confusion matrix shows a proportion of correctly classified individuals of 33.9%. This low level of correct classification illustrates the large variation observed within samples and a lack of geographic partitioning in sinus size / shape. A MANOVA, however, shows some significant differences between pairs of samples that were investigated through additional analyses. The resulting squared Mahalanobis distances (Table S5) highlight some closer affinities and differences between groups. Finally, we tested for a potential correlation between sinus dimensions and geography. To do so, a PCA was calculated on the measurement data for the different samples for extant *H. sapiens*. PC1 accounts for 91.95% of the variation. The data (PC1) was tested for spatial autocorrelation using a Mantel test. The statistical analysis (Mantel test: p-value= 0.1662) shows a lack of spatial autocorrelation, which means that the frontal sinus measurements in one population are not more similar to other groups nearby than they are to groups at a greater distance. We also calculated a generalized linear model (Table S6) to see if individuals from various regions differed in the measurements of their frontal sinuses These results illustrate that, though the Mantel tests show that the dimensions of the frontal sinuses are not spatially autocorrelated, they do differ significantly between geographic regions.

Bilateral variation in sinus and brain anatomy

For the dimensions of the frontal sinuses, we observe a tendency toward the left, and significant directional asymmetry toward this side within the complete *H. sapiens* sample. Among the 345 individuals analyzed, 41 do not have sinuses, 24 have no sinus on the right side and 9 no sinus on the left side. In addition, the mean values for (R-L) for the anterior length (AL) and superior length (SL) of the sinuses are -2.23 mm and -2.25 mm. Those mean values are significantly different from zero ($t = -4.3113$, $p = 2.2e\text{-}05$; $t = -3.806$, $p = 0.0002$ respectively). These results illustrate that the

frontal sinuses in *H. sapiens* tend to be larger on the left side than on the right side of the skull as mean (R-L) is negative and significantly different from zero. We have previously shown [9], that none of the quantified traits exhibit directional asymmetry in *Pan* and *Gorilla*.
The comparison of the different analyzed features of bilateral frontal sinus morphology in fossil hominins is difficult. Indeed, several fossils do not have fully preserved pneumatization and taphonomic alteration may alter the shape and size of the sinuses on each size, complicating the analyses of subtle bilateral differences. Small sample size for hominins also prevents largescale analyses of directional and fluctuating asymmetry. This is why we consider here several features at the individual level among our fossil hominin sample. Those data include the bilateral variation of the dimensions of the sinuses when possible, the shape of the torus –described qualitatively- and the position of the underlying frontal lobes, as explained in the methods section, in order to identify potential relationships between anatomical features and repeated patterns among hominin samples. Individual values were calculated for the R-L differences in anterior length and superior length and compared with the other anatomical data observed on each individual. We detail below the resulting qualitative data for fossil hominin individuals.

In the *A. africanus* individuals Sts 5 and StW 505, there is a left frontal petalia of the brain, meaning that the left frontal pole has a more anterior position than on the right side and that the first frontal convolution extends more laterally on the left side than on the right side. This is associated with a smaller frontal sinus on the left side compared to the right sinus. In U.W. 88 (*A. sediba*), a right frontal petalia is associated with a larger left frontal sinus. We observe a tendency among our sample of Asian *H. erectus* for the left sinus to be larger than the right sinus. In these fossils, the frontal torus is very large, while the sinuses form a globular cavity. Most *H. erectus* individuals show a right frontal petalia. The only exception is Sangiran 17, which has a left frontal petalia. In *H. heidelbergensis s.l.*, the left frontal sinus tends to be larger than the right sinus. Moreover, a right frontal petalia is observed in 12 specimens where this feature is observable. Nevertheless, the direct observation of the potential relationship between these two anatomical features does not seem to support a general pattern among the sample. The qualitative observation of those traits show that their respective variation is large and bilateral differences do not exhibit clear qualitative relationship. A slight relationship is visible in SHS 12 (Sima de los Huesos), Ceprano, Broken Hill and Zuttiyeh. In the other individuals, no link is evident in the expression of bilateral variation in the sinuses and the petalias. In *Homo neanderthalensis*, there is no clear asymmetric tendency in the sinuses at the scale of the sample, but incomplete preservation in many individuals may explain this. A right frontal petalia is the most common pattern in brain morphology. Moreover, we observe a clear relationship between the position of the frontal lobes and the extension of the sinuses in La Ferrassie 1, Forbes' Quarry 1, Krapina 3 and Spy 1.

Additional information about frontal pneumatization from the literature

A limiting factor in this analysis of complex internal anatomical traits during human evolution is the available information for the hominin fossil record. Fossil preservation and relatively low resolution for the many of the imaging datasets are indeed problematic. Data access is another issue. Fortunately, we had here access to a unique database to study the variation and evolution of the hominin anatomy, yet there are still taxa we were unable to access. The sample for this study is more complete and diverse in terms of hominin species and fossils individuals included than any

previous study on paranasal pneumatization. The internal preservation of the crania and the capacity of the imaging data allow visualisation of the features studied have to be considered, nevertheless. Our simple and pragmatic protocol allows for a large, precise, and detailed study of this complex fossil record. In this context, we revise previous incomplete or erroneous characterisations of sinus morphology for some fossil individuals or species and obtain original information on the majority of the material.

Additional available information about frontal sinus shape and size, particularly for the hominin species we could not include in this study, is limited and not easy to compare with our detailed morphological and morphometric data. We do not pretend to give an exhaustive overview of the literature but below we discuss some aspects that pertain interestingly to our study.

The description of *Ar. ramidus* (86) mentions that the lateral extension of the sinus is visible at the break of the preserved part of the frontal torus. According to these authors, presence of a sizable sinus in *Ar. ramidus* is shared with chimpanzees and gorillas. However, as shown here some differences exist in frontal pneumatization between those two extant primate genera.

No information is available yet about frontal pneumatization in *A. anamensis* (87). According to Kimbel et al. (2004) (88), the frontal sinuses are variable in *A. afarensis*. According to these authors, the frontal sinuses appear to be poorly developed in the individual A.L. 438-1b, forming small cells at the inferior extent of the bone, whereas in A.L. 444-2 the sinuses completely fill the glabellar area, extending laterally into the orbital roofs and posteriorly in the frontal squama, as observed from fractures on the original specimen.

A detailed description of the sinus of a *P. boisei* individual KNM-ER 23000 is provided in Brown et al. 1993 (89). The sinuses are large and according to the description and the published images, they seem to be similar in shape to those of OH 5 described here. In terms of other *P. boisei* individuals, KNM-ER 733 has tiny sinusal cavities according to Leakey and Walker (1973) (90) and in KNM-ER 407, the frontal torus is absent, but the sinuses propagate into the anterior part of the frontal squama. The lateral parts of the sinuses of this individual extend further posteriorly compared to their extension medially, ending at 45 mm from bregma (91). The individual KNM-ER 814 is a fragment of the right supra orbital marginal that indicates the presence of a large sinus with a position similar to that observed in other *P. boisei* specimens described here. The sinuses are exposed by breaks of the exocranial area in KNM-WT 17400 (92), and they are extensive. Photographs of this individual show that the pneumatization is very similar in position, shape, and size to that we have observed in OH5 (Fig. S14). According to Tobias (1967: p. 212) (93): “The frontal sinus of *Zinjanthropus* is extensive. Like that of *Gorilla*, it has lateral and posterolateral diverticula, but its relationship to the naso-orbital region and to the ethmoidal sinus system cannot be determined. In its extent, the frontal sinus of *Zinjanthropus* is more comparable with that of pongids than with that of *H. erectus*”. This observation is confirmed by our multivariate analysis, indeed OH 5 fits comfortably within the range of variation observed for gorillas for absolute and relative dimensions of the frontal sinuses (Fig. 1). It would be particularly interesting to evaluate the frontal sinus of KNM-ER 733 using digital imaging data to confirm its supposedly restricted pneumatization and compare it with our multivariate dataset. Moreover, the inclusion of other fossils such as KNM-ER 732 would be interesting to evaluate potential patterns of variation in the frontal sinuses related to sexual dimorphism. We have previously observed differences in the size of the sinuses between males and females in gorillas, together with a significant relationship between sinus size and endocranial volume (9).

Available information about early *H.* specimens is limited. In KNM-ER 1470, "there is extensive frontal sinus formation, and matrix that fills the frontal sinus cavity extends 42 mm posteriorly from the left orbital margin and 35 mm laterally from the midline" (91, p. 73). In OH 16, the frontal sinuses appear to be quite small (93). As a result, Tobias (1991, table 189, P. 761 (93)) summarizes the frontal sinus morphology as small with a simple shape in *H. habilis* and *A. africanus*, compared to large and with diverticula in *P. robustus* and *boisei*. Our results give a slightly different view. Sinuses in both *A. africanus* and *Paranthropus* species are relatively large compared to skull size, which may be related to the available space in the glabellar area. Moreover, the two early *Homo* individuals we could analyses: StW 53 and Sk 847, appear also to have large sinuses. All these hominins exhibit sinuses with apparent digitations at their extremities.

As observed in our results, greater variability in frontal sinus size and shape appears with later *Homo* species. In terms of additional information from African *H. erectus s.l.*, the sinus is filled with sediment in KNM-ER 3732, the only available information is that the sinus extends at least 23 mm posteriorly from the orbital margin (91). This posterior extension is similar to that observed for KNM-ER 3883 (Fig. S17). The images published for Daka (67) illustrate that the frontal pneumatization fills the glabellar area and extends laterally above the orbital notches in slightly more than the medial third of the frontal torus. This would correspond to a lateral extension (noted W here) of 50 mm, which is comparable with the values observed in the more pneumatized *H. erectus s.l.* analyzed here. The sinuses are large antero-posteriorly, as they fill the thick frontal torus, and they propagate slightly into the frontal squama at the center of the marked medial depression of the arched frontal torus.

A look at the Asian continent beyond our sample shows a different view of *H. erectus* sinus morphology. In the Hexian *H. erectus* calvaria, the sinuses are small and form globular cells in the glabellar area (65). In Maba, the sinuses are larger (66) extending into both medial parts of the frontal torus as exposed by small fractures of the bone. We have of course observed some variation among Asian *H. erectus* individuals in the current study (see above).

LB1, the holotype of *H. floresiensis* lacks frontal pneumatization It has been erroneously suggested that cavities visible on low resolution CT data could be the frontal sinuses (e.g., Fig. 2, Brown, 2012:207 (94) or Fig. 7, Falk et al., 2007:8 (95)), but this is not the case, as revealed by our higher resolution dataset (53). A similar case can be made for the Steinheim individual. The frontal sinuses were difficult to visualise, even with a microCT dataset, because of the post-mortem alteration of the internal structure of the fossil (96). However, we are confident in our determination that allows a proper description of the pneumatization. Previous description of huge sinuses invading the frontal squama is erroneous (4), probably in due to misinterpretation of low resolution imaging dataset (see also 6).

Frontal bone pneumatization and phylogenetic implications

We can draw some conclusions from the variation observed in *Pan* and *Gorilla* (9) and among different hominin species. Importantly, some differences are visible between the taxa analyzed. Moreover, these differences can be interpreted through an evolutionary perspective and can be interpreted as primitive or derived states.

What is the expression of frontal pneumatization in the first hominins?

As a group, early hominins, including here *Sahelanthropus*, *Australopithecus* and *Paranthropus*, plot comfortably within the variation observed for *Pan* and *Gorilla* and at some distance from the distribution of *Homo* individuals. This observation is true when sinus size and endocranial size are plotted together but also in our multivariate analysis of sinus dimensions. In fact, early hominins tend to have relatively large sinuses compared to their endocranial size when the general trend among hominins is observed (Fig 1). More generally, variation in absolute sinus volume is quite similar in the different extant genera and hominin species and the ranges of variation for *Pan* and *Gorilla* and hominins considered as a whole overlap. The exceptions are the few hominins with exceptionally large sinuses, Broken Hill, Bodo and Petralona, but also the 12% of the recent *H. sapiens* sample with sinus aplasia and few individual cases of *Homo* fossils with no frontal pneumatization.

We have previously proposed that *P. paniscus*, *P. troglodytes* and *G. gorilla* exhibit the primitive condition for the characteristics of frontal pneumatization, relative to *H. sapiens* (9). *H. sapiens* differs from *Pan*, *Gorilla* and other hominines in its relatively smaller sinuses that are more variable in shape and size. Early hominins with small endocranial sizes appear to follow the trend observed for *Pan* and *Gorilla*. Their sinuses follow the allometric trend linking their size to brain size (Fig. 1) and their sinus shape shows similar characteristics, as highlighted by the multivariate analyses with absolute and relative measurements (Figs. 2 and 3). This observation concerns Toumaï; Sts 5, SK48, StW 573, U.W. 88, SK 48, OH5 and StW 53. Only Sts 71 and StW 505 plot outside of the distribution of *Pan* and *Gorilla*. However, the development of the pneumatization is not complete in Sts 71, and StW 505 does not plot so far from *Pan* and *Gorilla* and does not appear to be an outlier in its morphometric dimensions among early hominins. Our results appear to suggest that the larger space available in the frontal superstructures of *Pan* and *Gorilla* and early hominins gives the frontal sinuses the opportunity to develop in a way that is weakly constrained by other surrounding structures (see also 10).

We were able to study sinus shape on only a few individuals possibility attributed to early *Homo*. Moreover, the analyzed individuals had various taxonomic attribution (StW 53: *H. habilis* or *H. gautengensis*; SK 847 *H. ergaster*, *H. gautengensis, P. robustus*...) and are fragmentary or their sinuses were not visible on the imaging datasets (KNM-ER 1805 and 1813). In this context, early *Homo* will deserve a particular attention for frontal sinus shape in order to clarify their characteristics relatively to early hominins and later *Homo*.

In relation to the unique information collected on most hominin species, what are the implications of the variations in frontal bone pneumatization for phylogenetic interpretations of hominin taxa?

A consequence of this conclusion is that the size and shape of the frontal sinuses are likely uninformative in the context of the discussion on the taxonomic attribution of TM 266-01-060-01, StW 573, U.W. 88 and StW 53. The sample analyzed here is the largest ever studied for early hominins but due to the fragmentary nature of the fossil record, it contains only a few individuals for each species and only one for many of them. In this context, no particular feature was observed in this sample that could discriminate between taxa. Based on the available hominin record, we conclude that frontal pneumatization does not appear to be decisive for the diagnosis of the

following hominin species: *Sahelanthropus tchadensis*, *A. prometheus*, *A. sediba* and *H. gautengensis*.

In contrast to early *Homo*, we observe some differences in the morphometric analyses, both for absolute and relative data, between *H. erectus s.l.*, *H. neanderthalensis*, *H. heidelbergensis* and fossil *H. sapiens*. Our results suggest that frontal pneumatization develops in these later hominins in relation to new and variable constraints related to factors such as the integration between the cranium, brain and frontal sinuses. We therefore consider that the shape of the frontal sinuses shows some characteristics that are a derived condition in later *Homo*, including *H. sapiens*, compared with *Pan*, *Gorilla* and early hominins. These differences may be an indirect consequence of the differences in cranial morphology between taxa, but that does not prevent their potential utility in taxonomic analyses.

We observe a good relationship between the relative development of the sinuses on the left and right side and the pattern of frontal petalia in *H. erectus s.l.* A similar trend was demonstrated for extant *H. sapiens* (9). The condition for the other recent hominins is not clear due to small sample size or incomplete preservation of the sinuses. Nevertheless, a general link between the position of the sinus and the petalial pattern and position of the frontal poles is evident. Further investigation of this relationship may shed light on our understanding of frontal sinus development and also the factors underlying intraspecific variation in frontal sinus morphology.

The variation in frontal sinus morphology within *H. erectus s.l.* is large. In Asia, the Zhoukoudian fossils and the Ngandong and Sambungmacan individuals tend to have small sinuses, and several do not have frontal pneumatization at all. Sinuses are larger in the more ancient Indonesian and African individuals. The Dmanisi sub-sample is a good example of the wide variation in *H. erectus* as variation is already great in this sample of only 5 individuals from the same place and period. *H. erectus s.l.* shares with *H. sapiens* relatively wide variation in the size and shape of the sinuses, including a good proportion of aplasia. Due to the large chronological and geographical extensions of this fossil sample we cannot clarify the origin of this variation. High levels of craniodental variation of the within *H. erectus s.l.* have also been noted by other authors (e.g., 28, 29) and have caused some to argue that the taxon should be divided (30, 31), although many see this level of variation as commensurate with what should be expected in a long-lived, geographically wide-spread primate species (28, 32).

H. naledi is in the centre of the variation observed for *H. erectus s.l.* for both multivariate analyses of absolute and relative data. It falls outside of the distribution of the other fossil hominin groups analyzed.

H. neanderthalensis do not have absolutely or relatively large sinuses compared to other hominins (see also 6, 10, 35) but the shape of the sinuses is particular, and could be related to the specific shape of the frontal torus in this species. Moreover, *Homo neanderthalensis* exhibit a reduced variation in sinus shape and size compared to other fossil hominins. A very homogenous pattern with low variation was also observed for temporal bone pneumatization (39). This is interesting in the context of ancient DNA studies, which have demonstrated low levels of genetic diversity and frequency of inbreeding within *Homo neanderthalensis* (38, 40). When included in multivariate analyses, they plot with other hominins, at the intersection of the distributions of *H. sapiens*, *H. erectus* and *H. heidelbergensis*. *H. sapiens* individuals, considering both the fossil and recent samples, show wide variation in the shape and size of the frontal pneumatization, as has previously been shown (6, 9, 97).

Among *H. antecessor*, *H. rhodesiensis* and *H. heidelbergensis s.l.*, high levels of variation are visible, particularly because of the huge pneumatization of a few key individuals: Bodo, Broken Hill and Petralona. The size of the pneumatization of these three individuals distinguishes them from all other samples. In terms of relative dimensions, the three fossils plot at the intersection of early hominins and *H. erectus* in relation with a relatively large antero-posterior size of sinuses compared to other hominin species. As a result, they are unique in terms of size and shape of their sinuses and this observation certainly deserves some further attention in future work (see also 4, 6, 10, 11).

Can we identify specificities in frontal sinus morphology among "Middle Pleistocene hominins"?

In contrast to Bodo, Petralona and Broken Hill 1, the other fossils grouped in the Middle Pleistocene sample have a particular position in the multivariate analyses compared to the other hominin samples. Quantifying frontal sinus shape is complicated, however, due to high levels of variation in size, the multilobed shape of many Middle Pleistocene hominin frontal sinuses, and the variable presence of either one or two separate sinuses in the frontal. The individuals named here *H. heidelbergensis* (8), with less massive frontal pneumatization than Bodo, Petralona and Broken Hill 1 mostly plot within or very close to the range of variation for *H. neanderthalensis* for absolute dimensions.

Zuttiyeh, for example, fits well among *H. heidelbergensis*. Concerning the discussion about its taxonomic position (33, 34), the contribution of frontal bone pneumatization is that this individual shows closer affinities for this anatomical area with *H. neanderthalensis* than with *H. erectus s.l.* The exceptions to this trend of *H. heidelbergensis* grouping with *Homo neanderthalensis* are SHS 13 and SHS 17 who have smaller sinuses, and Steinheim which plots with *H. erectus* but whose sinus shape and extension was certainly modified by taphonomic alteration. When relative dimensions are considered, *H. heidelbergensis* are slightly more outside the range of *H. neanderthalensis*, but the latter are particularly homogeneous and most of the *H. heidelbergensis* sample are very close to this distribution. This general observation agrees with the generally accepted evolutionary trend suspected between European *H. heidelbergensis* and their successors, *H. neanderthalensis*.

Can the development of the frontal sinus be related to biomechanical demands?

One of the most frequently discussed hypotheses of sinus function and development is that they serve to disperse masticatory strains (see Introduction). Various studies have recently addressed this topic through different approaches. Virtual assessment of the effect of variation in presence, size and shape of the frontal sinuses in Broken Hill 1 subjected to masticatory strains has been attempted (42). This paper shows that sinus modification does not have an impact on the magnitudes and direction of strain, or how the skull is affected by those strains (42). We also studied the external morphology of the face, together with the variability of frontal pneumatization, in a sample of fossil *H. sapiens* from Afalou Bou Rhummel (Algeria) and Taforalt (Morocco). Those populations underwent extraction of upper incisors (41). The only noticeable influence of the modification is limited to the premaxilla whereas the variation in frontal sinus was not affected. We deduced that biomechanical stress has limited influence on the development of the face and

pneumatization in this sample. The example of the work of Godinho et al. (2018) (42) shows that finite element analysis is useful to simulate loading scenarios and the resulting straining of the skull areas (98). However, we should keep in mind that the employed virtual constraints are not real and that anatomical structures are subject too multiple constraints.

Our results here illustrate various patterns of frontal sinus variation in *Pan*, *Gorilla* and hominin species, given between taxa likely undergoing relatively similar masticatory strain regimes, such as *H. erectus sensu lato* and *H. rhodesiensis*. Based on these observations, it is very unlikely that sinus size and shape are driven by masticatory strains in hominins.

Some cases of metopic suture in the hominin record and their influence on frontal sinuses

In our sample, we observed two hominin fossils with uncommon characteristics in their frontal pneumatization. In D2700 there is no true frontal sinus, whereas they are quite large in all the other fossils from Dmanisi. This pattern of variation is not unexpected in relation with the variation observed in *H. erectus s.l.* However, other samples for this species show more homogeneous variation, the sinus are small in all individuals of from Ngandong or Zhoukoudian for example, and seem to be larger in the older sample of Javanese fossils. In Hofmeyr, the sinuses are small and have an asymmetric, irregular and unexpected shape (see also 99). Again, it is not improbable to observe small sinuses in a *H. sapiens* fossil in relation with the variation seen in this species. However, their shape is uncommon. These two individuals exhibit a remnant of the metopic suture in the anterior part of the glabella. The correlation between sinus size and shape and the persistence of the metopic suture is complex to evaluate. No longitudinal study exists yet on this topic; however, it has been shown in *H. sapiens* that absent and small sinuses were considerably more frequent in skulls with a persistent metopic suture, compared to the control sample (100). Our results suggest this observation may also apply to Upper Palaeolithic *H. sapiens* and *H. erectus s.l.*

Does brain shape influence sinus shape?

We were able to compare the position and extension of the frontal poles and those of the frontal sinuses in only three early hominins, Sts 5, StW 505 (*A. africanus*) and U.W. 88 (*A. sediba*). These fossils show a left frontal petalia and a larger frontal sinus on the contralateral side. This pattern is clear from the quantified metric variables (Tables S3) but also from the visual inspection of the features (Fig. S2-S70). This relationship is striking, but difficult to interpret. A larger sample that preserves both the frontal lobes and the frontal pneumatization without distortion is necessary to explore the validity of a correlation between the two and explore its origin in detail.
More information was obtained for later hominin species with larger samples. In sum, in contrast to the potential pattern in early *Homo*, we observe that a more anterior and lateral extension of the right frontal lobe of the brain compared to the contralateral side is a general pattern that becomes consistent in *H. erectus*, *H. heidelbergensis* and *H. neanderthalensis*. This asymmetry is a well-known feature of *H. sapiens* in the neuroscientific literature (e.g., 48-50), and is also generally recognized among fossil hominins (47, 101). We also see some general tendencies in the bilateral expression of the sinuses in these taxa. Some limitations make it difficult to draw a general picture of this trend, however. Indeed, bilateral variation in the traits analyzed is small in size and incomplete anatomical preservation or alteration of the original shape may alter the observation of such details, particularly for the more ancient fossils. A specific study should be developed on this topic in the future to look more closely at those features and to clarify the observed link in some

early hominins between frontal lobe petalias and sinus preferential development on one side. The great variation in the development of the frontal sinuses, despite the large space available in the superstructures of the frontal bone in all the hominins species analyzed except *H. sapiens*, illustrates that complex interactions occur between the different anatomical areas of the anterior skull. However, our observations here and the results that we have previously obtained from samples of recent *H. sapiens* (9) offer some information about frontal sinus shape and the influence of the underlying frontal lobes. We can speculate that covariation existed between the size and shape of the frontal sinuses on both sides and the expression of the underlying development of the frontal lobes, since at least *H. erectus* and that it continued among subsequent hominin species.

Our results on large and diverse samples of *H. sapiens* show that the dimensions of the frontal sinuses are not spatially autocorrelated, but they do differ significantly between geographic regions. Moreover, no direct link is observed between geographic origin and the size and shape of the sinuses, i.e. individuals from colder climate are not characterized by larger/smaller frontal sinuses than populations from warmer areas. If some differences exist between the analyzed sample, they are not related to climate. We propose then that climate does not seem to directly explain the development of frontal sinuses in our species. It is nevertheless likely that sinus shape and variation in living populations around the world may reflect some aspects of the recent history of our species including migrations, genetic drift, local adaptations as shown by different anatomical areas of the skull (e.g., 102) and possibly by the brain. Whether these factors act directly on the sinuses themselves, or indirectly via their effects on craniofacial morphology remains to be determined.

Are the sinuses of *H. neanderthalensis* an adaptation to cold climate?

A very common explanation for the presence of frontal sinuses is that large sinuses serve the function of conditioning inspired air in cold environments. This hypothesis stemmed from suggestions about large *Homo neanderthalensis* sinuses and an assumed cold-adapted niche for this taxon (e.g., 17, 44, 45). Based on our results (Table S3) and from multiple strands of evidence in the literature (6, 10, 35), we may consider it now proven that *H. neanderthalensis* are not hyperpneumatized compared to *H. sapiens*, *H. erectus* or other hominin samples in terms of absolute or relative volume of pneumatization. It has been shown, in fact, that within recent *H. sapiens*, frontal sinuses tend to be smaller in cold environments (this study and 9, 103, 104). Our results do not support a link between small sinus size and cold adaptation in Neandertals any more than they do between hyperpneumatization and cold adaptation. *H. neanderthalensis* relative frontal sinus volumes are neither particularly large nor small compared to those of other hominins. *Homo neanderthalensis* do show, however, reduced intraspecific variation in sinus dimensions, particularly when compared to *H. erectus* or *H. sapiens*, and also homogenous shape likely related to their distinctive frontal torus morphology. There is no clear support for a functional or climatic origin of *Homo neanderthalensis* pneumatization when all the detailed evidence presented above and the variation among hominins are considered. We propose that this hypothesis that the sinuses of *H. neanderthalensis* are due to cold climate should be discounted, unless it can be clearly demonstrated. Finally, we suggest that the low inter-individual variation and particular shape of the frontal sinuses as described here may be considered an apomorphy contributing to the diagnosis of the species *H. neanderthalensis*.

We conclude that the primitive condition of the frontal sinus in *Pan*, *Gorilla* and hominins is seen in *Pan*, *Gorilla* and early hominins, where considerable space available in the frontal superstructures gives the frontal sinuses the opportunity to develop in a way that is weakly constrained by other surrounding structures. In later hominins, new and variable constraints related to the integration between the cranium, brain and frontal sinuses have some influence on the frontal sinuses resulting in limitations on their opportunistic expansion into the superstructures. Even if the sinuses are large in some individuals, e.g. Broken Hill, Petralona and Bodo but also individuals for other *Homo* species, we have detected that their shape, including bilateral variation, was influenced by the surrounding structures. These aspects will also have to be explored in detail while considering the relative position of the orbits, the frontal bone and the frontal lobes (e.g., 105, 106). This is because the morphological integration between these elements could affect the size and shape of the frontal sinuses. Differences are also observed between *Homo* species and these have some implications for phylogenetic discussions, as discussed in this paper. Concerning the correlates of the expression of the frontal sinuses, our results support the assertion that sinus size and shape are not driven by adaptation to masticatory strains in hominins, nor due to climatic adaptation. Interestingly, frontal sinus morphology may be of some interest to discussions of population history in *H. sapiens*. This paper opens up new perspectives for the study of frontal sinuses. We propose a methodology for anatomical description and quantification of the frontal sinuses as well as a global comparative morphometric and anatomic framework for nearly all the existing hominin species. We hope that this work will encourage authors of future descriptions of key fossil hominin skulls to provide detailed information about the morphology and dimensions of the frontal sinuses, for the benefit of the whole paleoanthropological community. This would not prevent researchers from doing additional comparative analyses of their individuals but ameliorates our basic knowledge of the anatomy of the hominin fossil record.

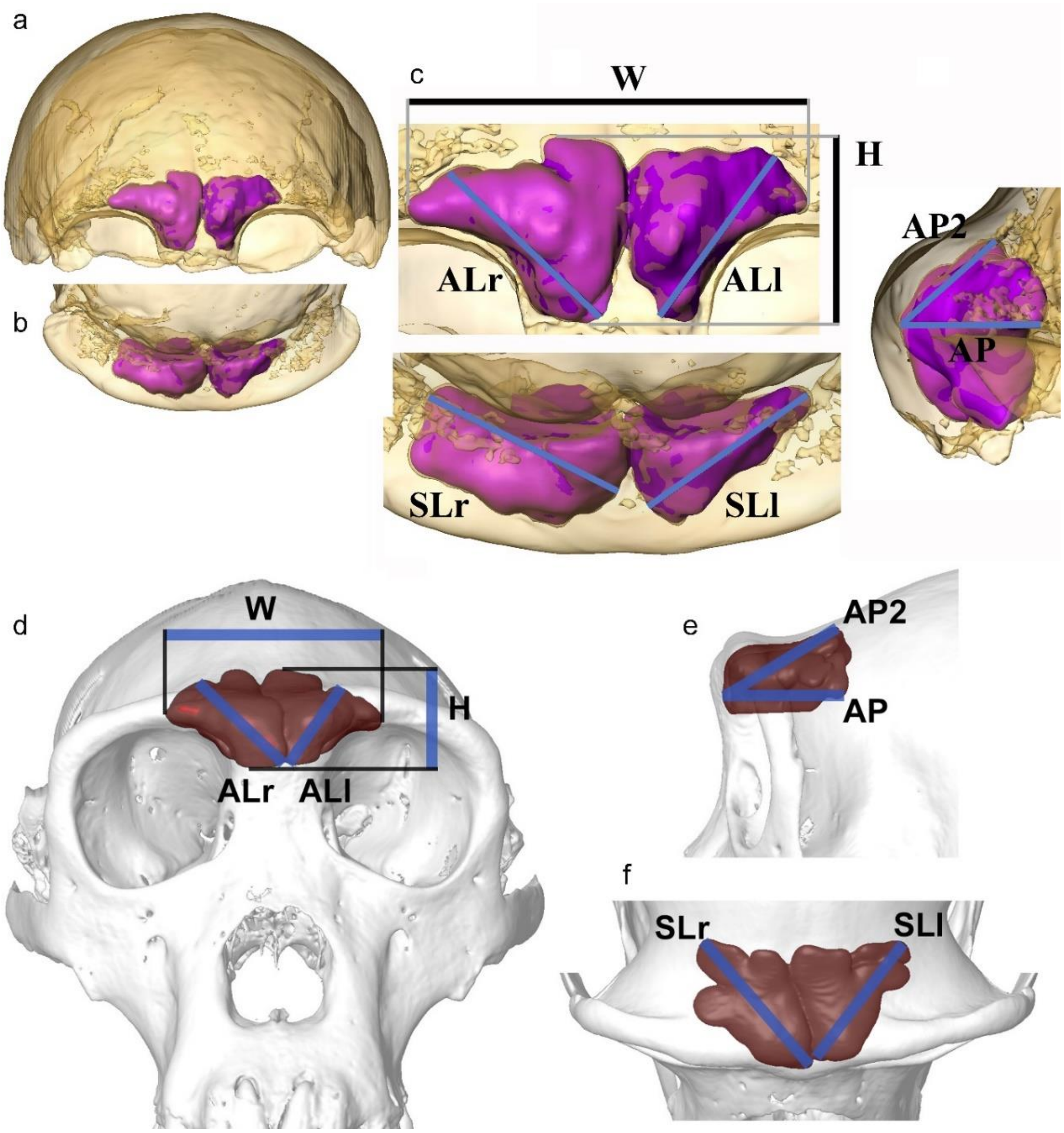

Figure S1. Visualization and quantification of the frontal sinuses. The skull of the type specimen of *Homo neanderthalensis*, Feldhofer 1, in anterior (a), superior (b) and detailed views (c) and the skull of one specimen of *Pan troglodytes* in anterior (d), lateral (e) and superior views (f) (modified from 9). Bone is rendered transparent and sinuses are shown as a virtual solid in color. Dimensions of the frontal sinuses are measured as 2D projections in different orientations and are shown as follows (c and d): maximal lateral extension (W), maximal height (H), maximal length of the left and right frontal sinuses (Anterior Length: ALl and ALr) measured from the most medial and inferior point of the sinus to the more distant point of the extension of the sinus vertically and laterally measured in anterior view; maximal medio-lateral extension of the left and right sinus (Superior Length: SLl and SLr) measured in superior view (c and f); length from the most anteriorly protruding point of the sinus to the most posterior point in an horizontal direction (AP) and length from the most anterior point to the maximal supero-posterior extension of the sinuses (AP2) measured in left lateral view (c and e).

Supplementary figures showing the position and extension of the frontal pneumatization in anterior, lateral and superior views in all the analyzed fossil hominins, for scale refer to the dimensions of the frontal sinuses in Table S2

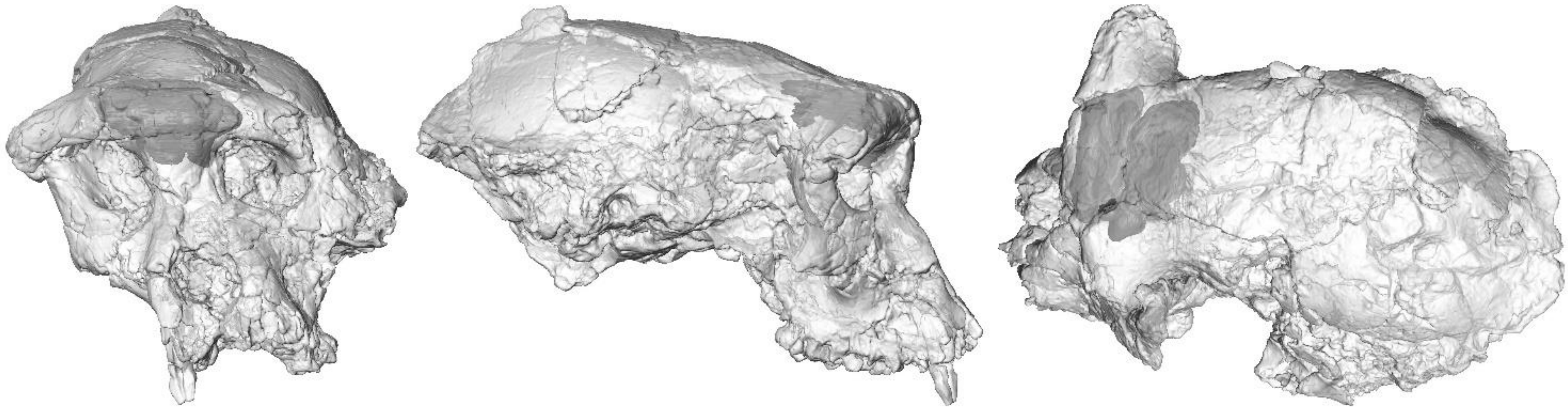

Fig. S2. TM 266-01-060-1, holotype of *Sahelanthropus tchadensis*

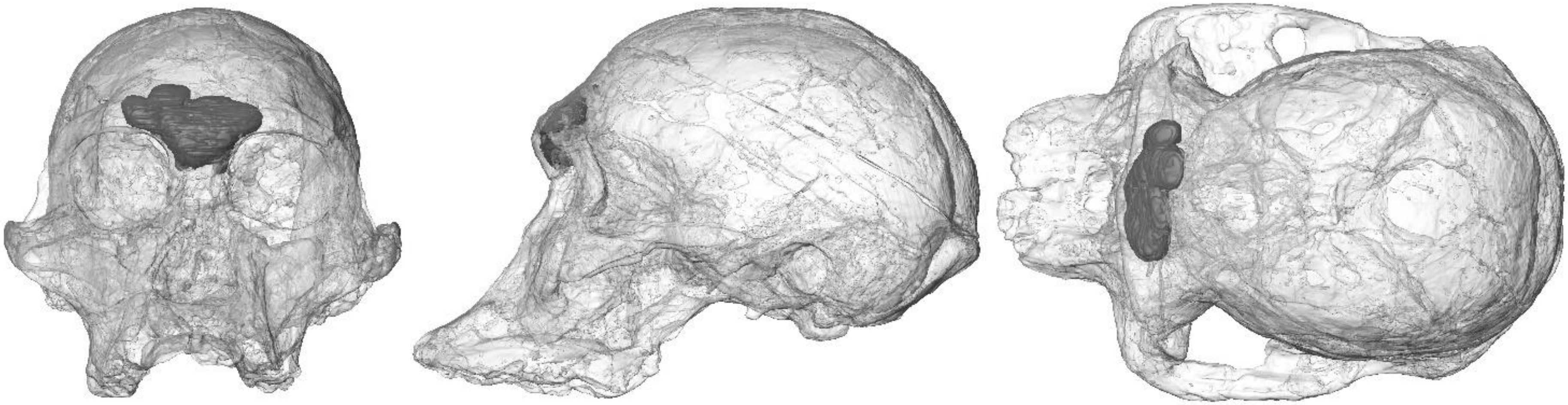

Fig S3. Sts 5, *Australopithecus africanus*

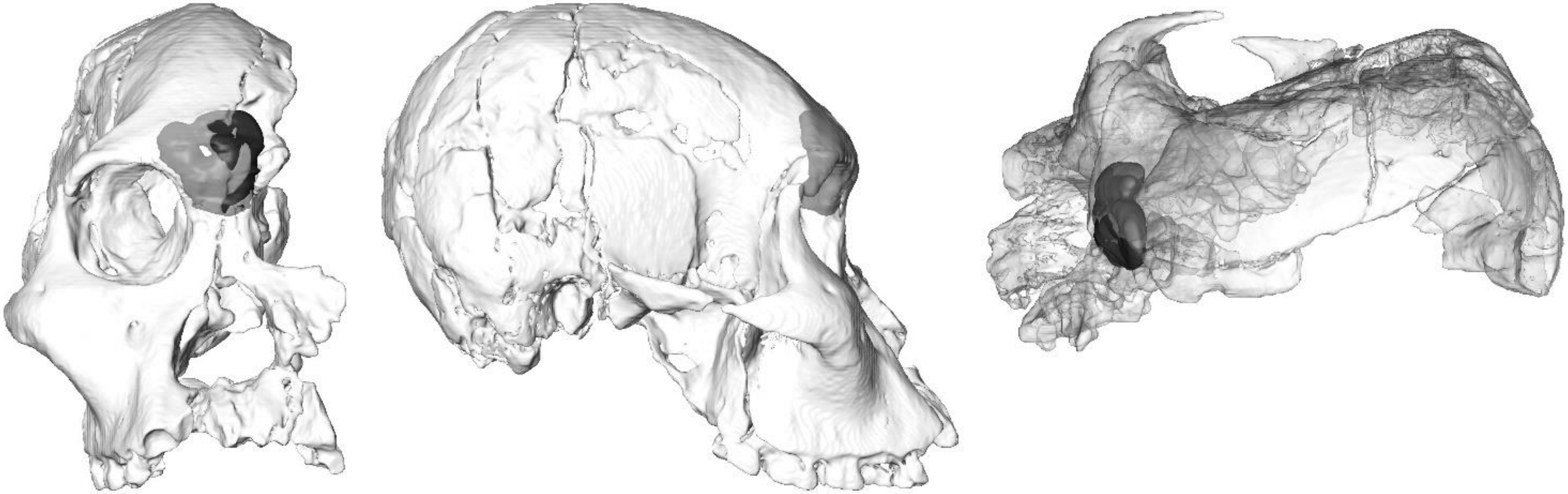

Fig S4. Sts 71, *Australopithecus africanus*

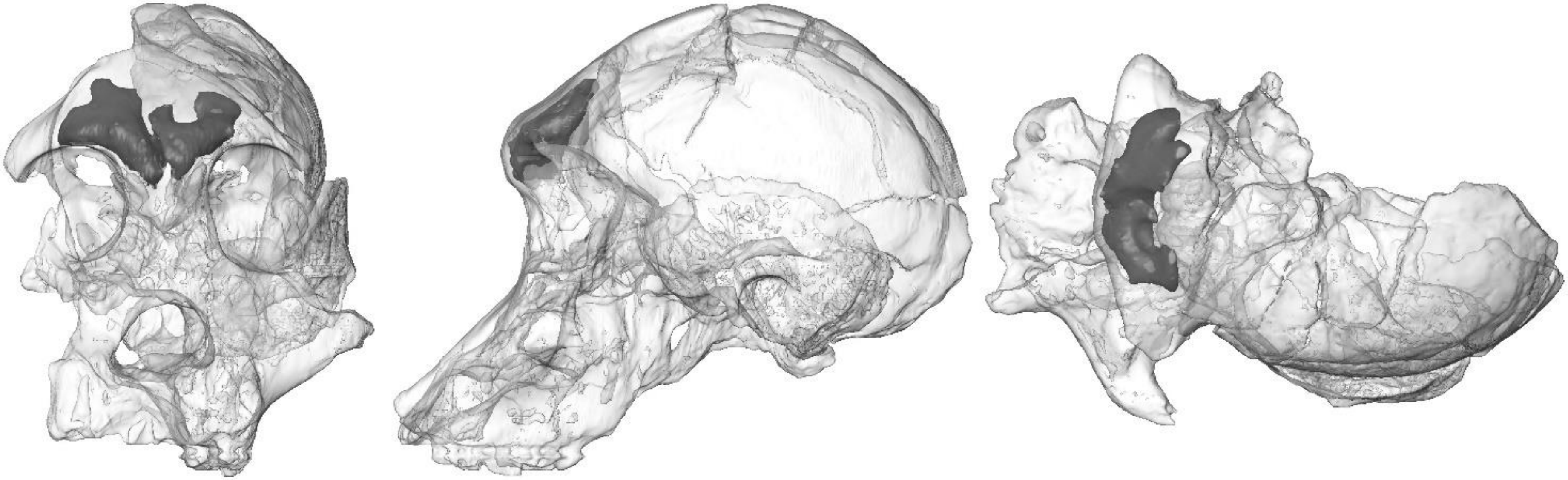

Fig S5. StW 505, *Australopithecus africanus*

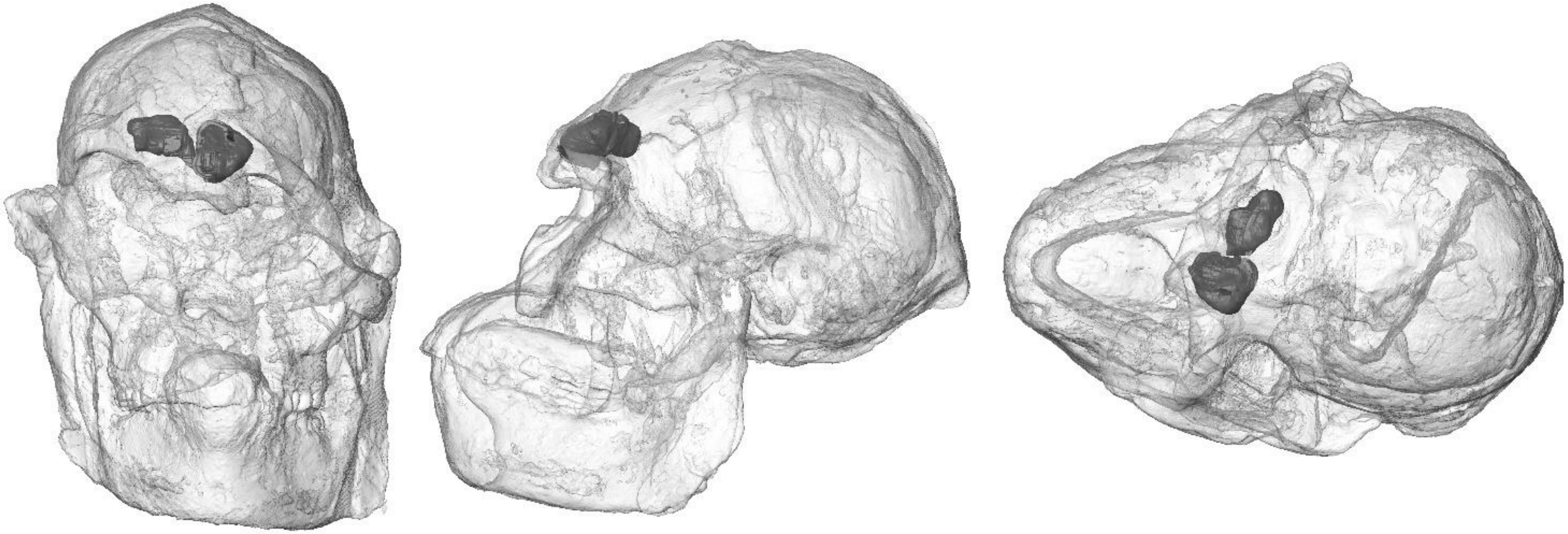

Fig. S6. StW 573, *Australopithecus prometheus*

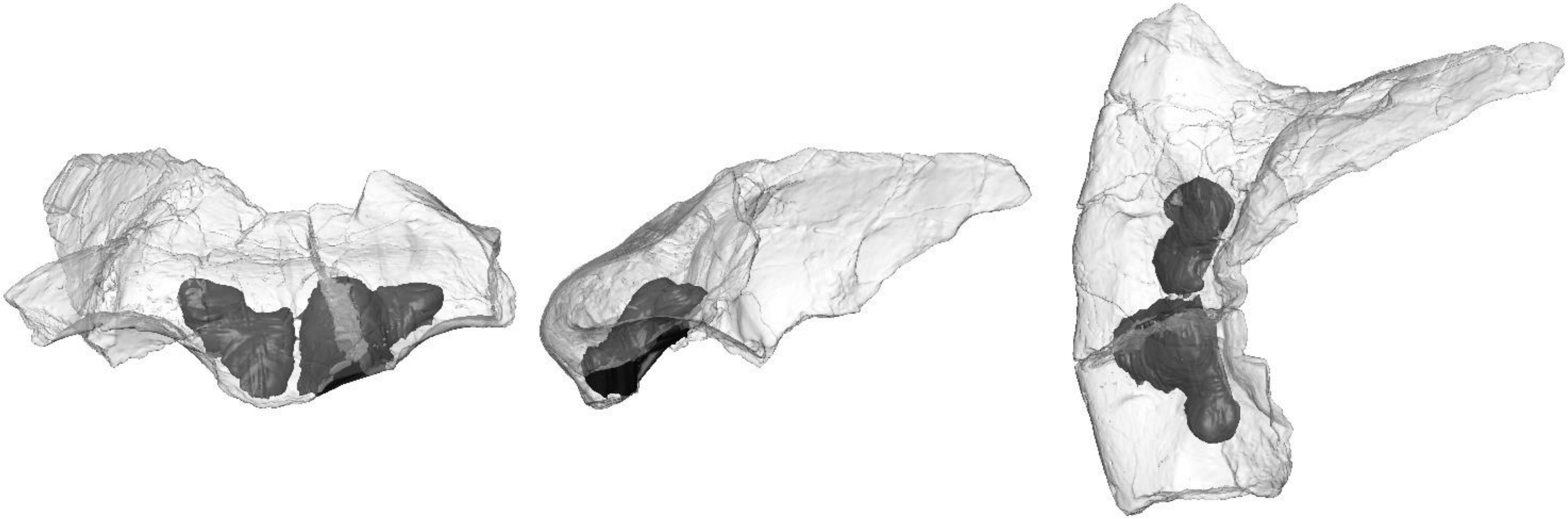

Fig. S7. BOU-VP-12/130, holotype of *Australopithecus garhi*

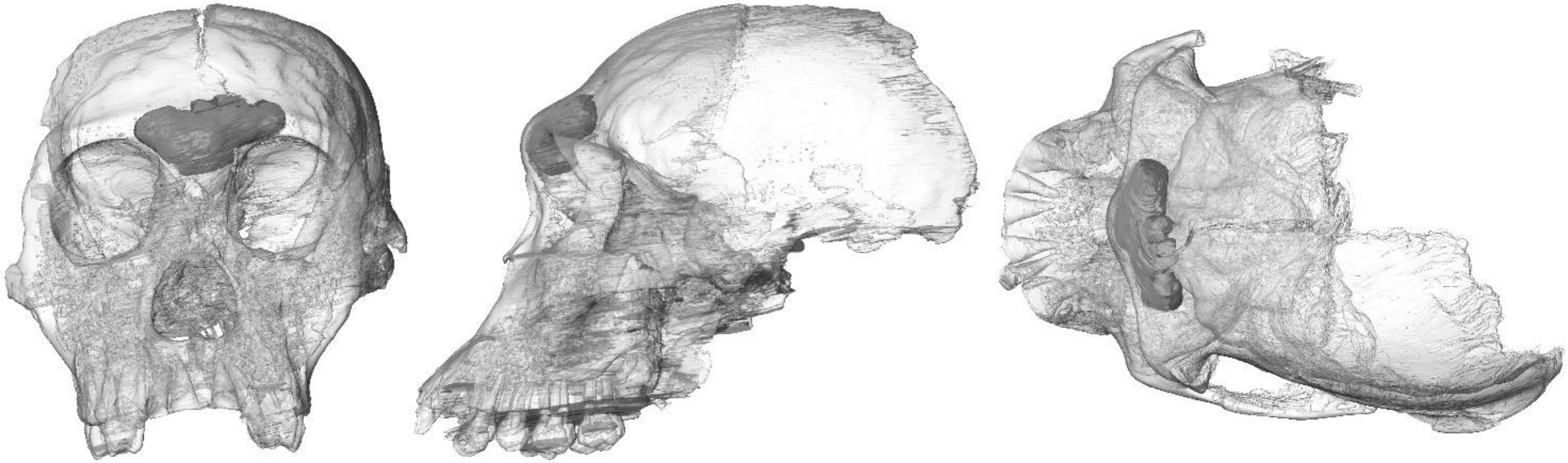

Fig. S8. U.W. 88-50 (MH1), holotype of *Australopithecus sediba*

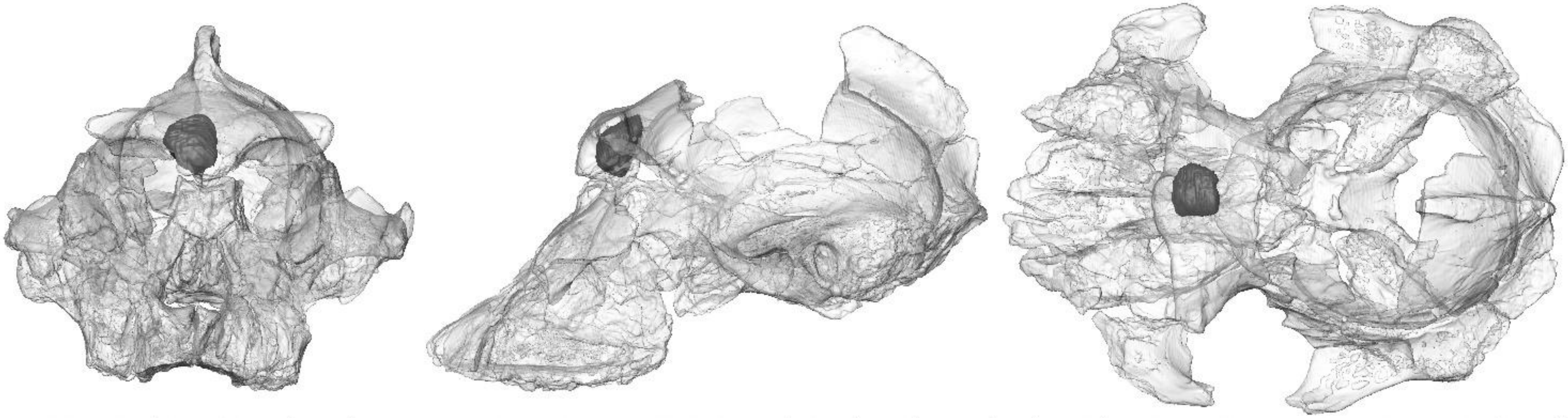

Fig. S9. KNM-WT 17000, *Paranthropus aethiopicus*

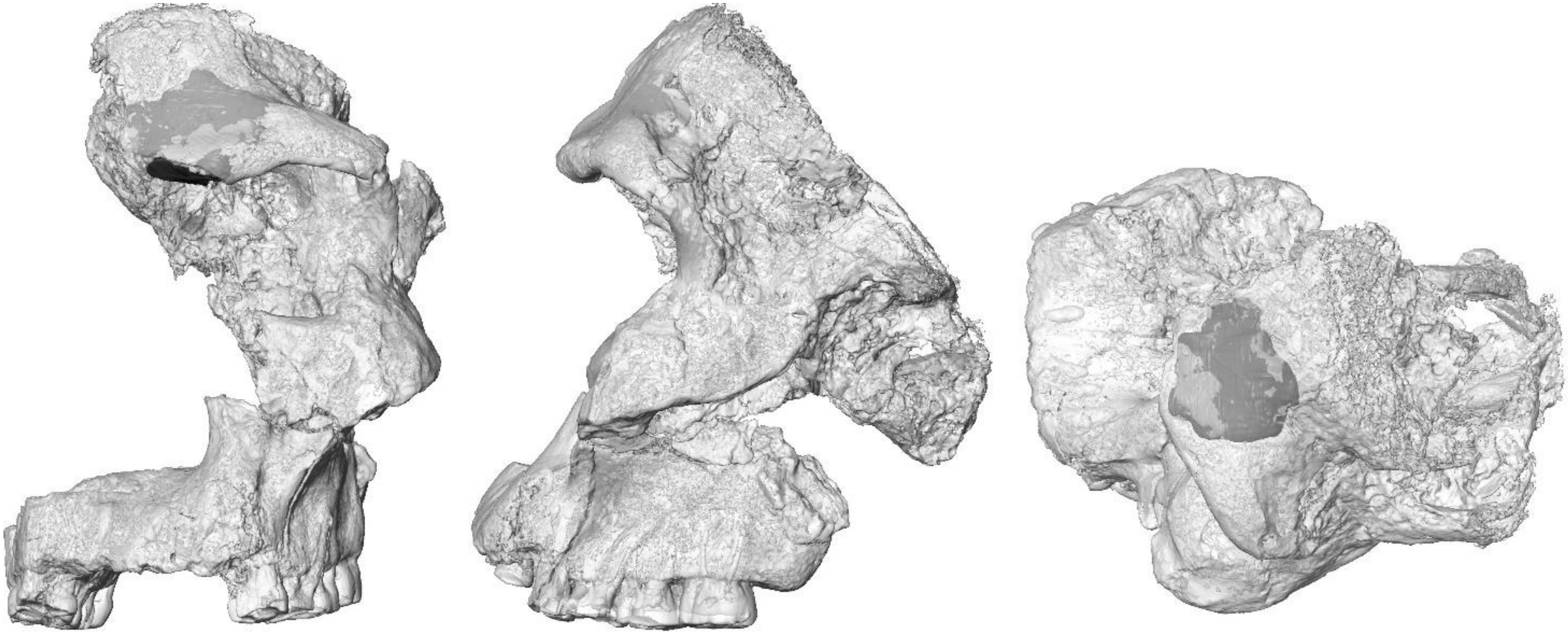

Fig. S10. SK 46, *Paranthropus robustus*

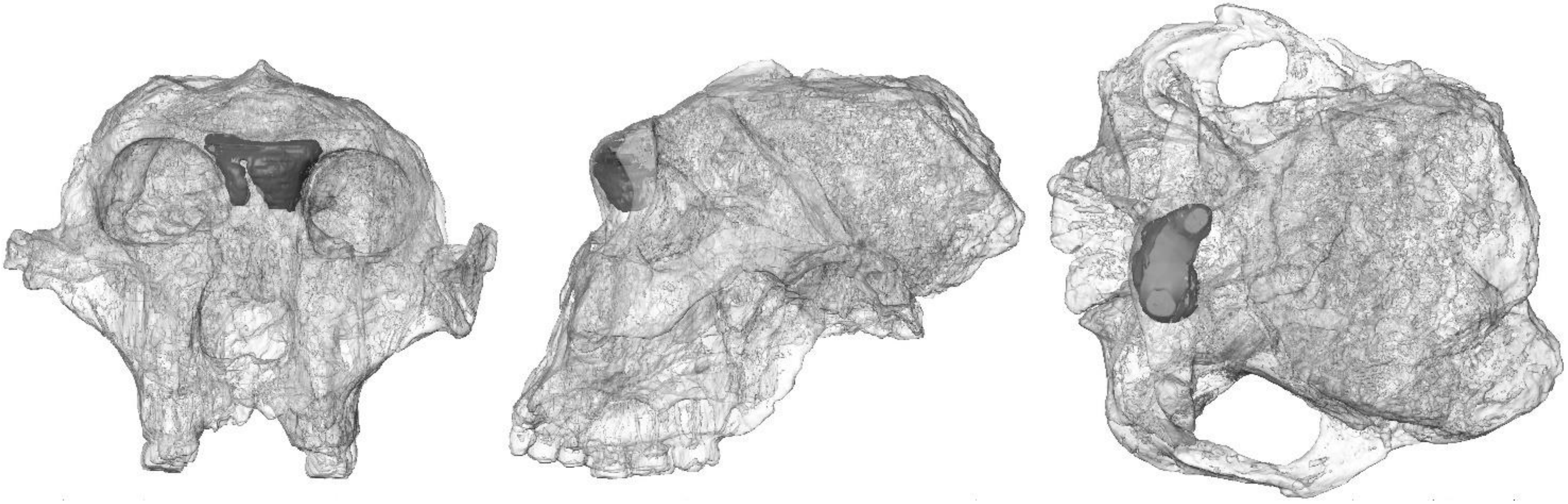

Fig. S11. SK 48, *Paranthropus robustus*

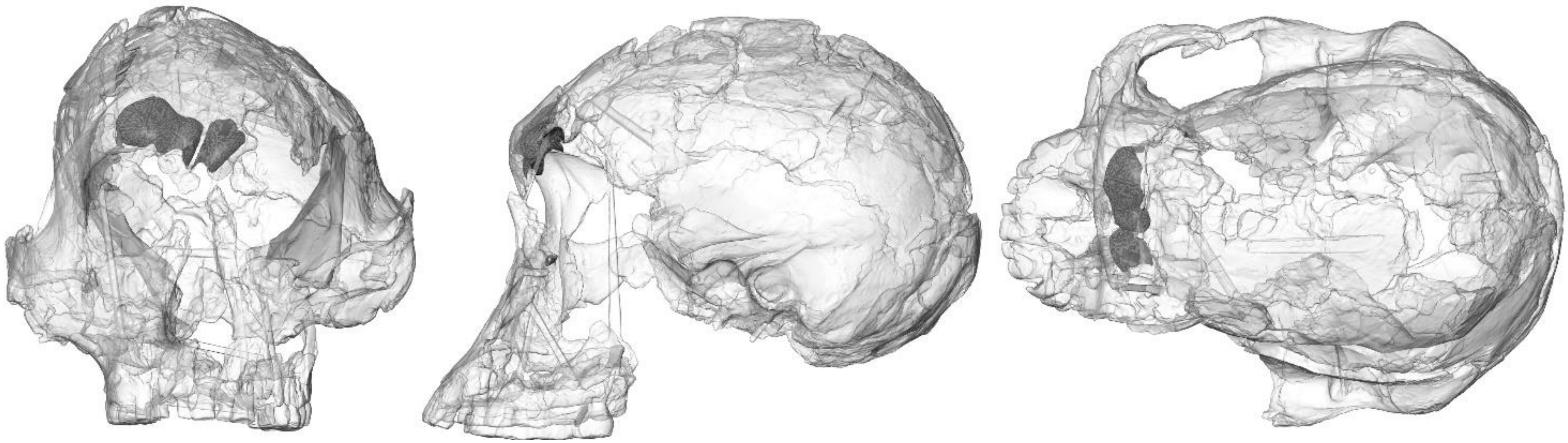

Fig. S12. DNH 7, *Paranthropus robustus*

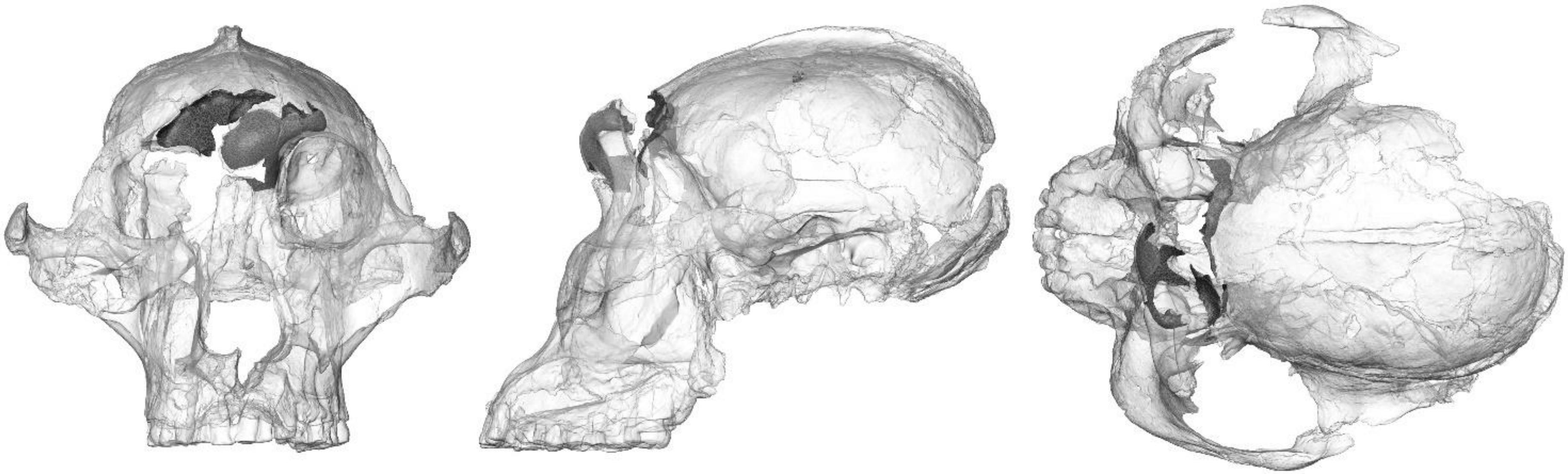

Fig. S13. DNH 155, *Paranthropus robustus*

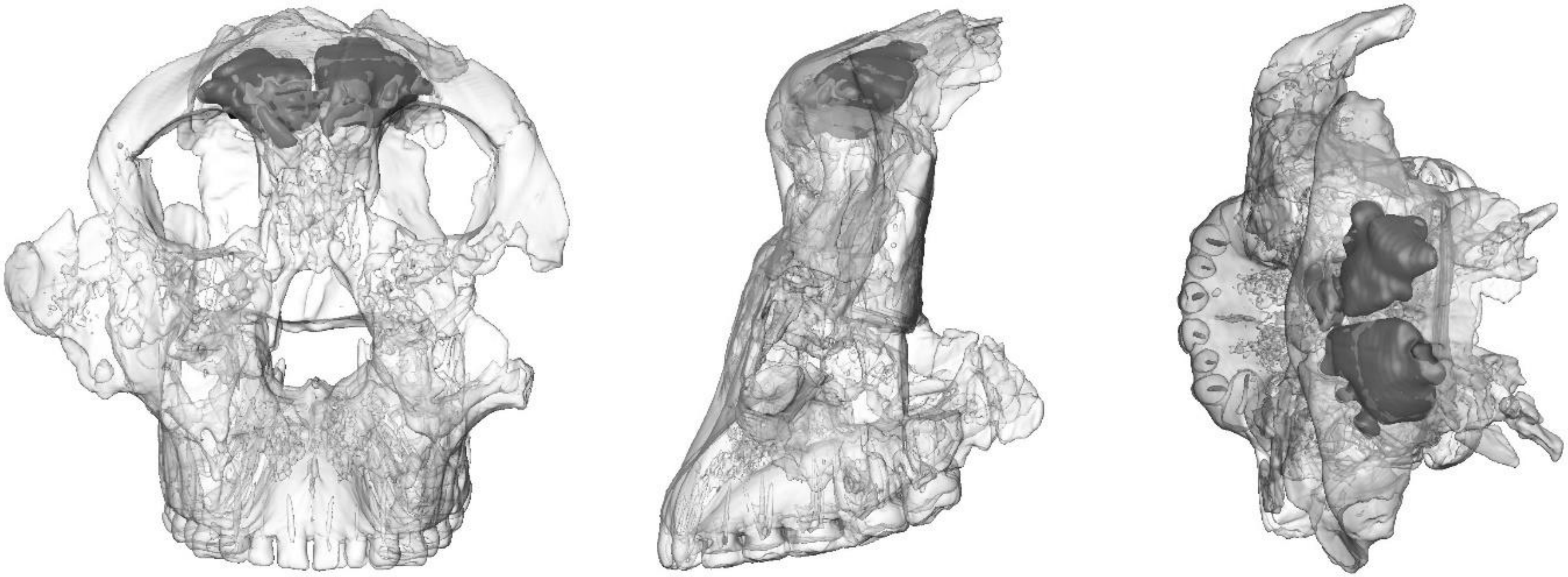

Fig. S14. OH 5, holotype of *Paranthropus boisei*

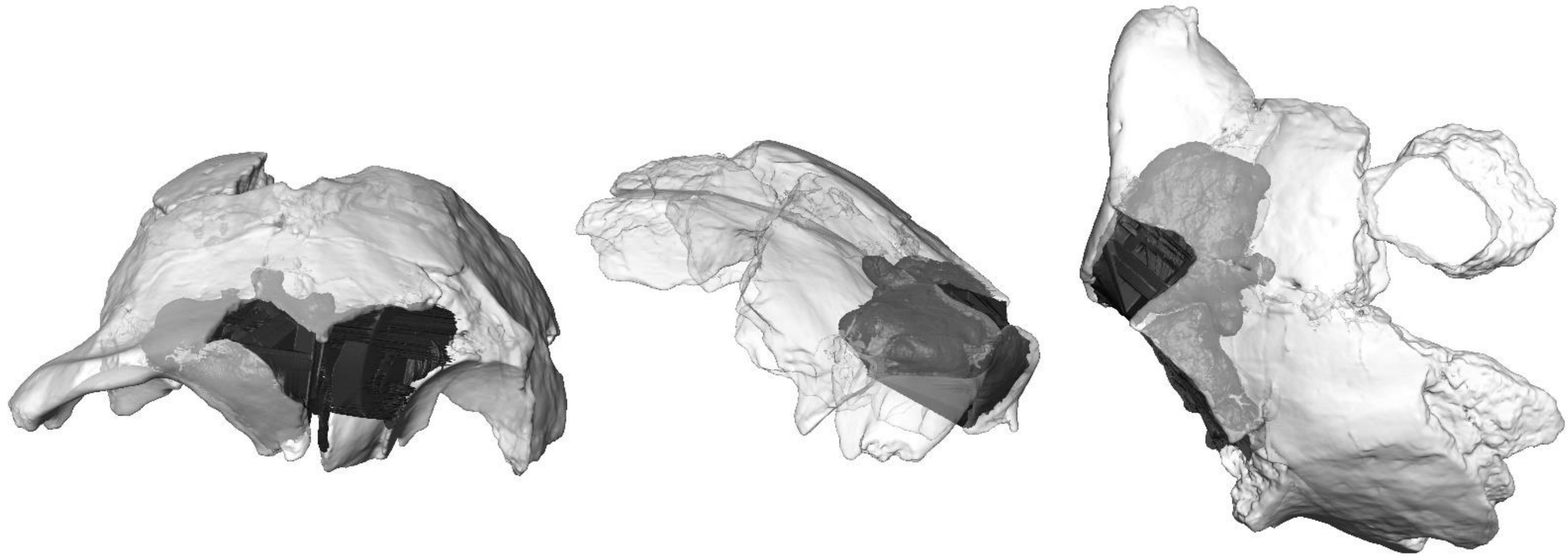

Fig. S15. StW 53, holotype of *Homo gautengensis*

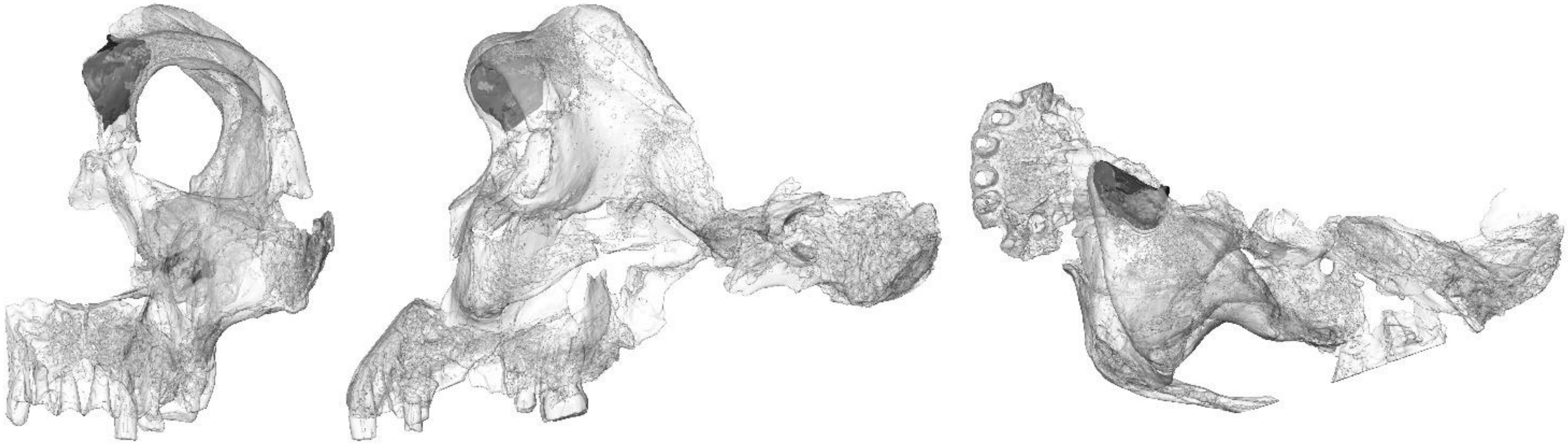

Fig. S16. SK 847, *Homo habilis*

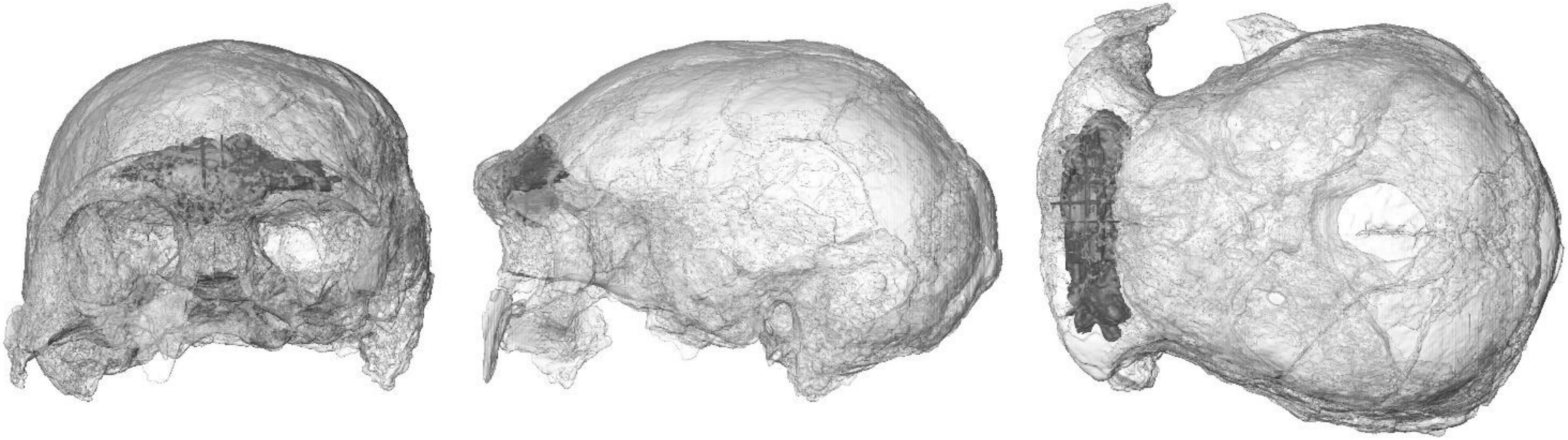

Fig. S17. KNM-ER 3883, *Homo ergaster/Homo erectus*

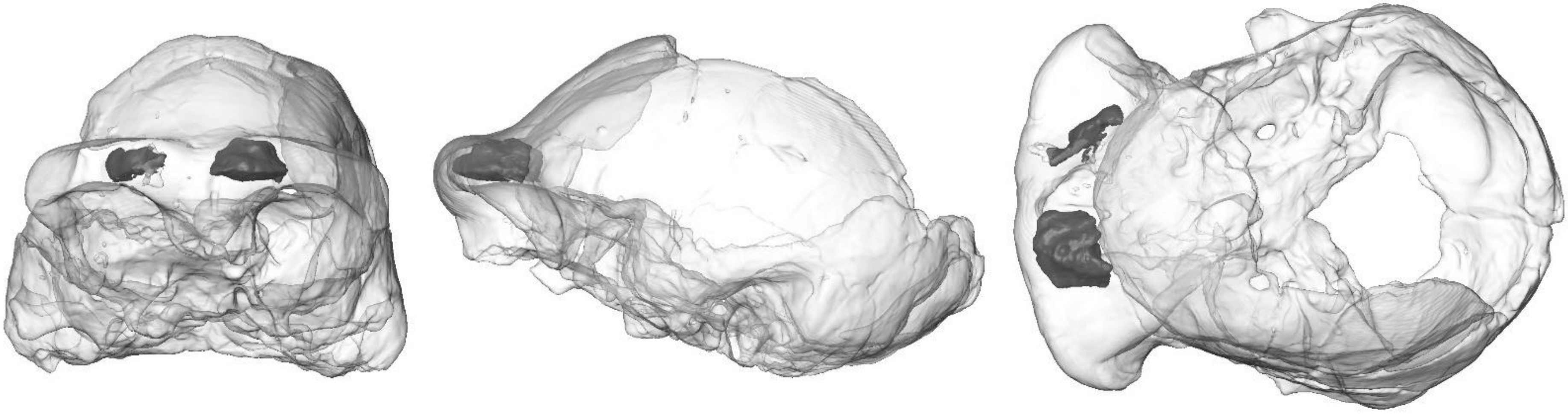

Fig. S18. OH9, *Homo ergaster/Homo erectus*

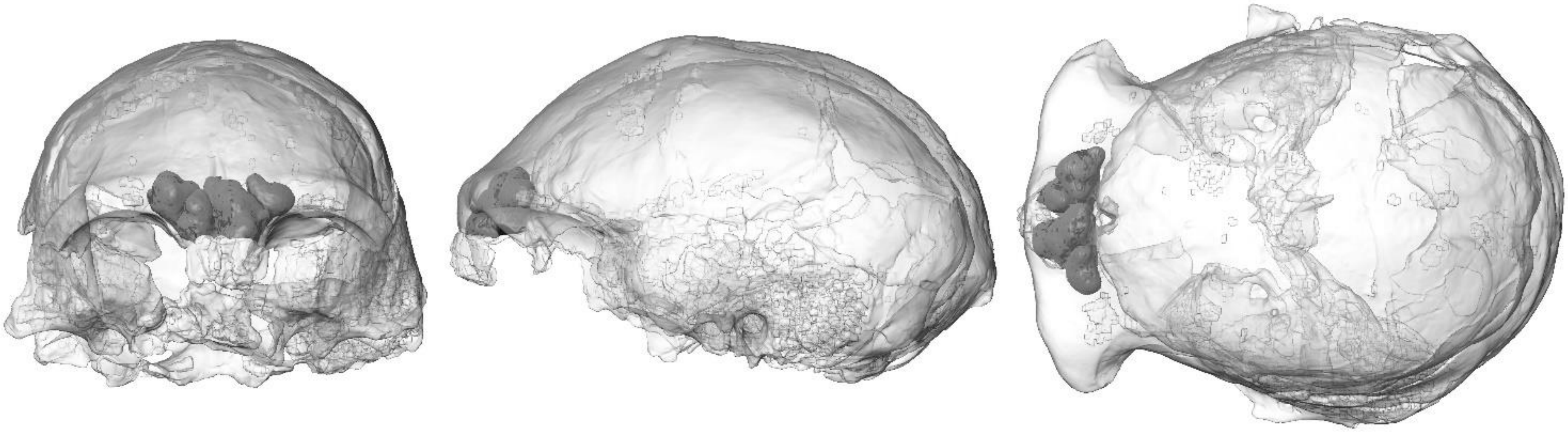

Fig. S19. D2280, *Homo georgicus/Homo erectus*

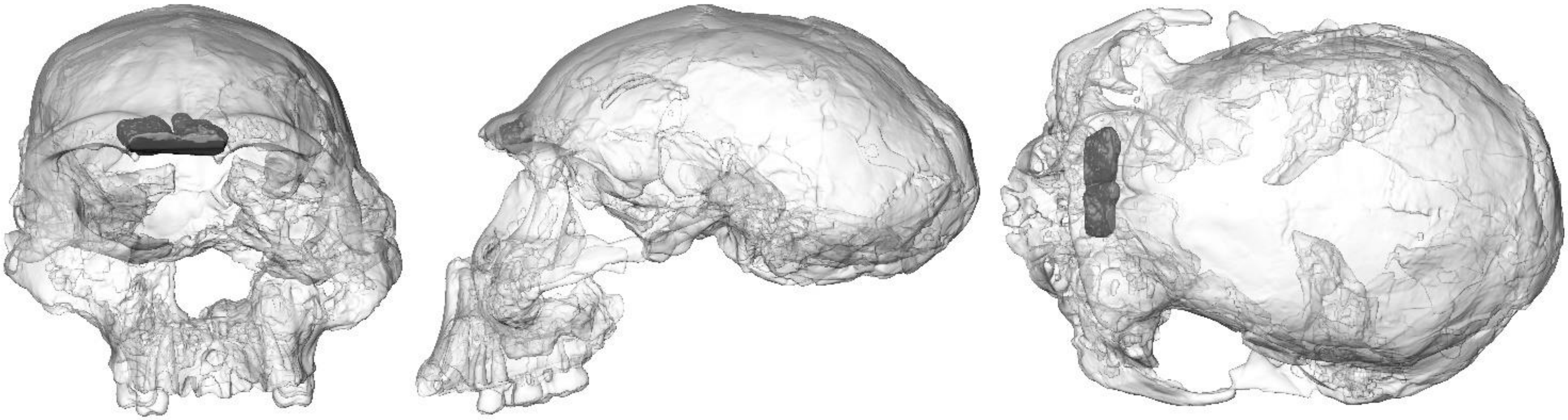

Fig. S20. D2282, *Homo georgicus/Homo erectus*

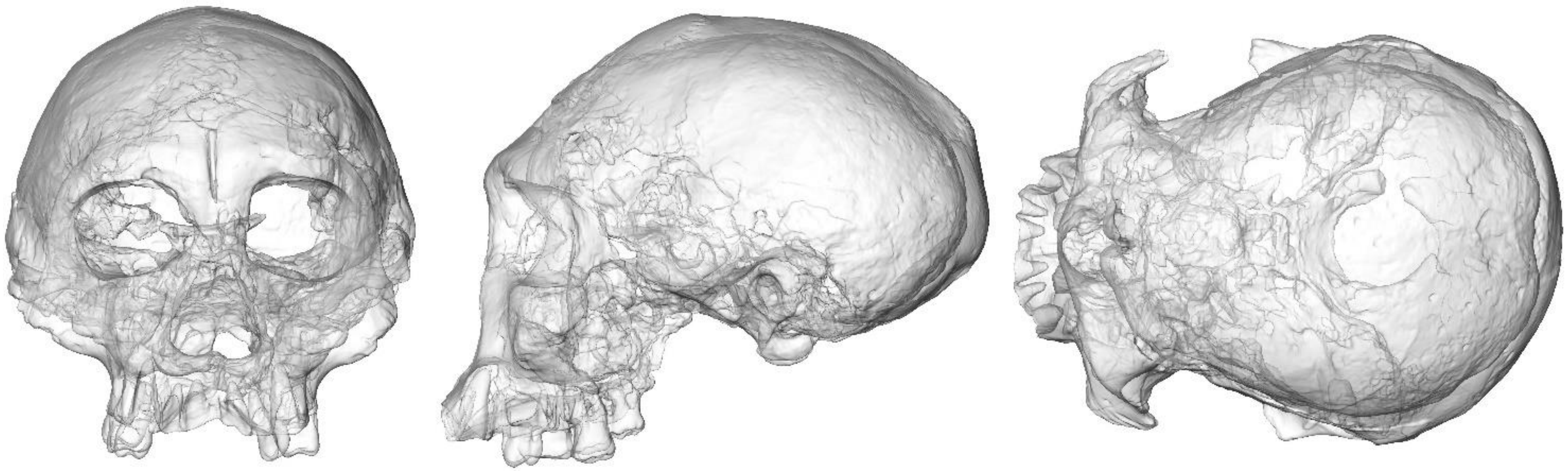

Fig. S21. D2700, *Homo georgicus/Homo erectus*

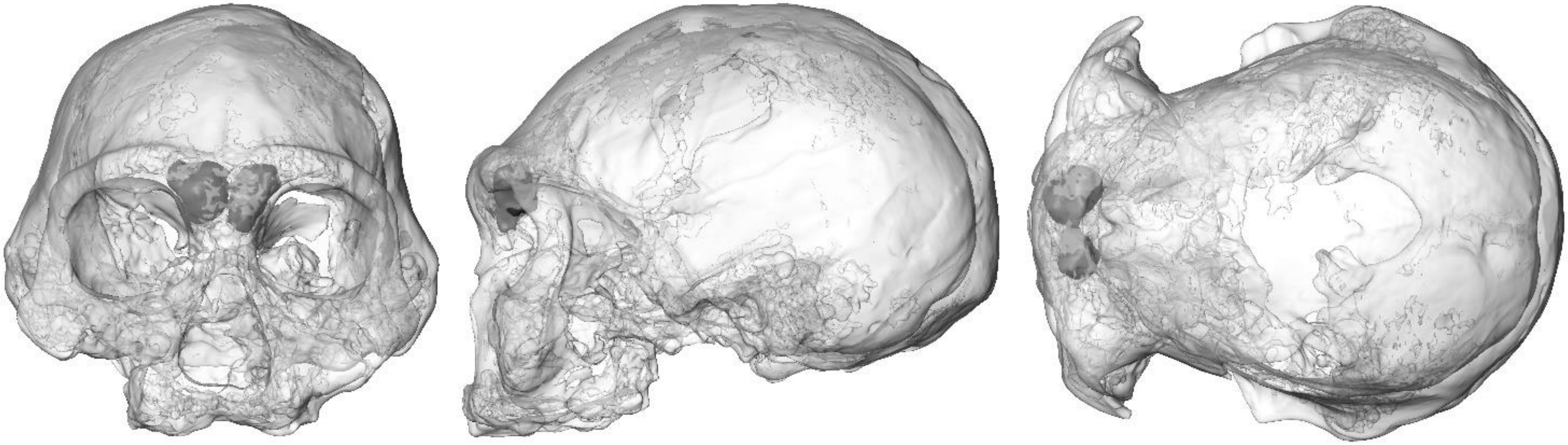

Fig. S22. D3444, *Homo georgicus/Homo erectus*

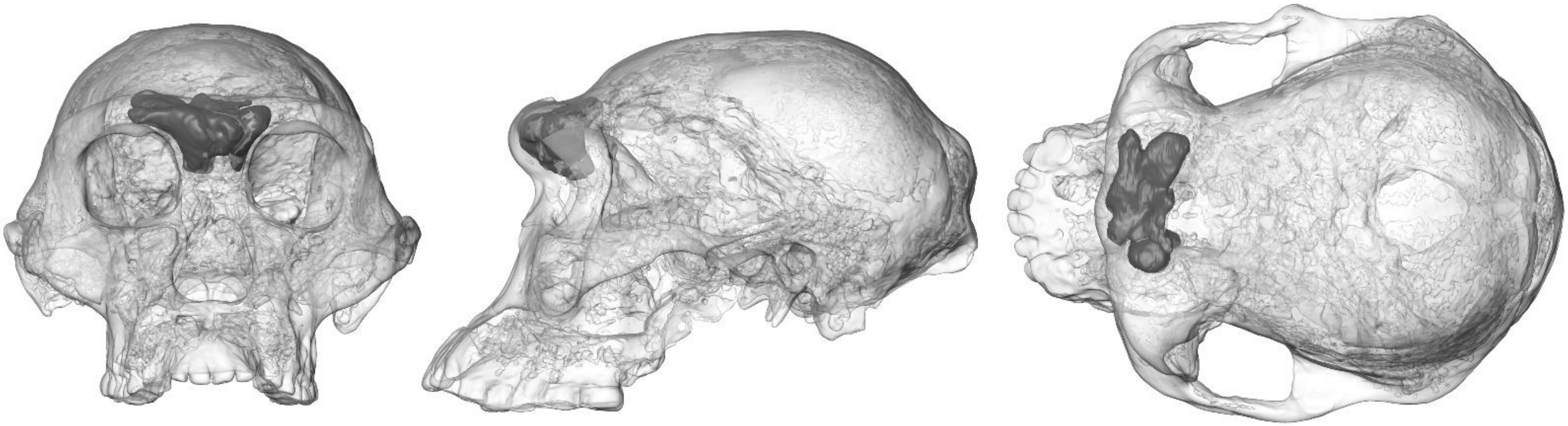

Fig. S23. D4500, *Homo georgicus/Homo erectus*

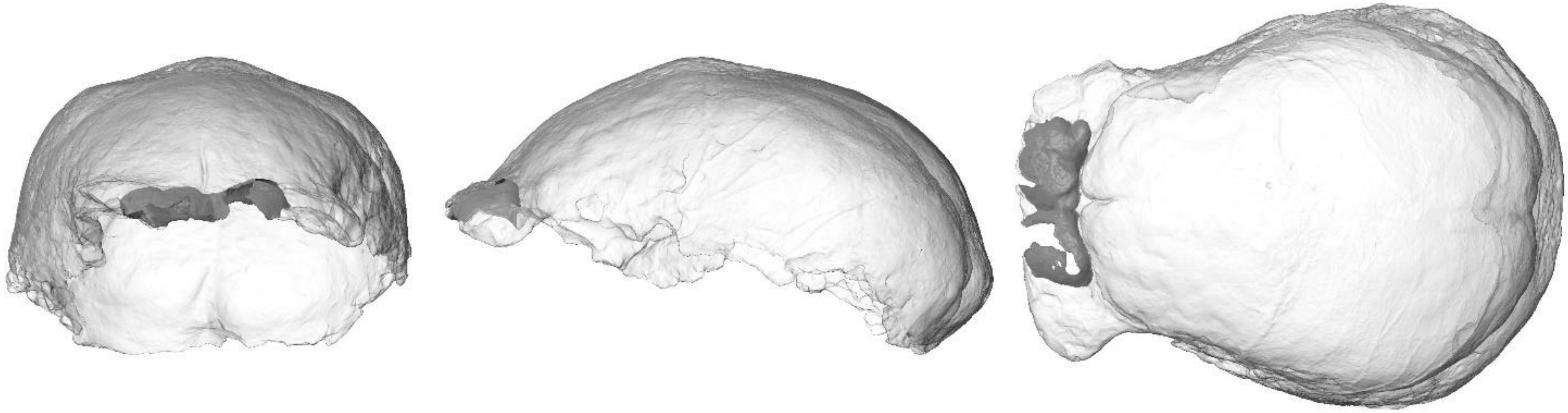

Fig. S24. Trinil, holotype of *Homo erectus*

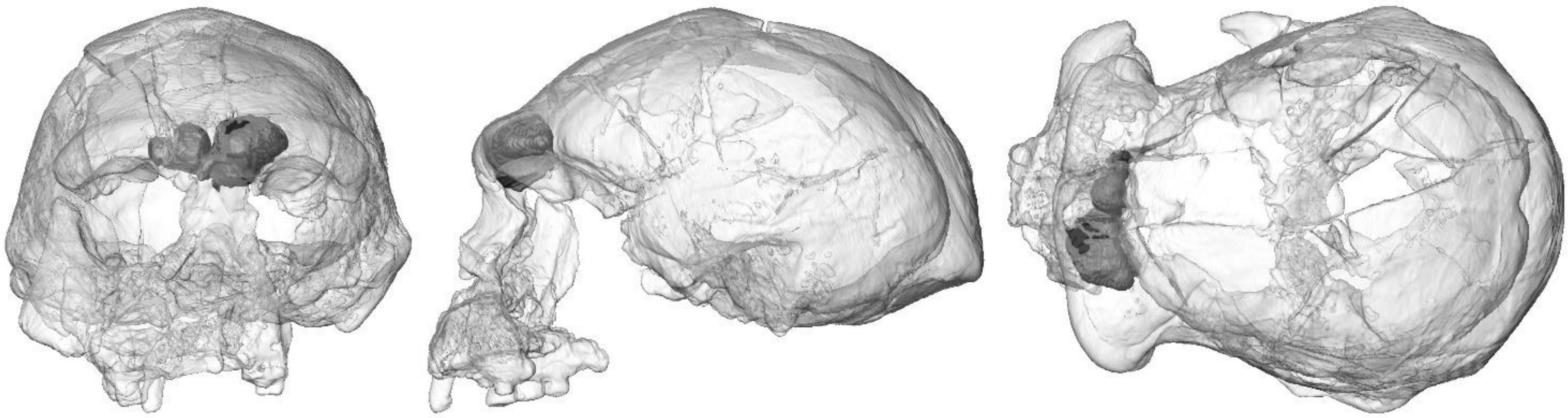

Fig. S25. Sangiran 17, *Homo erectus*

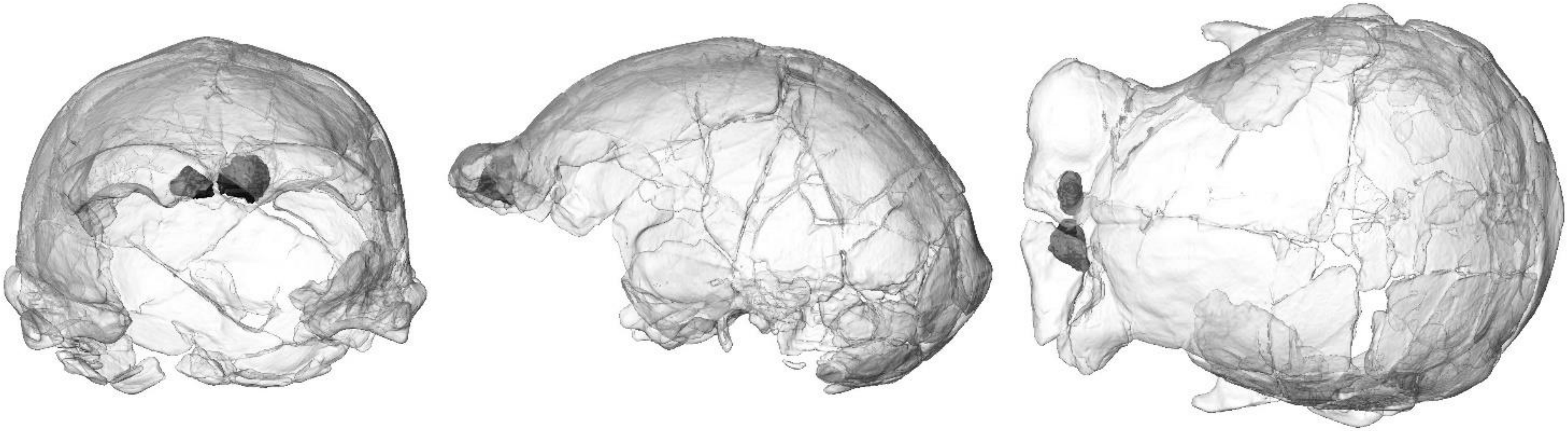

Fig. S26. Skull IX, *Homo erectus*

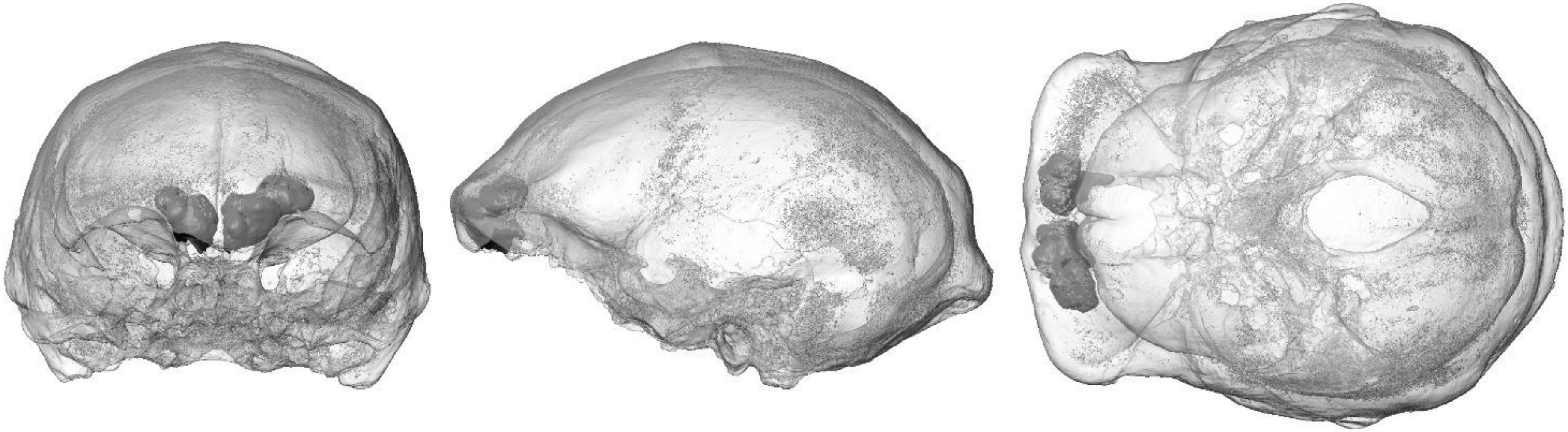

Fig. S27. Sambungmacan 4, *Homo erectus*

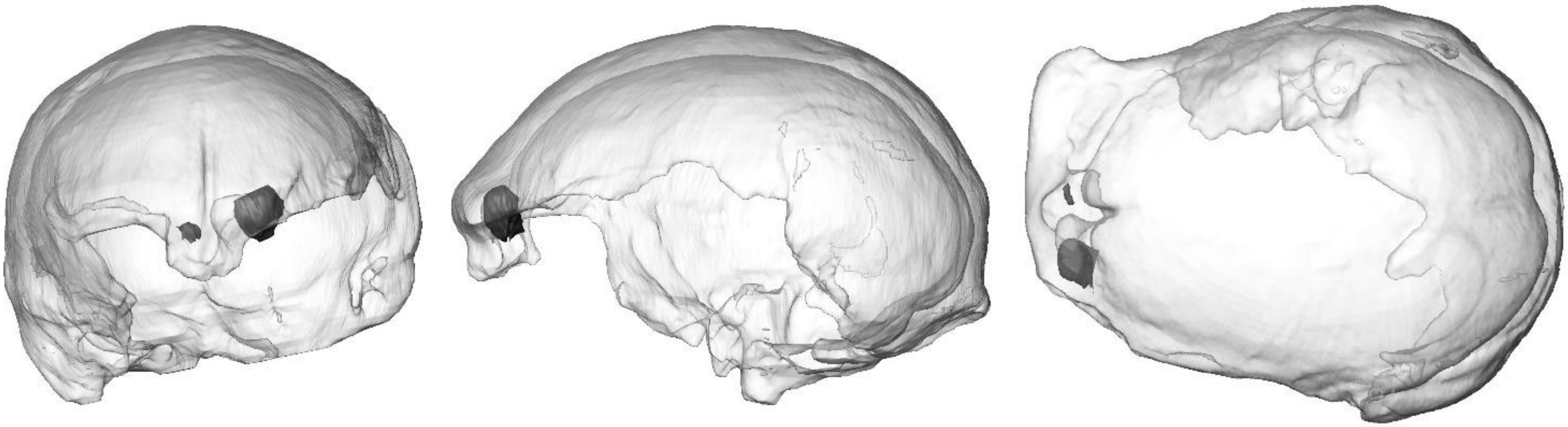

Fig. S28. Ngandong 1, *Homo erectus*

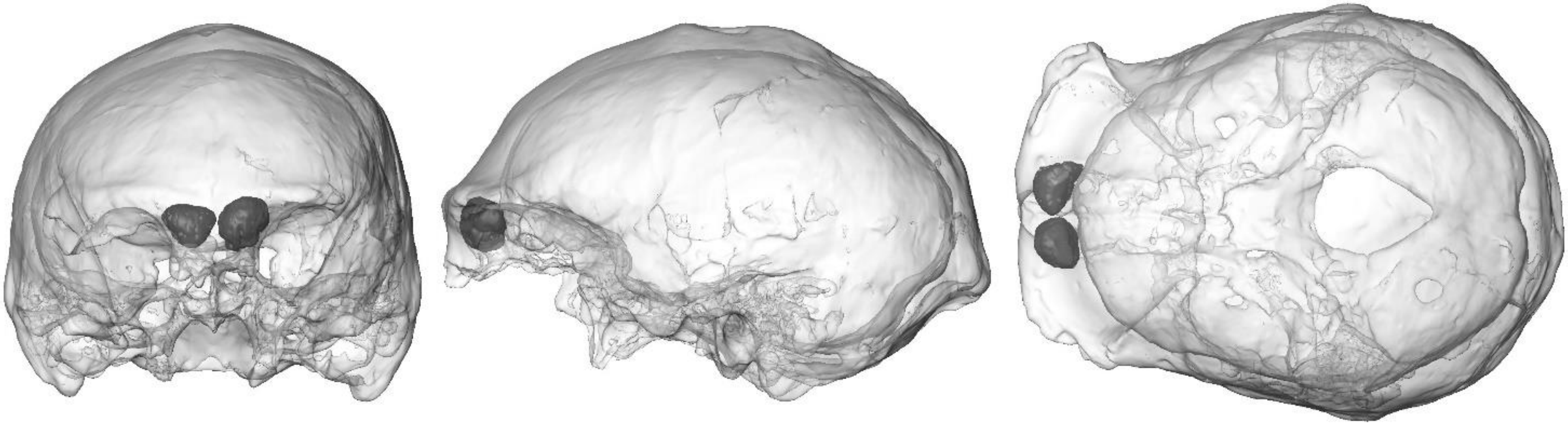

Fig. S29. Ngandong 7, *Homo erectus*

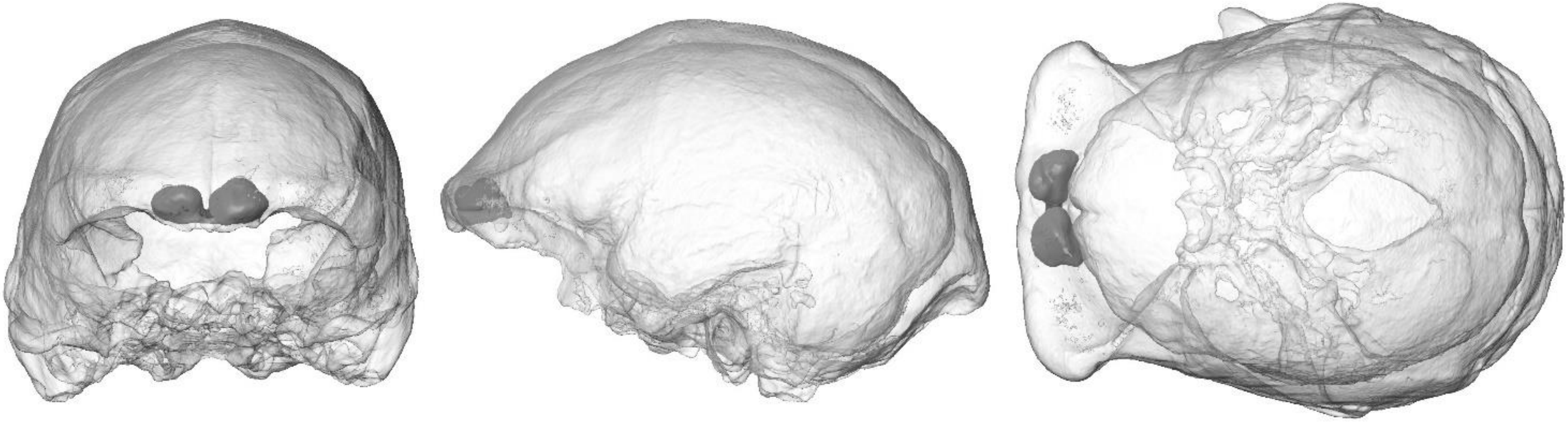

Fig. S30. Ngandong 12, *Homo erectus*

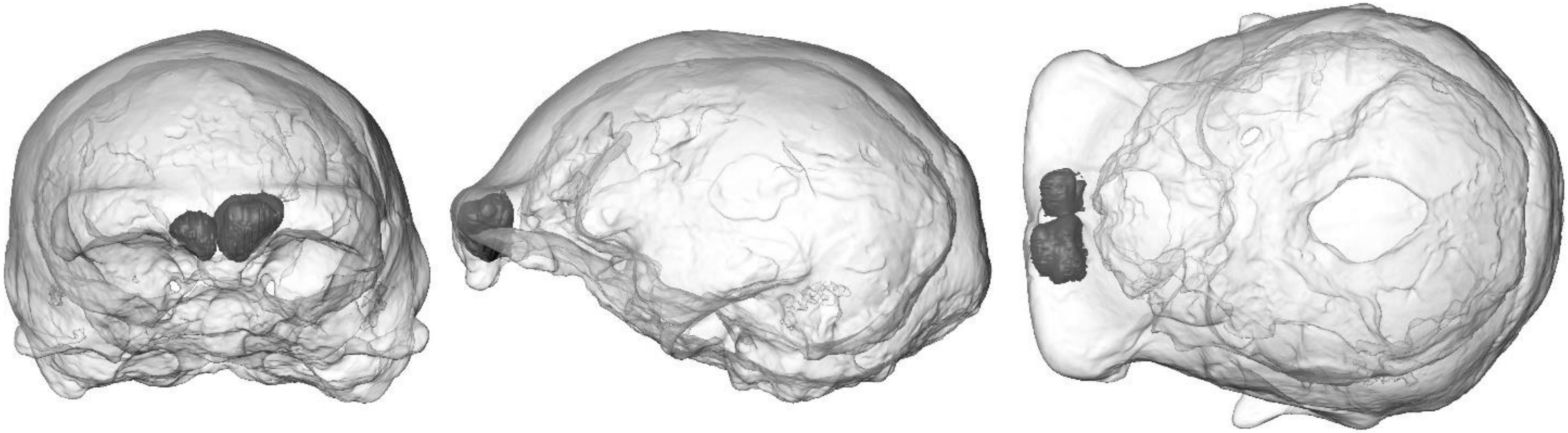

Fig. S31. Ngawi 1, *Homo erectus*

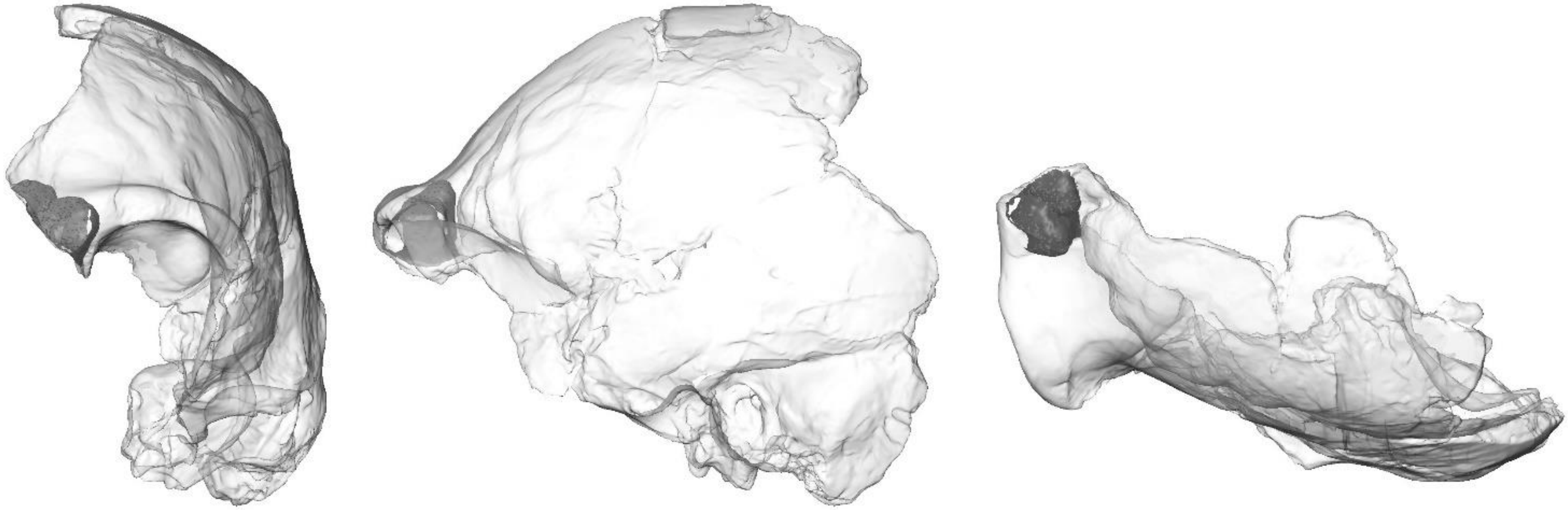

Fig. 32. DH3, *Homo naledi*

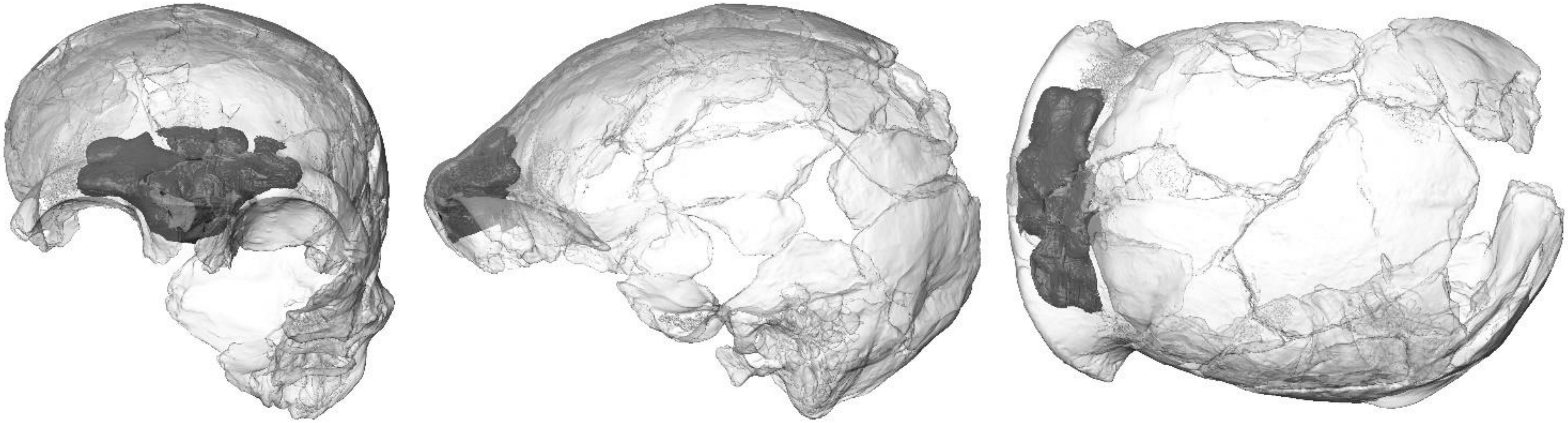

Fig. S33. LES 1, *Homo naledi*

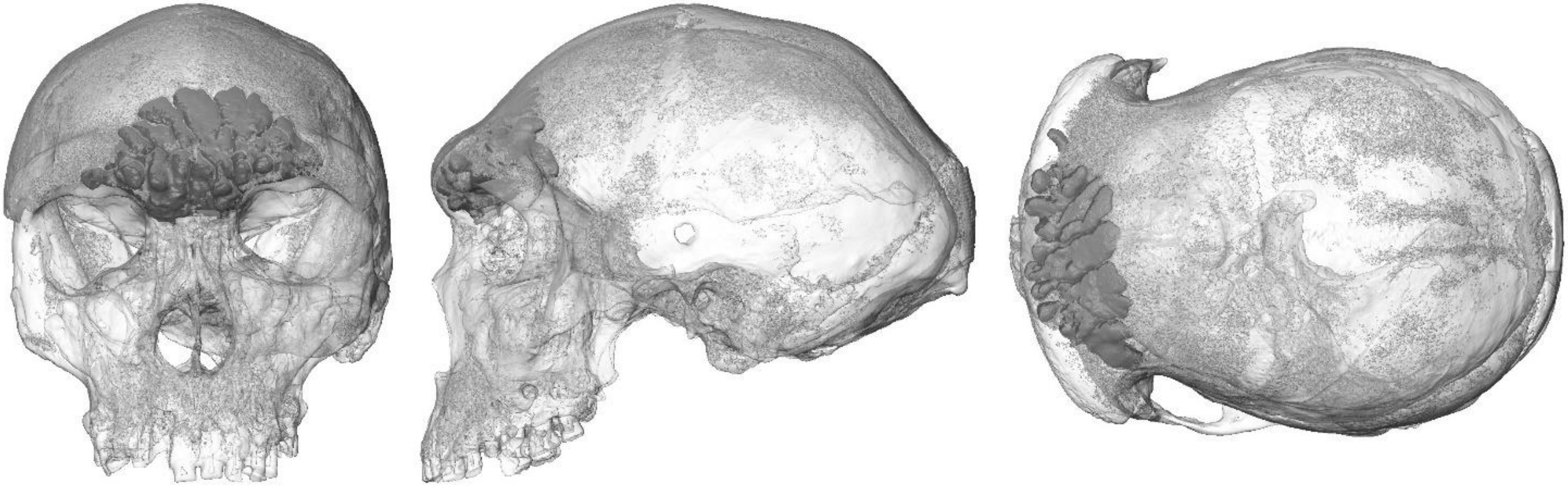

Fig. S34. Broken Hill 1 - NHMUK PA E 686, holotype of *Homo rhodesiensis*; Copyright of the Trustees of the Natural History Museum

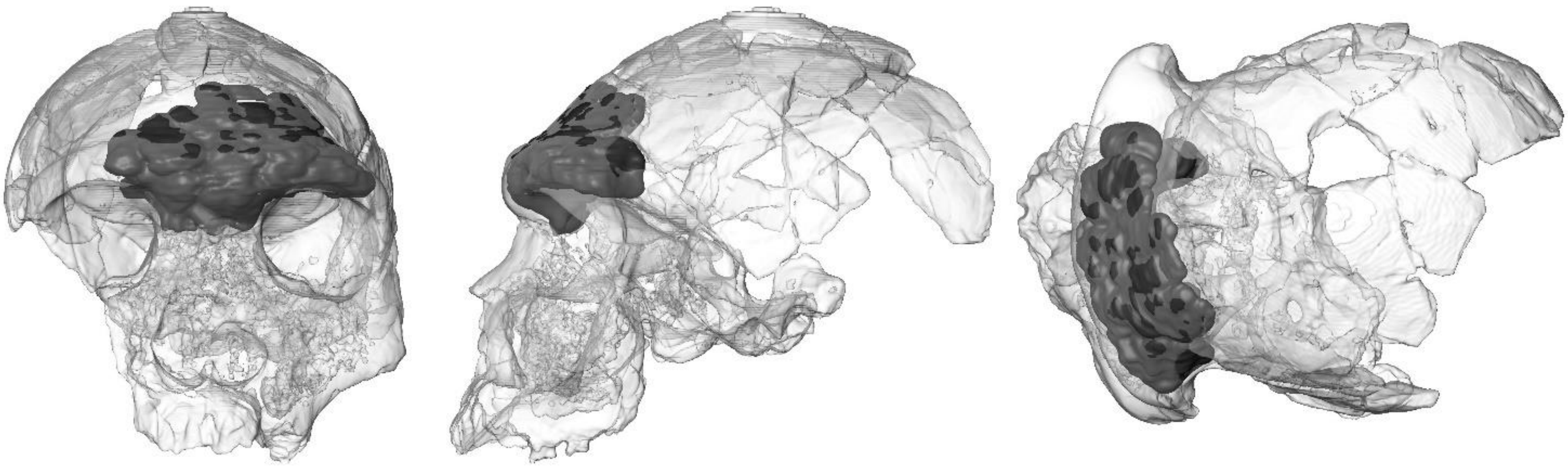

Fig. S35. Bodo, *Homo rhodesiensis*

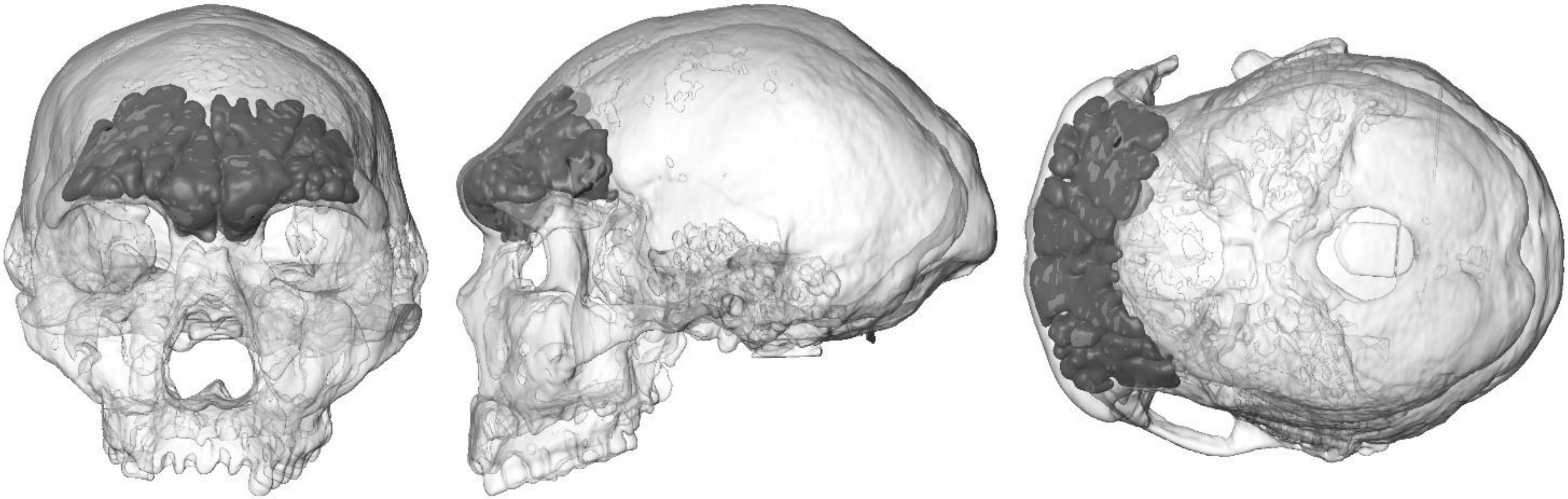

Fig. S36. Petralona, *Homo rhodesiensis*

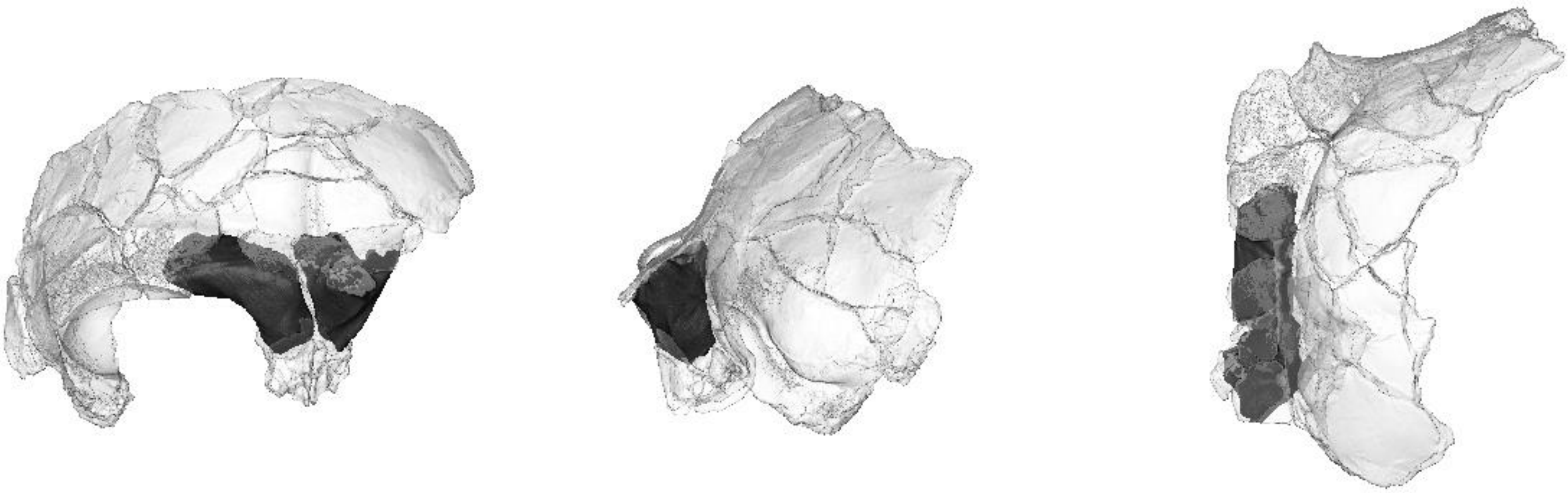

Fig. S37. TD6-15, *Homo antecessor*

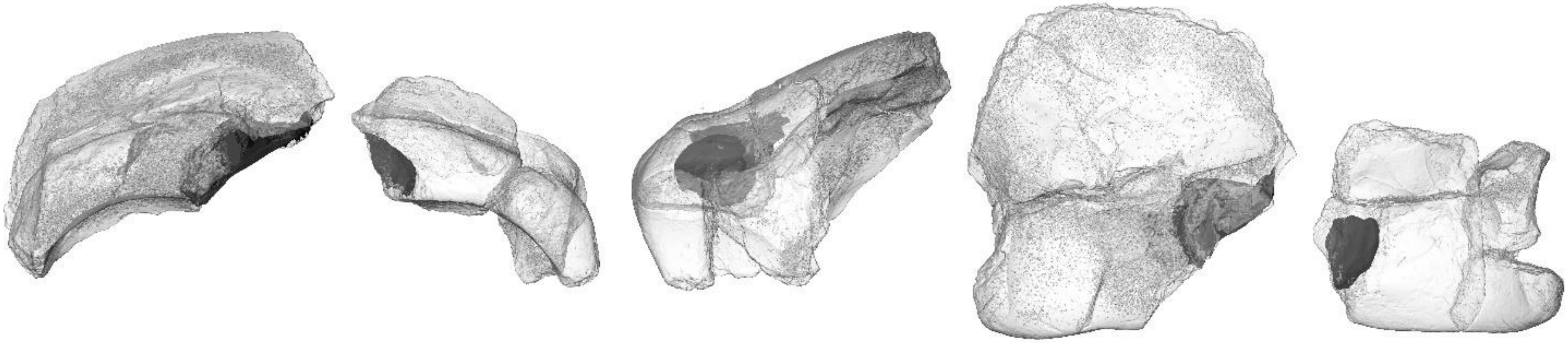

Fig. S38. HK 87 and HK 7573 (form one individual), Middle Pleistocene hominin

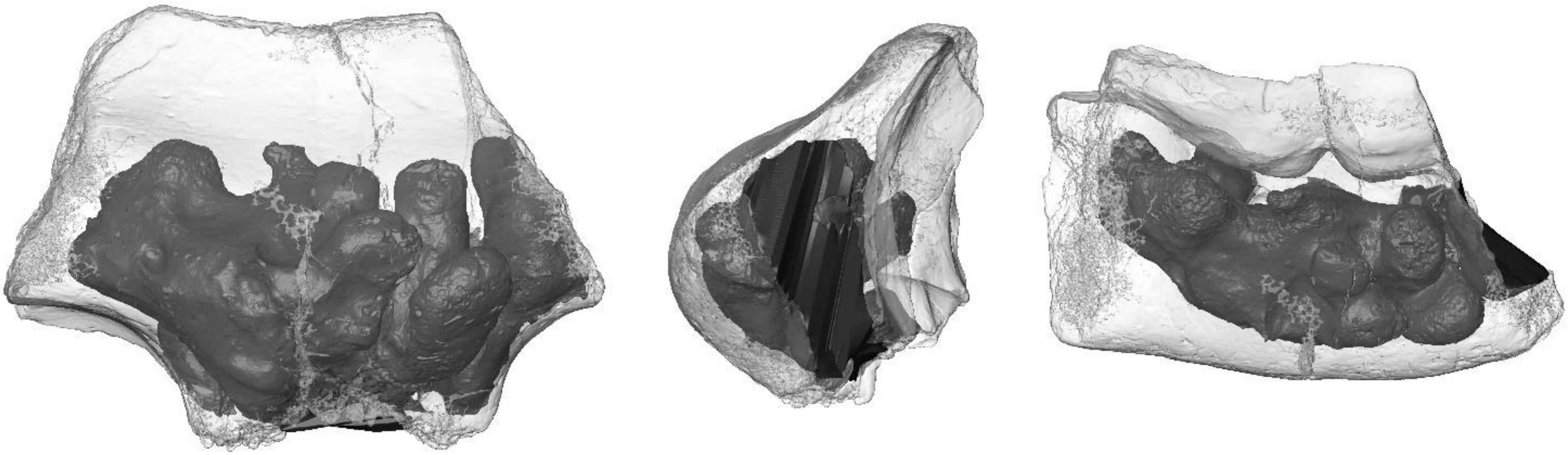

Fig. S39. Bilzinglseben HK75 199, Middle Pleistocene hominin

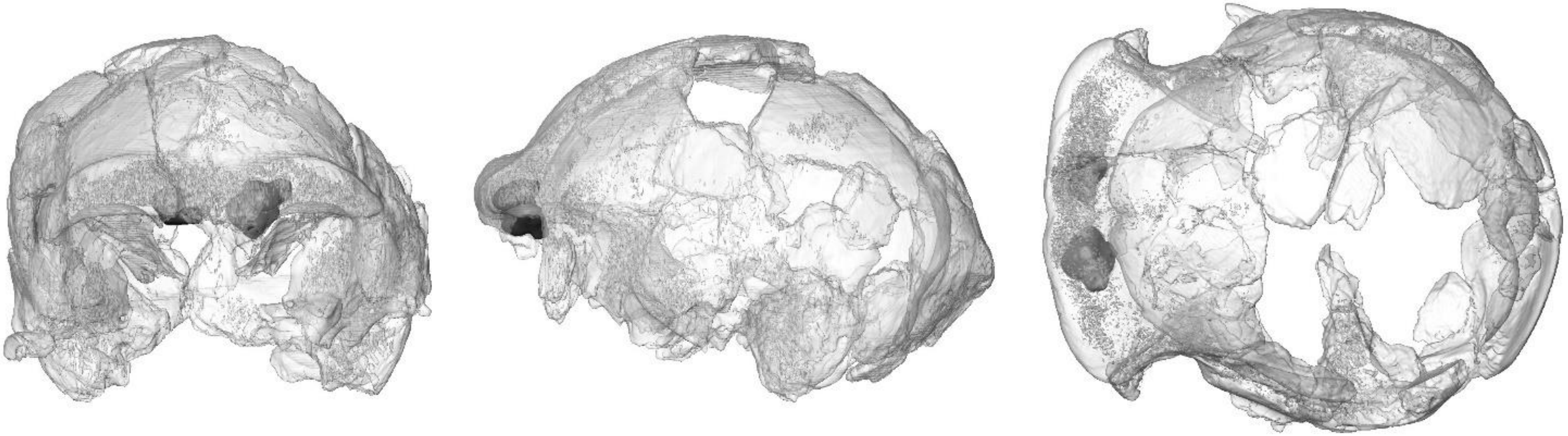

Fig. S40. Ceprano, Middle Pleistocene hominin

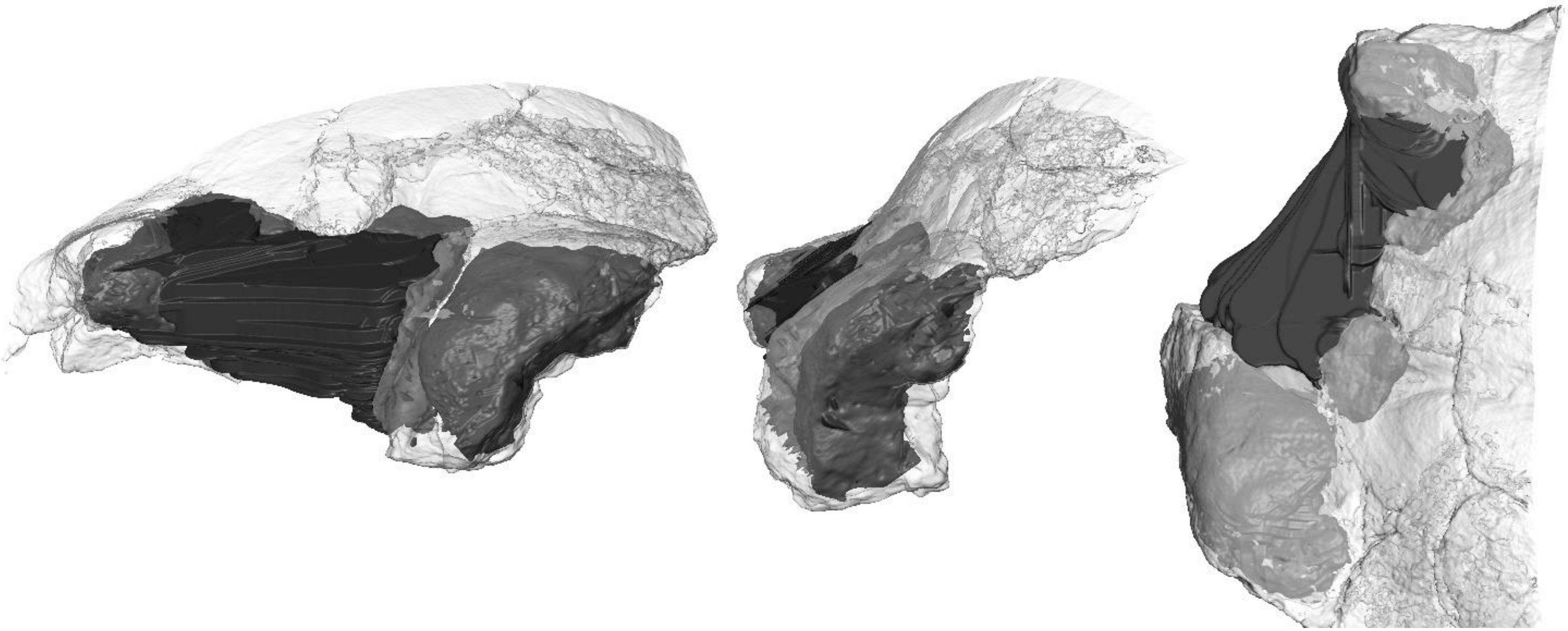

Fig. S41. Ehringsdorf H1024, Middle Pleistocene hominin

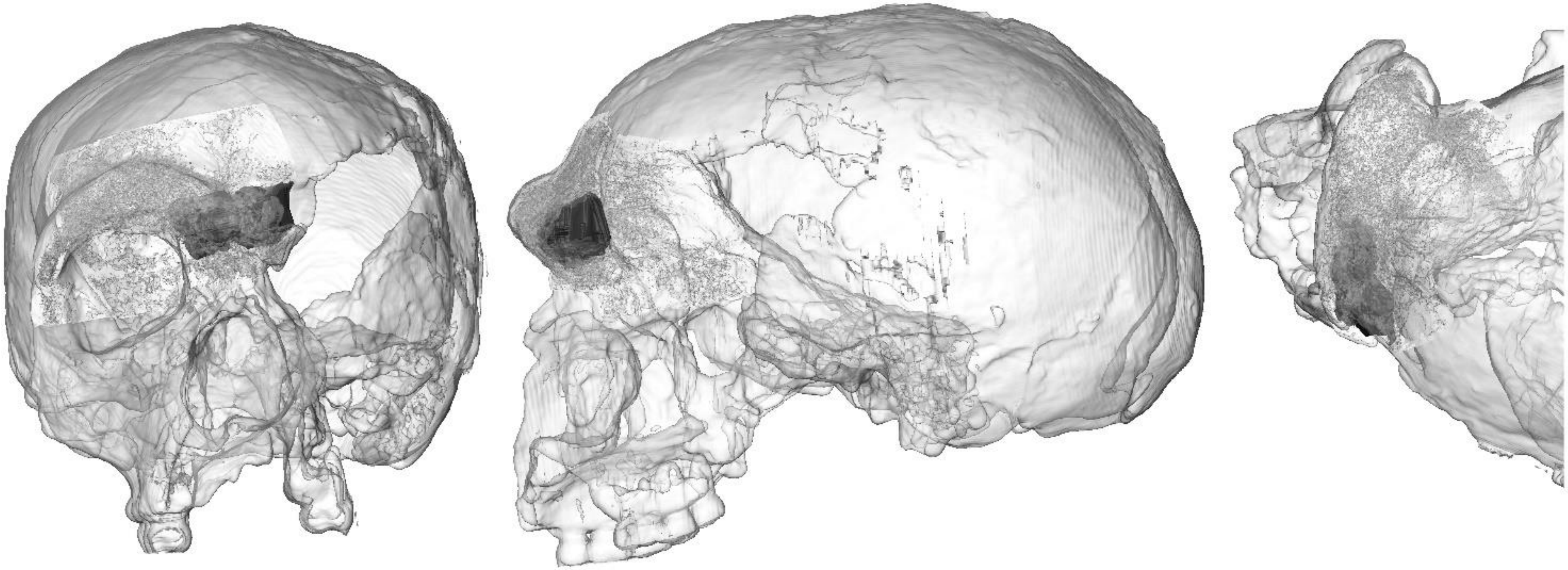

Fig. S42. Steinheim, Middle Pleistocene hominin

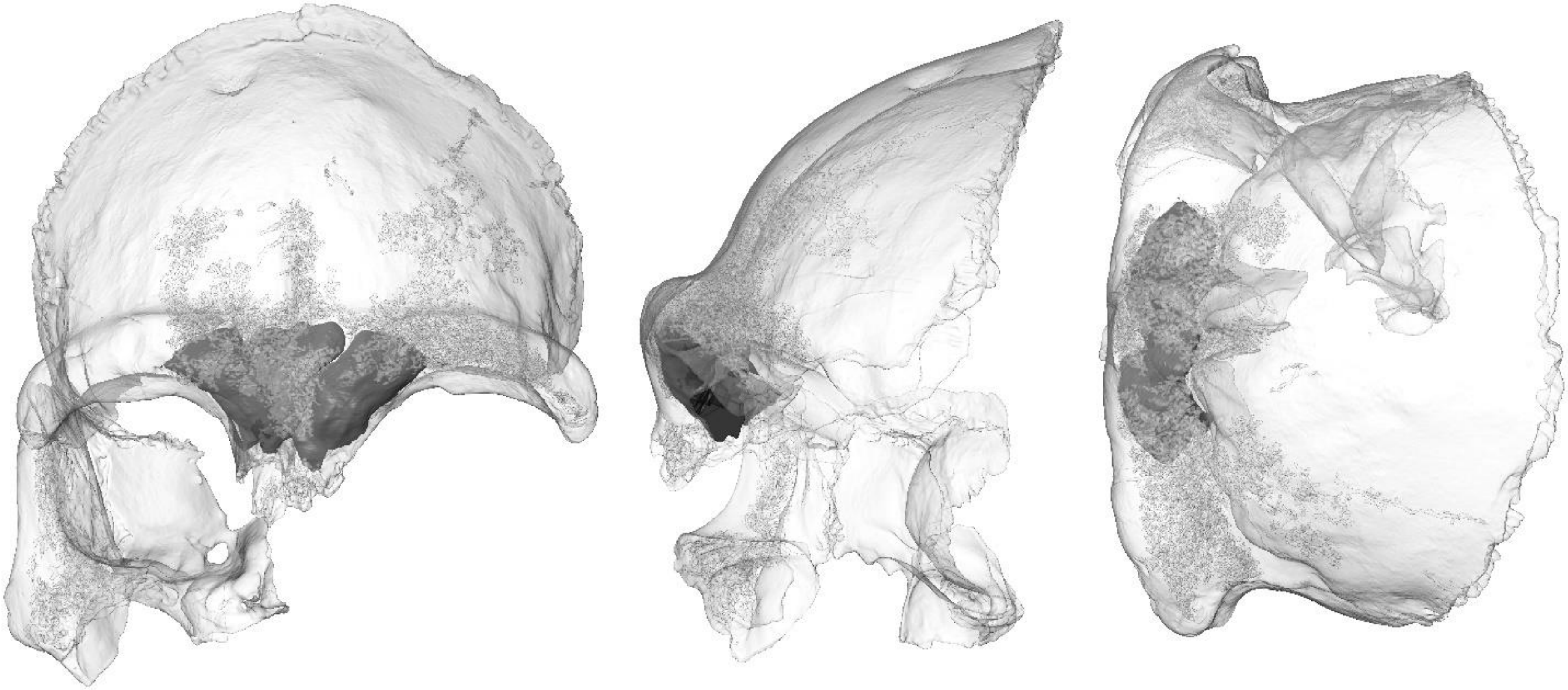

Fig. S43. Zuttiyeh, Middle Pleistocene hominin

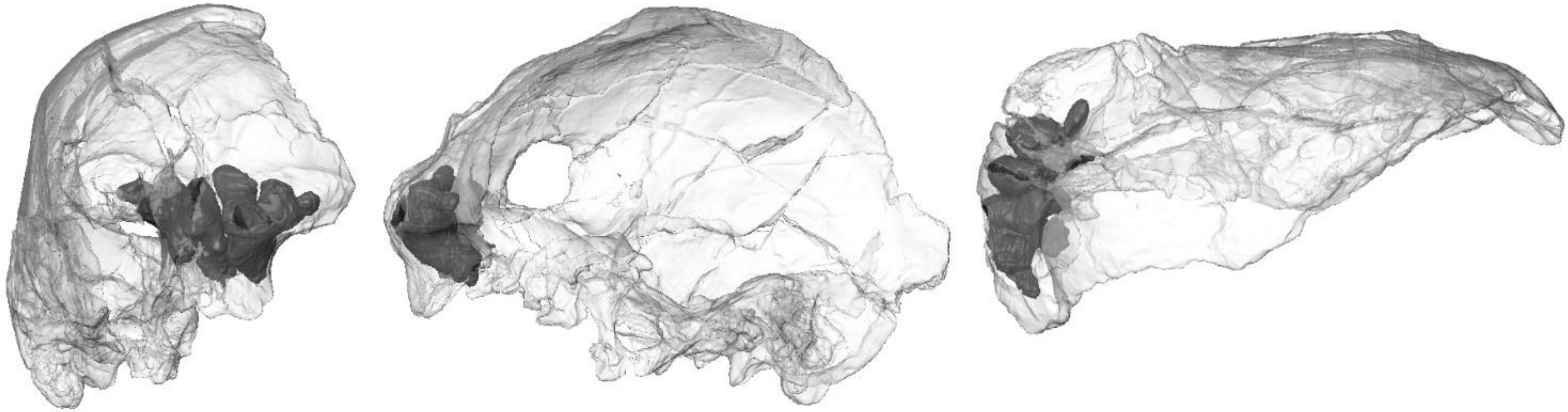

Fig. S44. Aroeira, Middle Pleistocene hominin

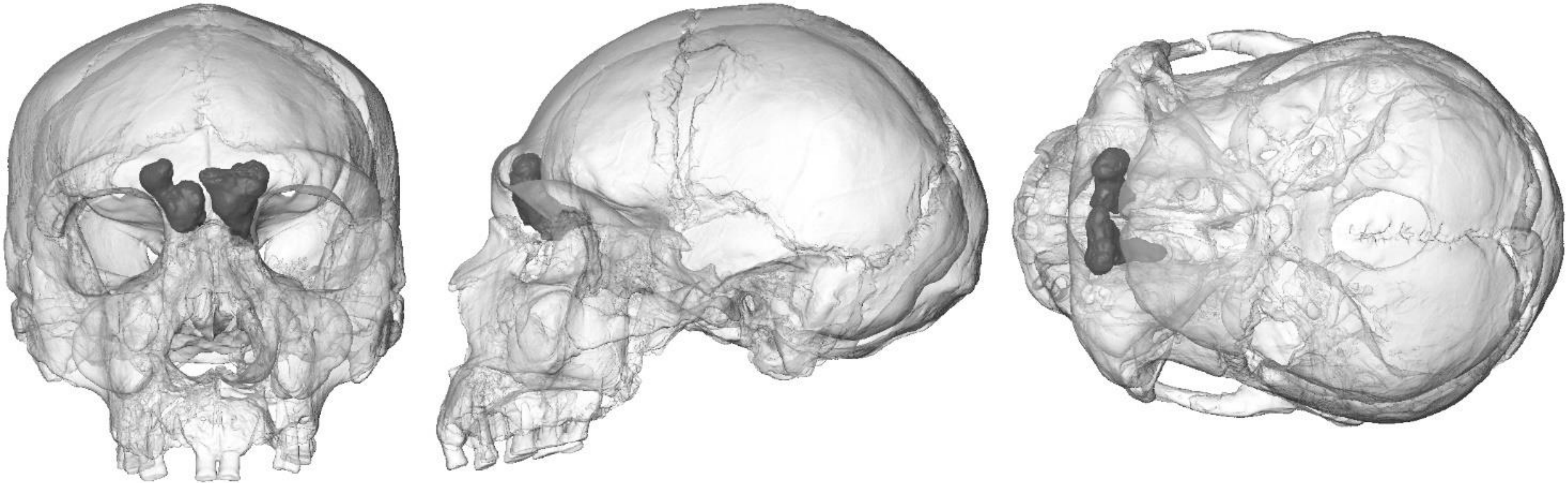

Fig. S45. Sima de los huesos skull 5, Middle Pleistocene hominin

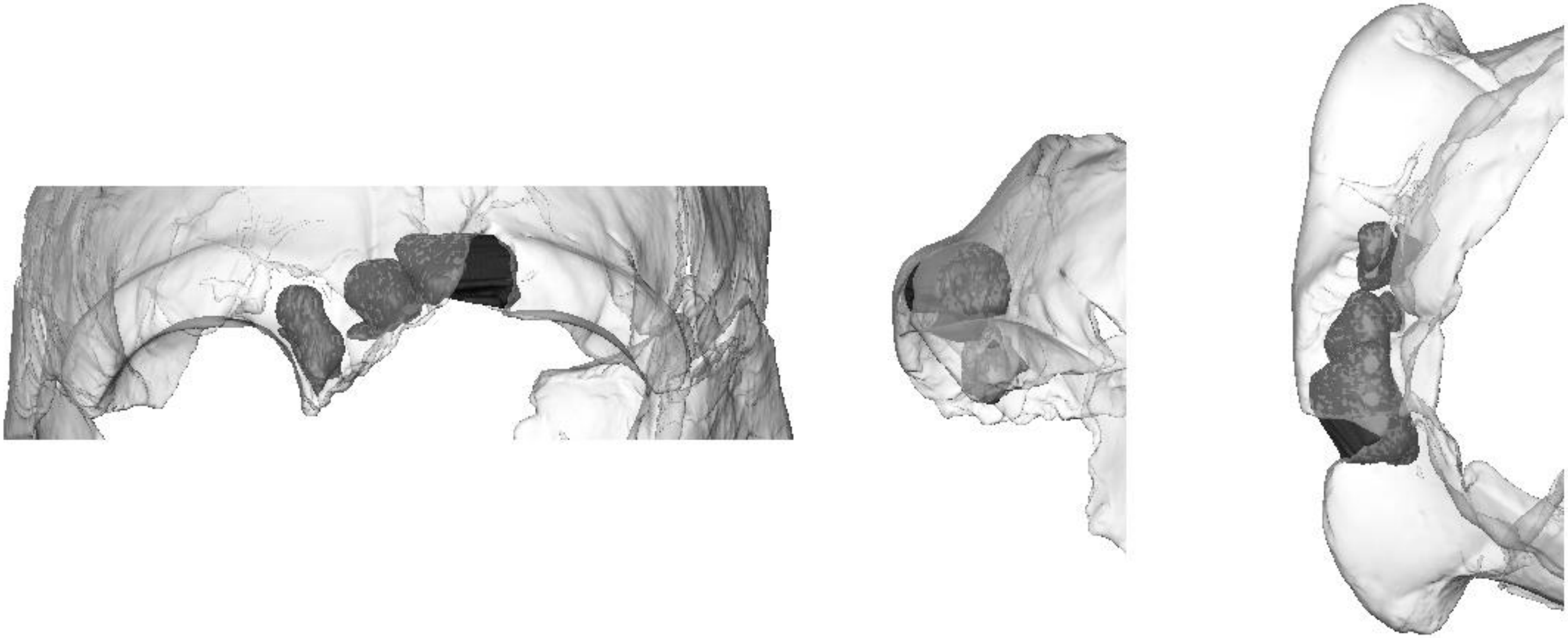

Fig. S46. Sima de los huesos skull 12, Middle Pleistocene hominin

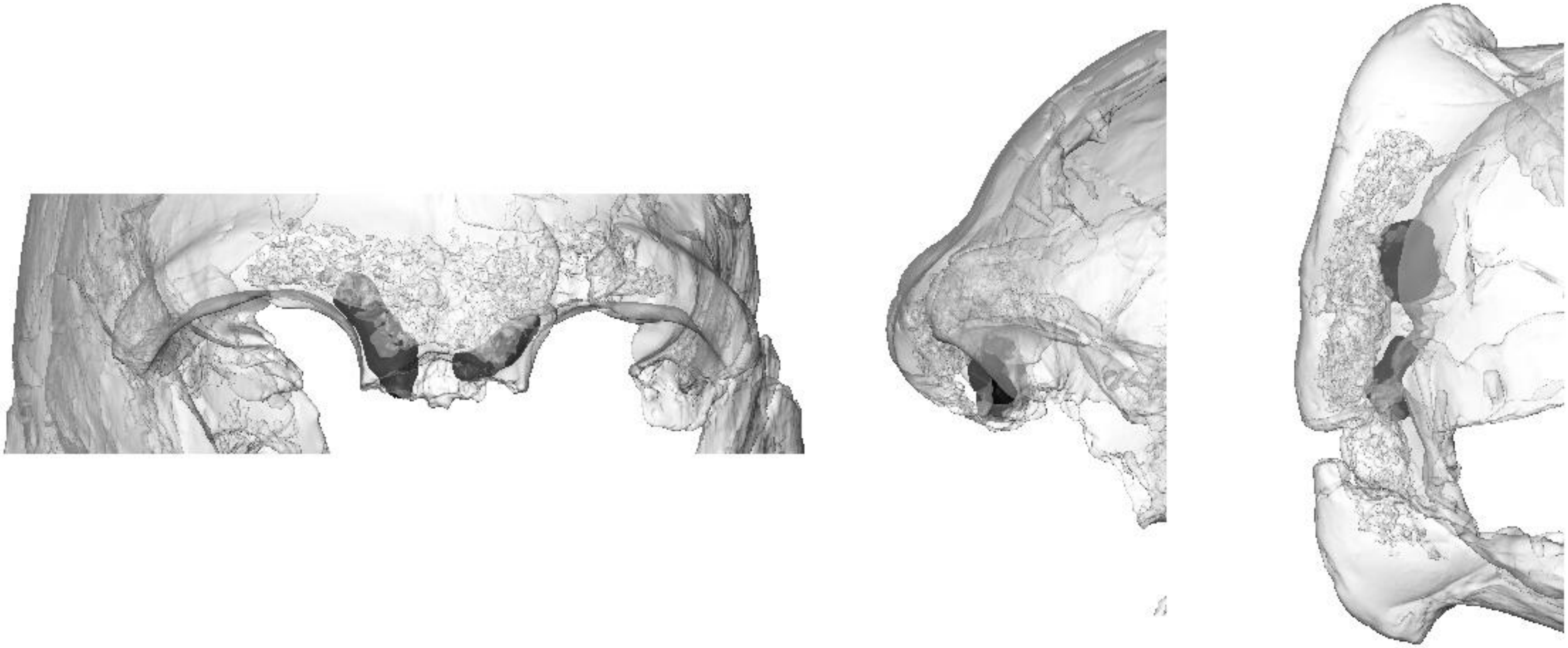

Fig. S47. Sima de los huesos skull 13, Middle Pleistocene hominin

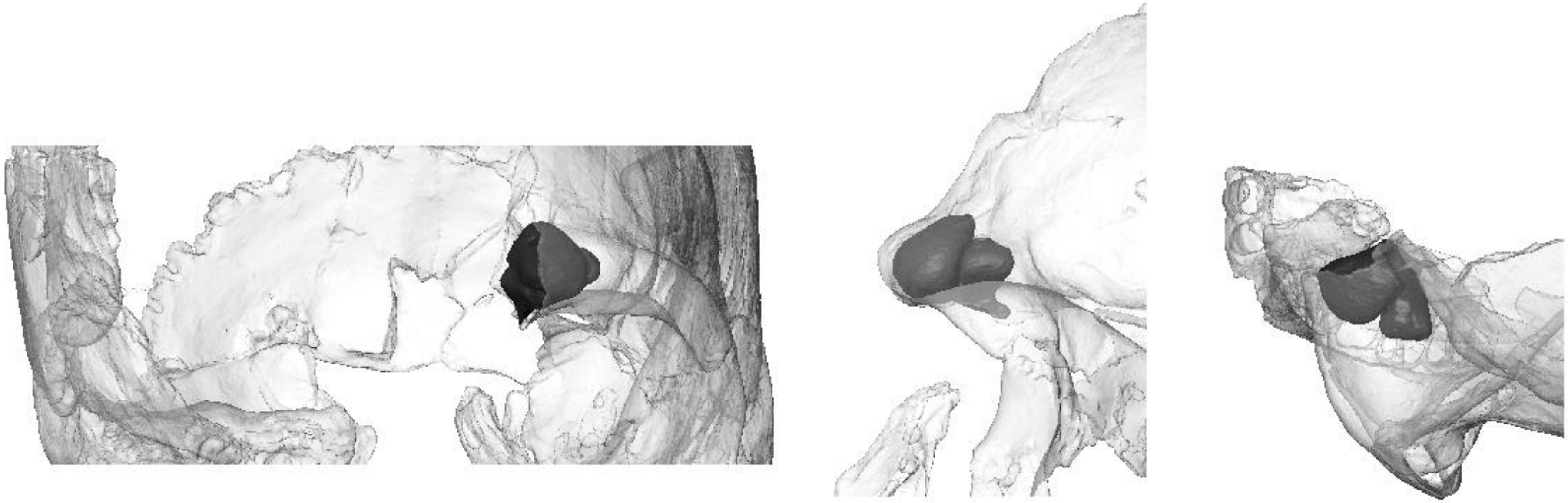

Fig. S48. Sima de los huesos skull 15, Middle Pleistocene hominin

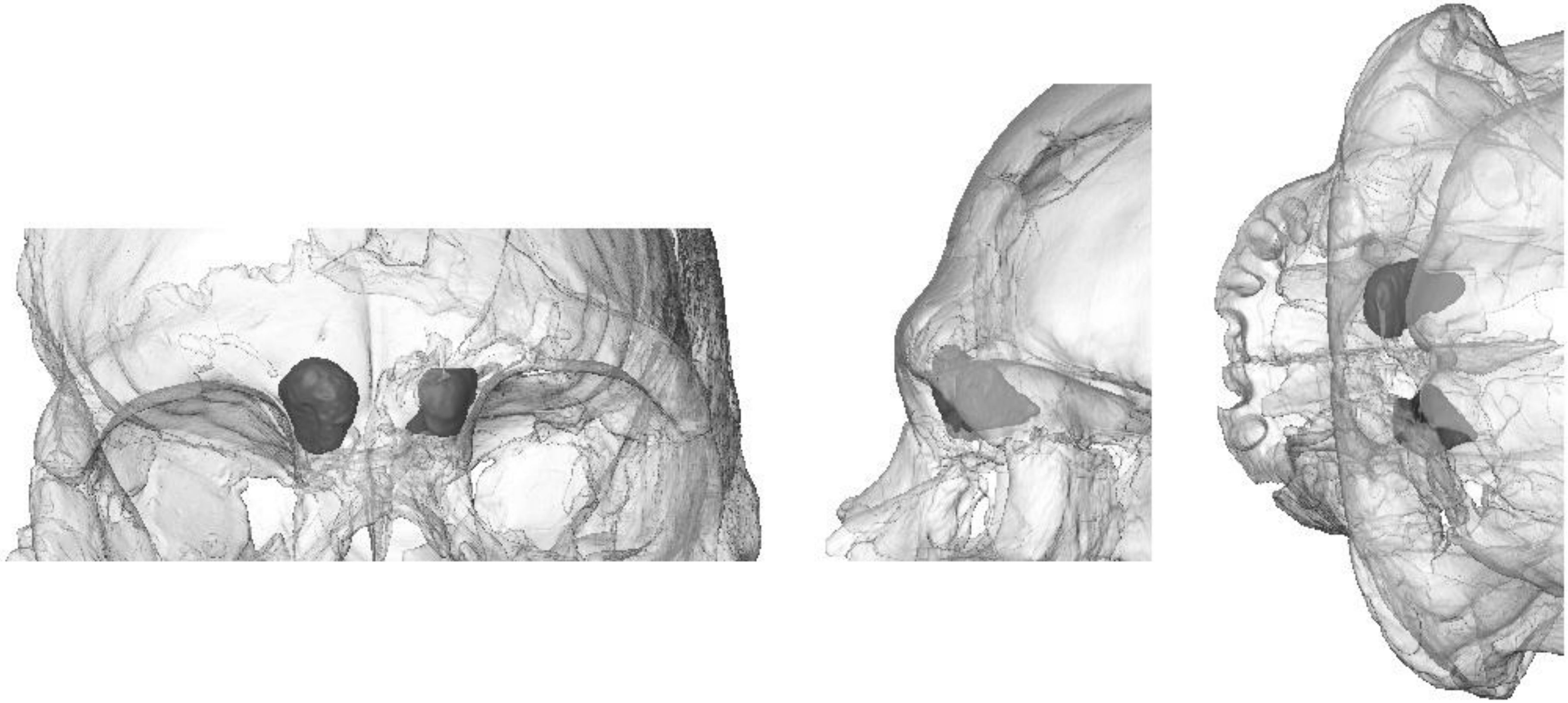

Fig. S49. Sima de los huesos skull 17, Middle Pleistocene hominin

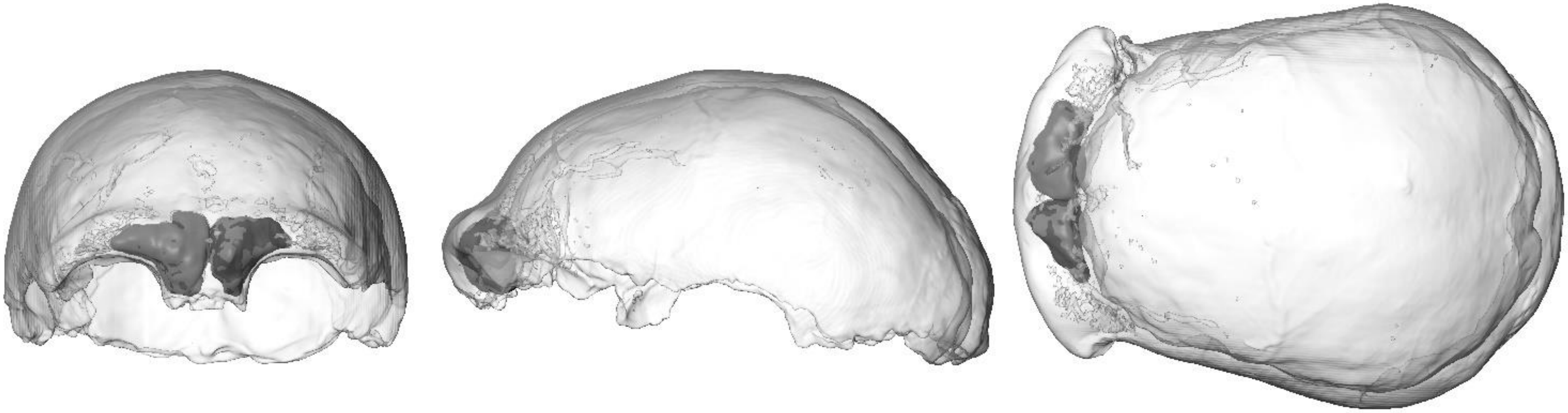

Fig. S50. Feldhofer, holotype of *Homo neanderthalensis*

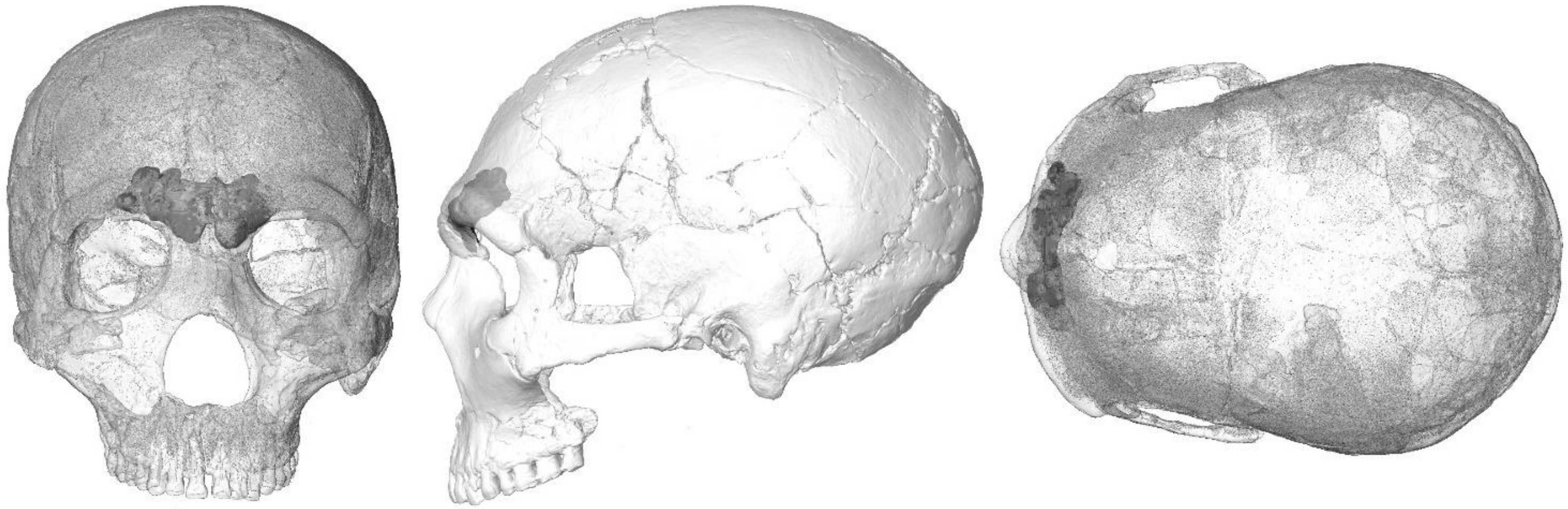

Fig. S51. Amud 1, *Homo neanderthalensis*

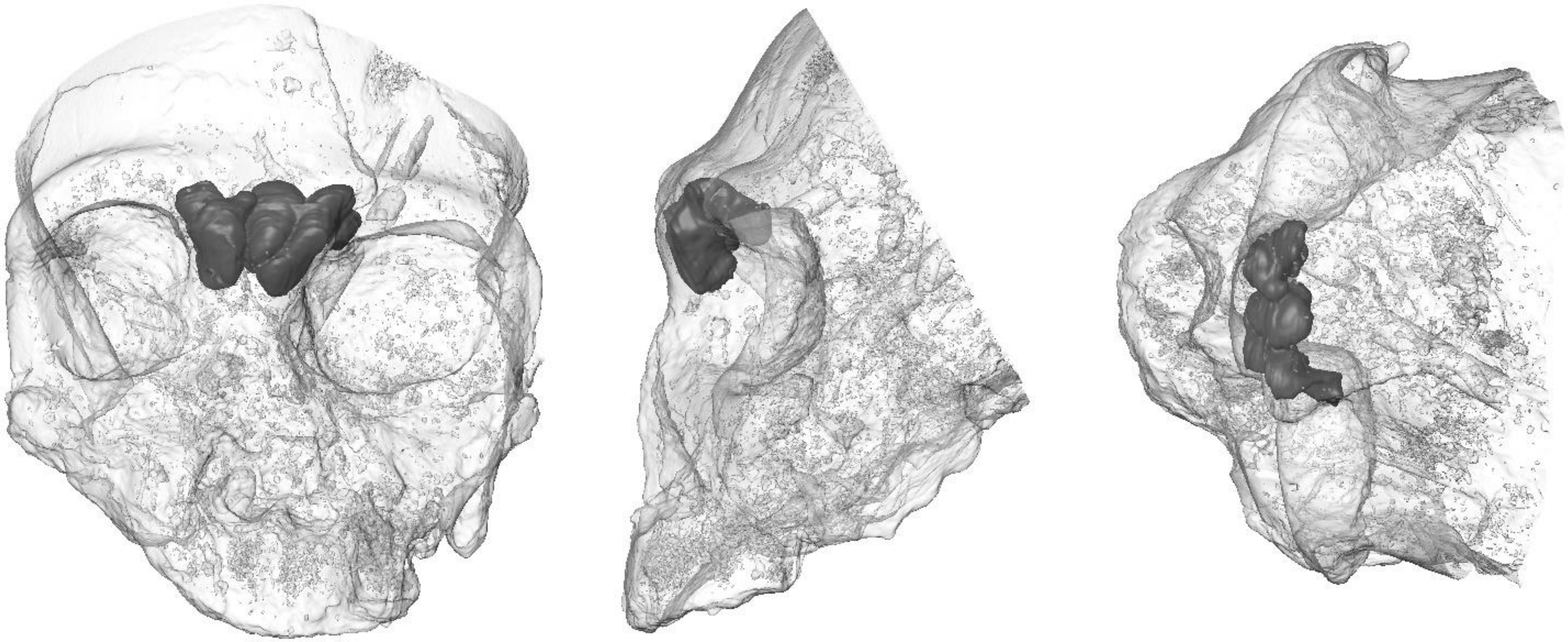

Fig. S52. Apidima 2, *Homo neanderthalensis*

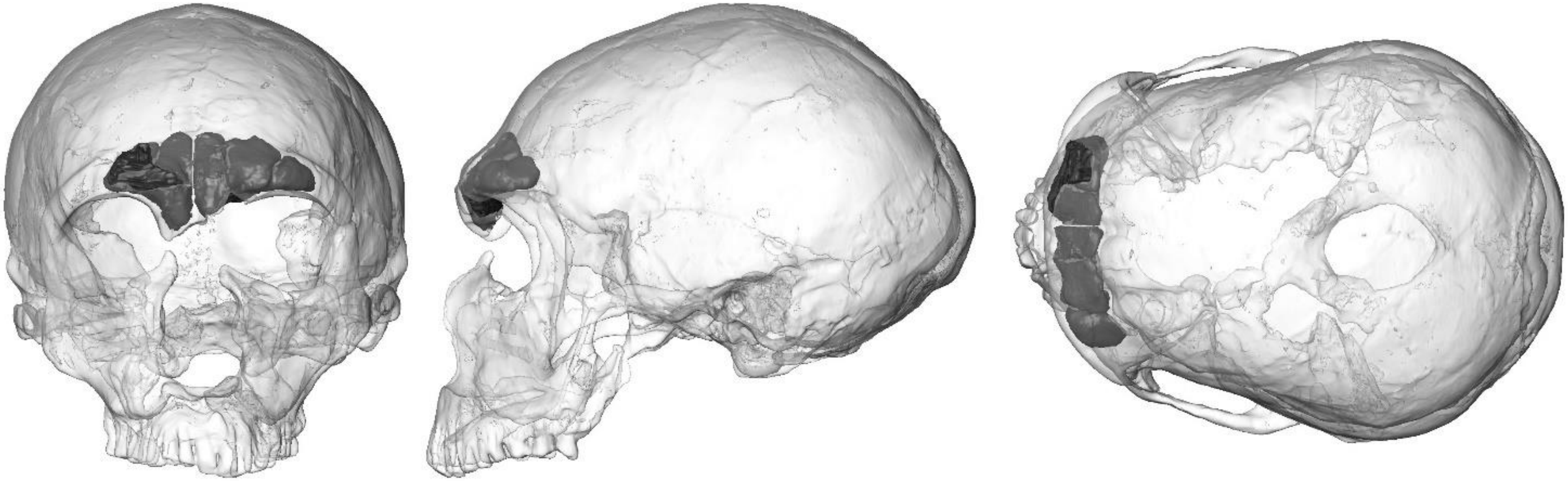

Fig. S53. La Ferrassie 1, *Homo neanderthalensis*

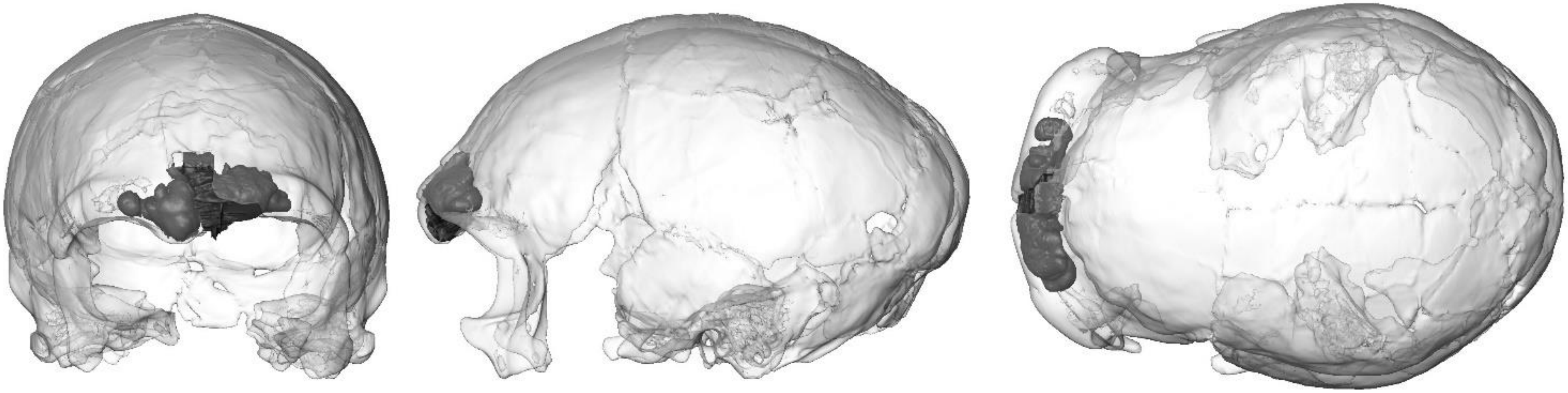

Fig. S54. La Quina H5, *Homo neanderthalensis*

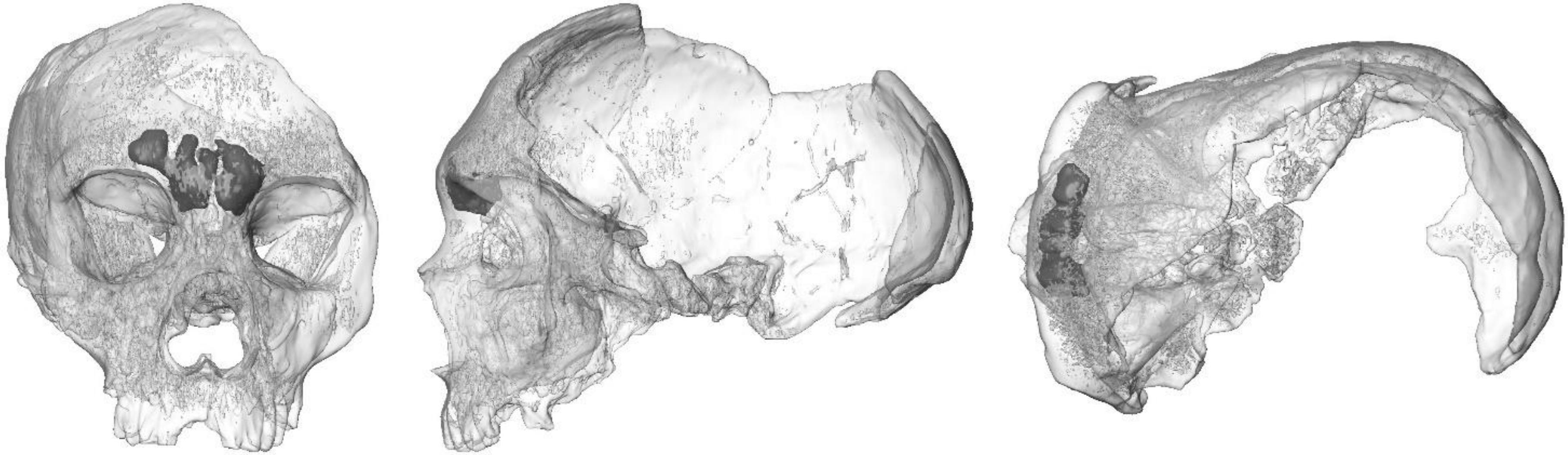

Fig. S55. Forbes' Quarry 1 - NHMUK PA EM 3811, *Homo neanderthalensis*; Copyright of the Trustees of the Natural History Museum

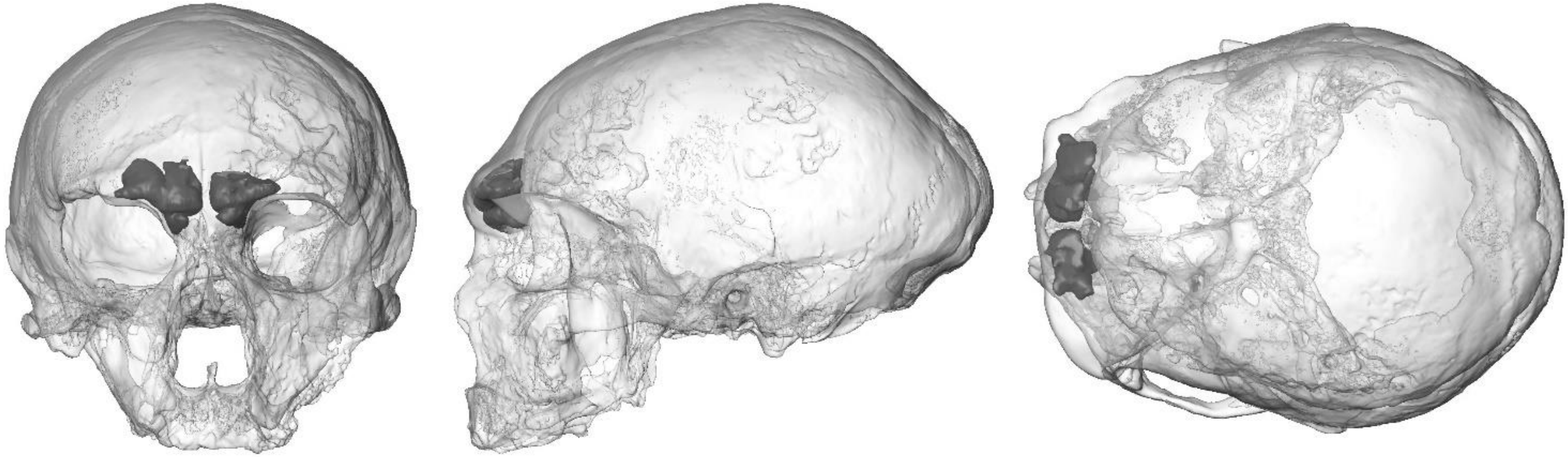

Fig. S56. Guattari 1 *Homo neanderthalensis*

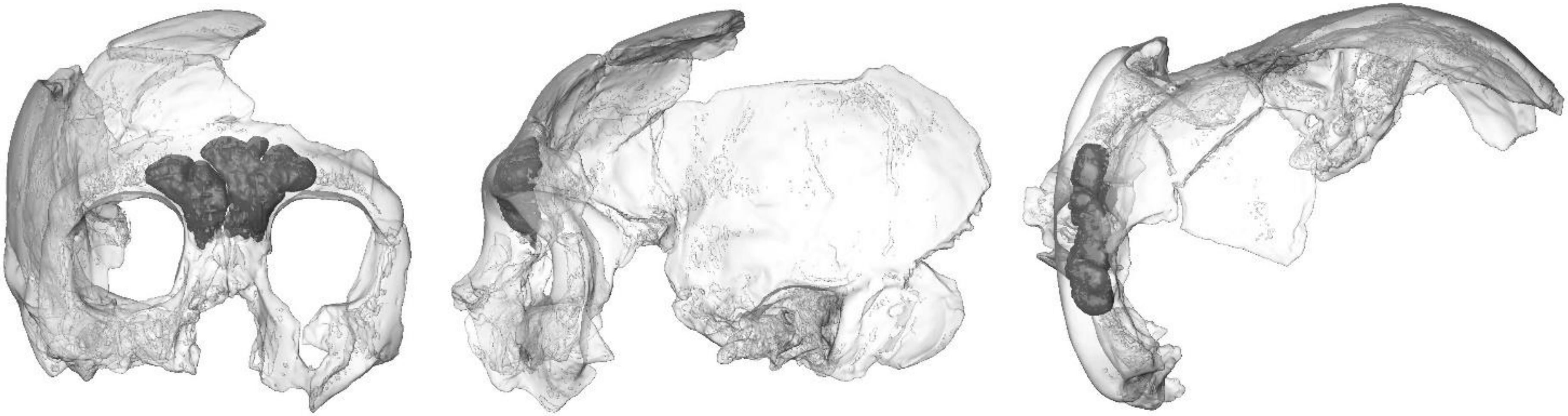

Fig. S57. Krapina 3, *Homo neanderthalensis*

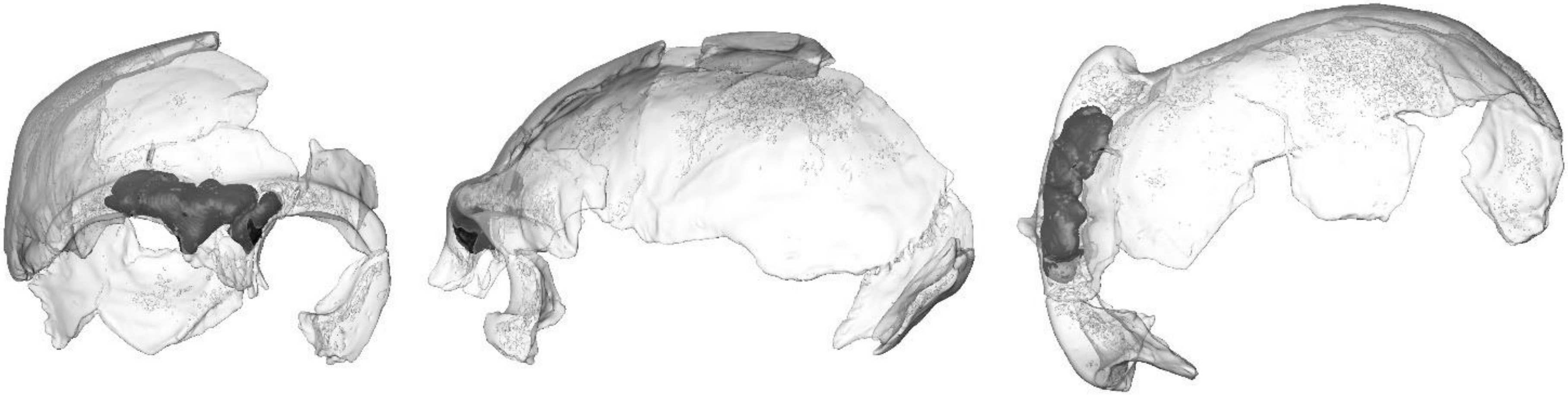

Fig. S58. Krapina 6, *Homo neanderthalensis*

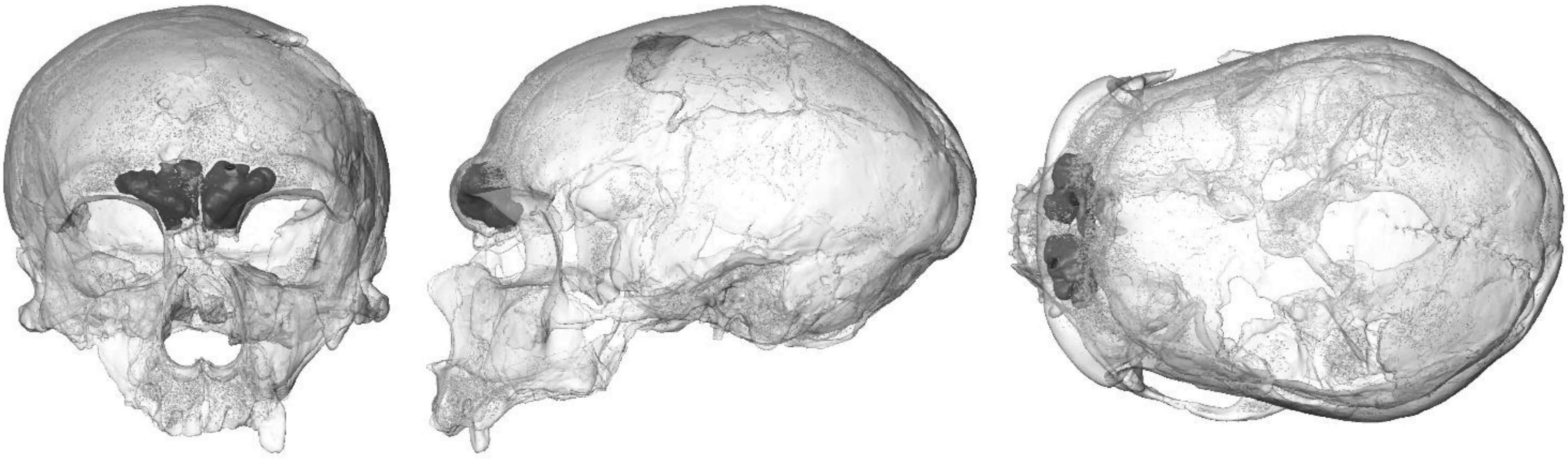

Fig. S59. La Chapelle aux Saints 1, *Homo neanderthalensis*

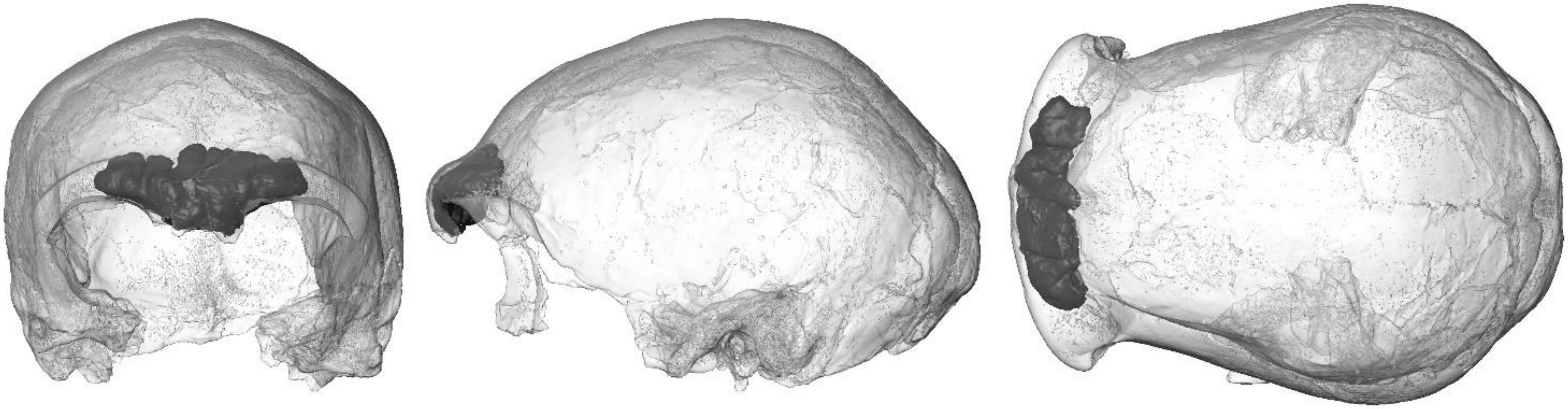

Fig. S60. Spy 1, *Homo neanderthalensis*

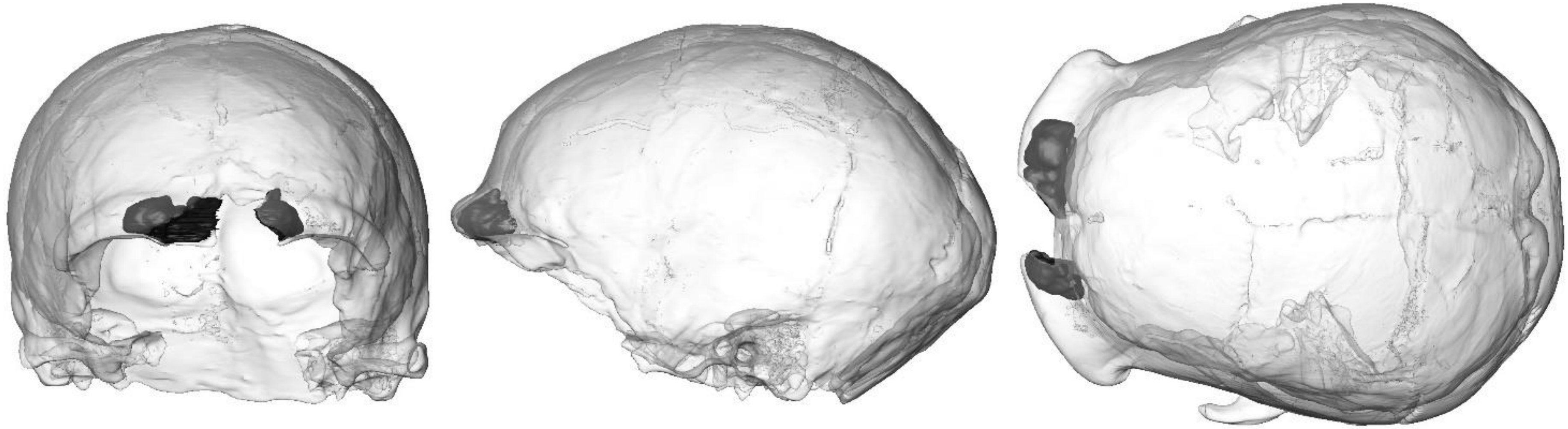

Fig. S61. Spy 10, *Homo neanderthalensis*

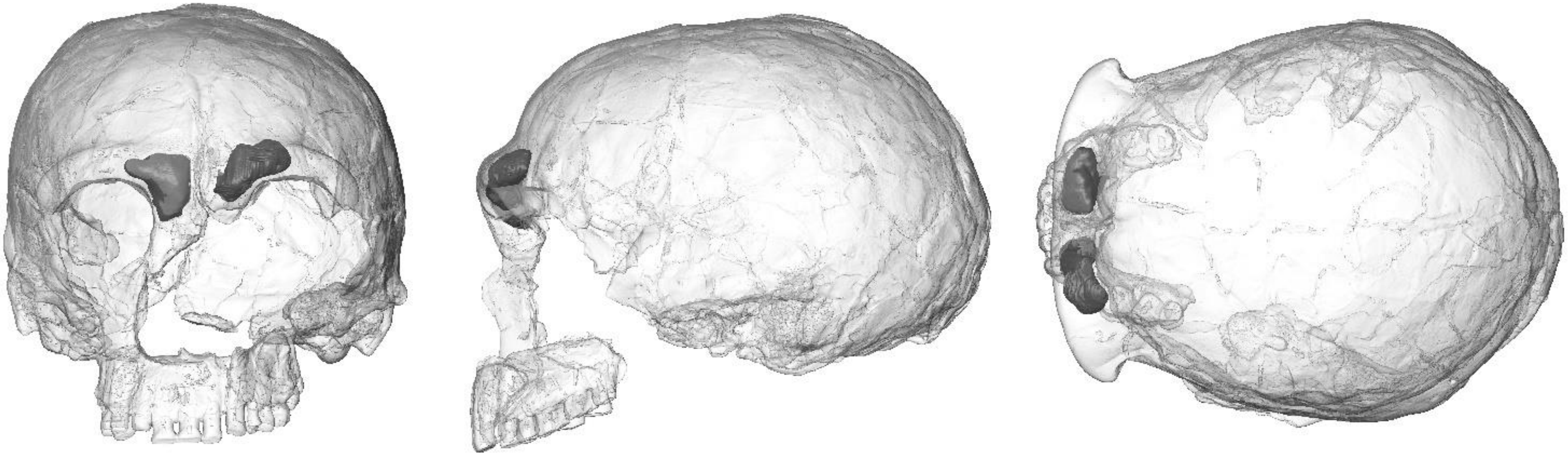

Fig. S62. Tabun C1 - NHMUK PA EM 3640, *Homo neanderthalensis*; Copyright of the Trustees of the Natural History Museum

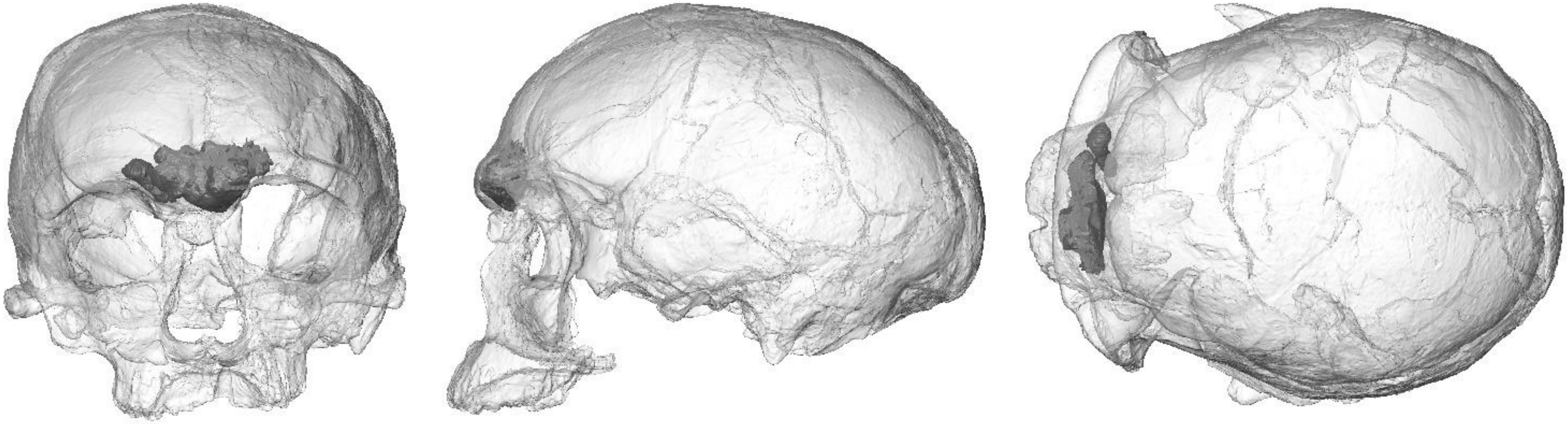

Fig. S63. Jebel Irhoud 1, *Homo sapiens*?

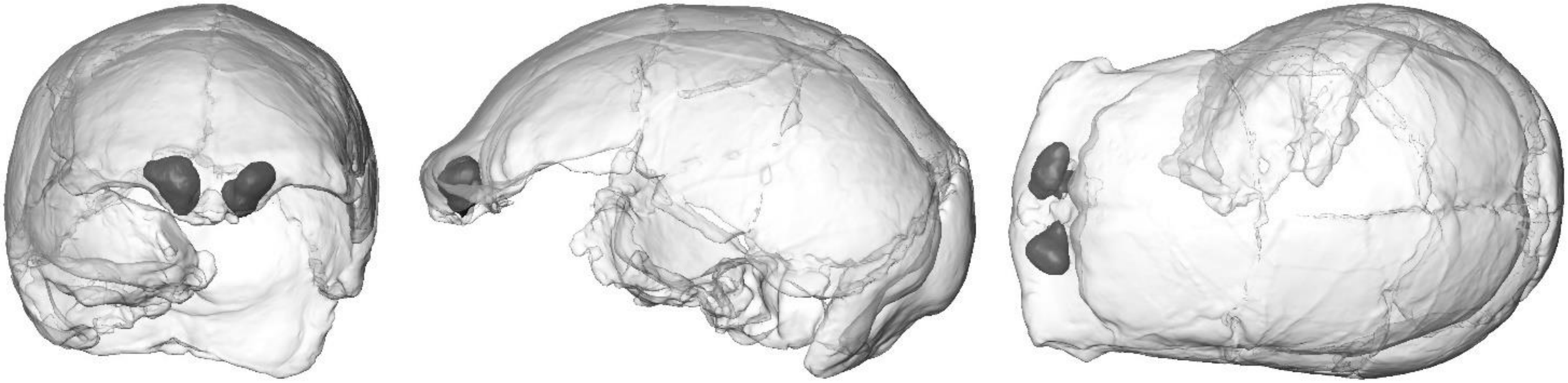

Fig. S64. LH 18, *Homo sapiens*?

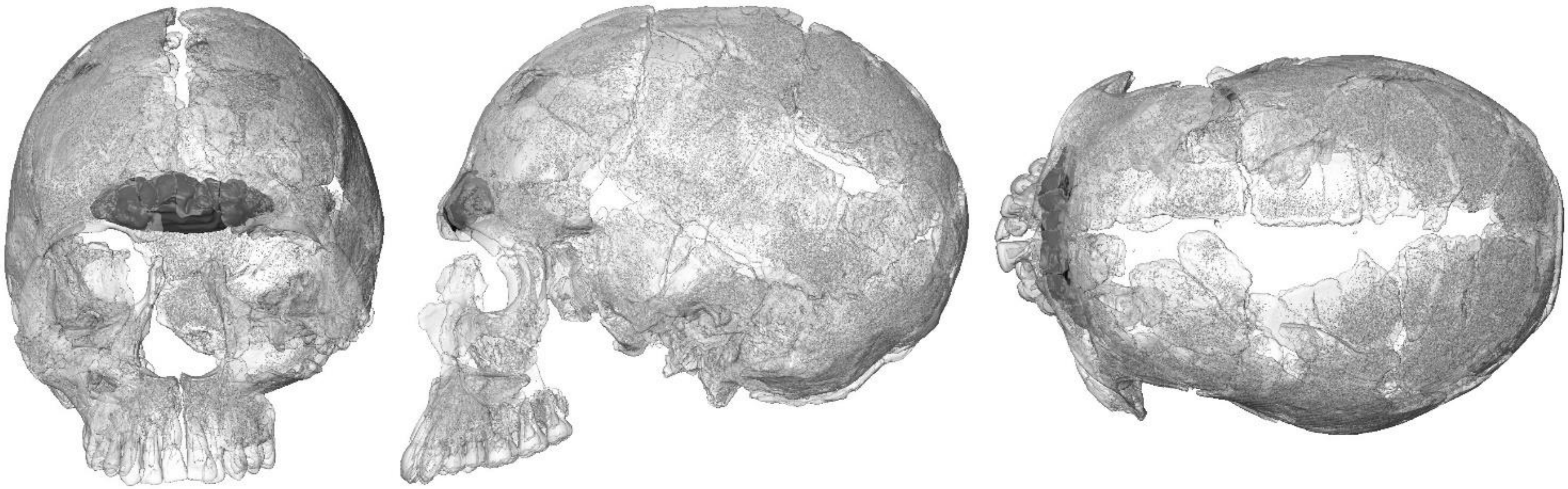

Fig. S65. Qafzeh 9, *Homo sapiens*

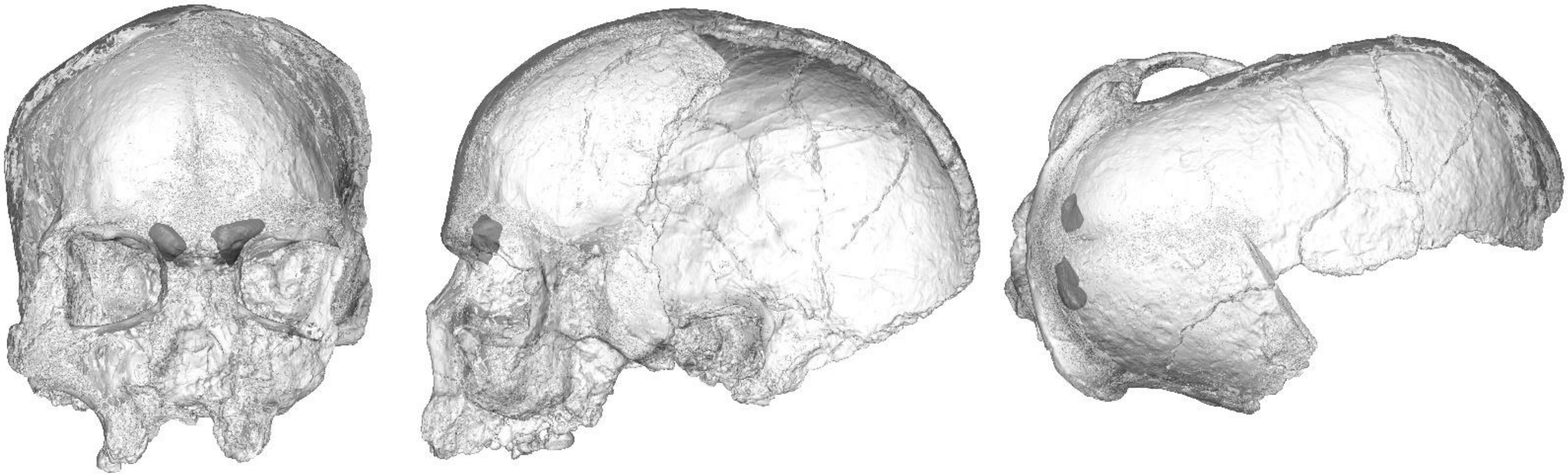

Fig. S66. Hofmeyr, *Homo sapiens*

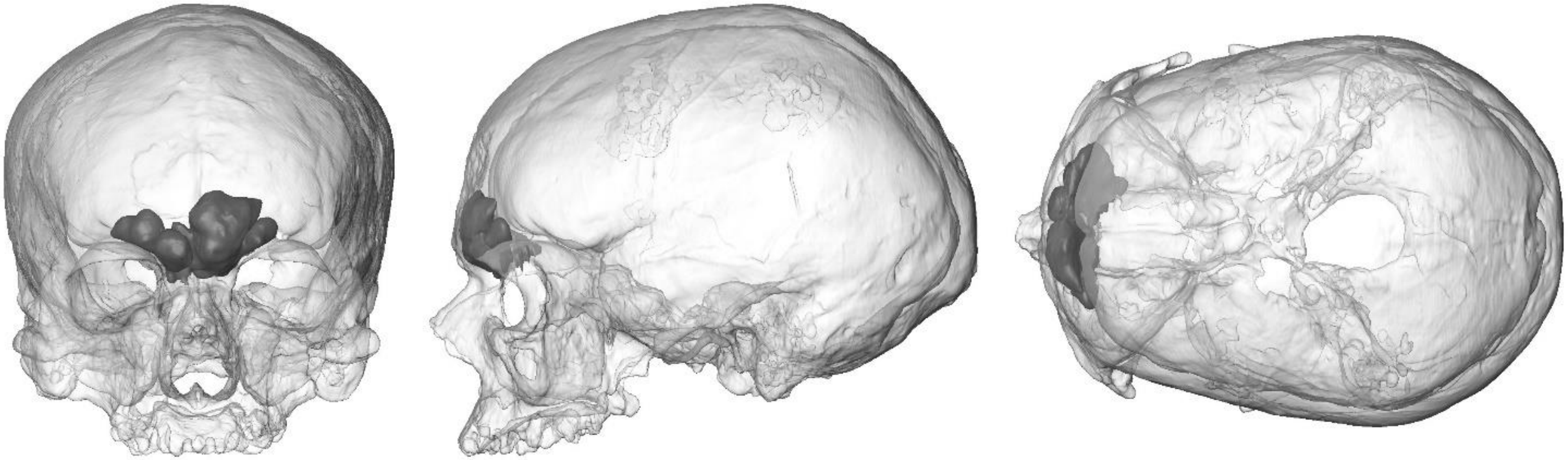

Fig. S67. Cro-Magnon 1, *Homo sapiens*

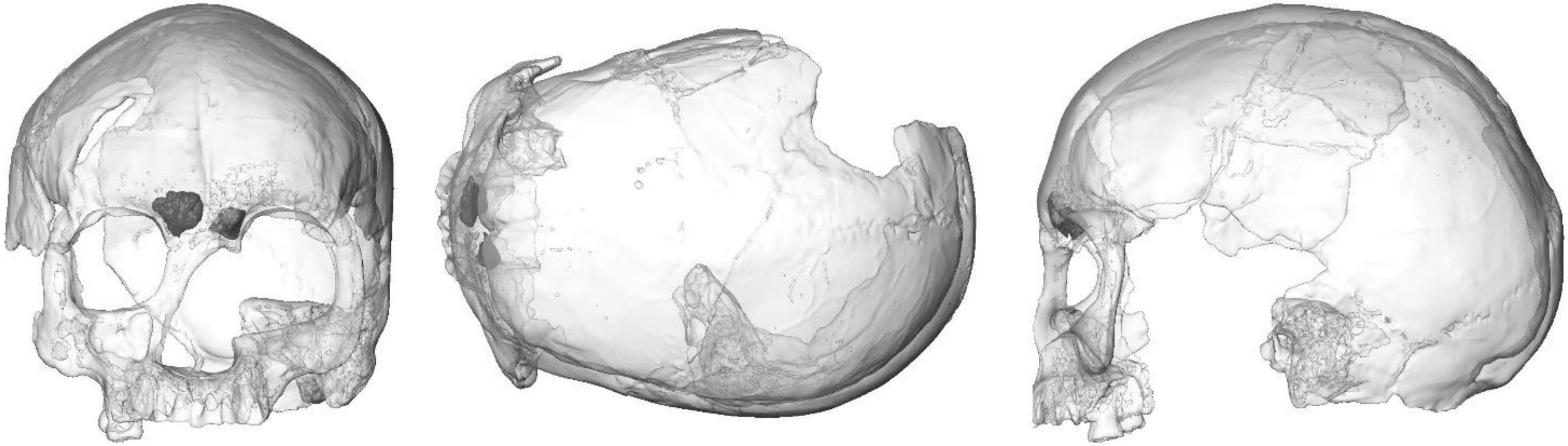

Fig. S68. Cro-Magnon 2, *Homo sapiens*

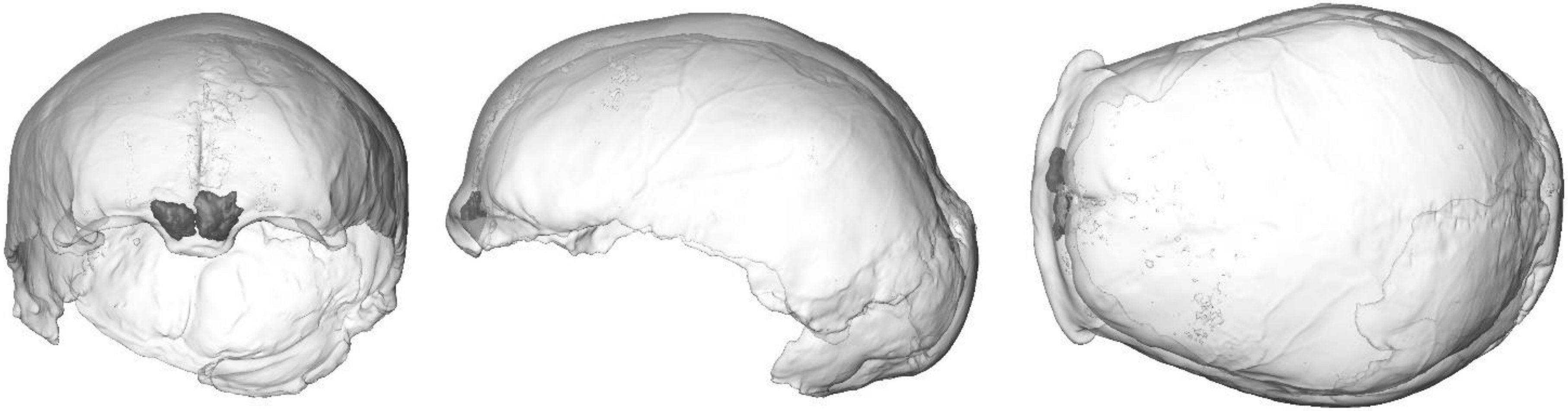

Fig. S69. Cro-Magnon 3, *Homo sapiens*

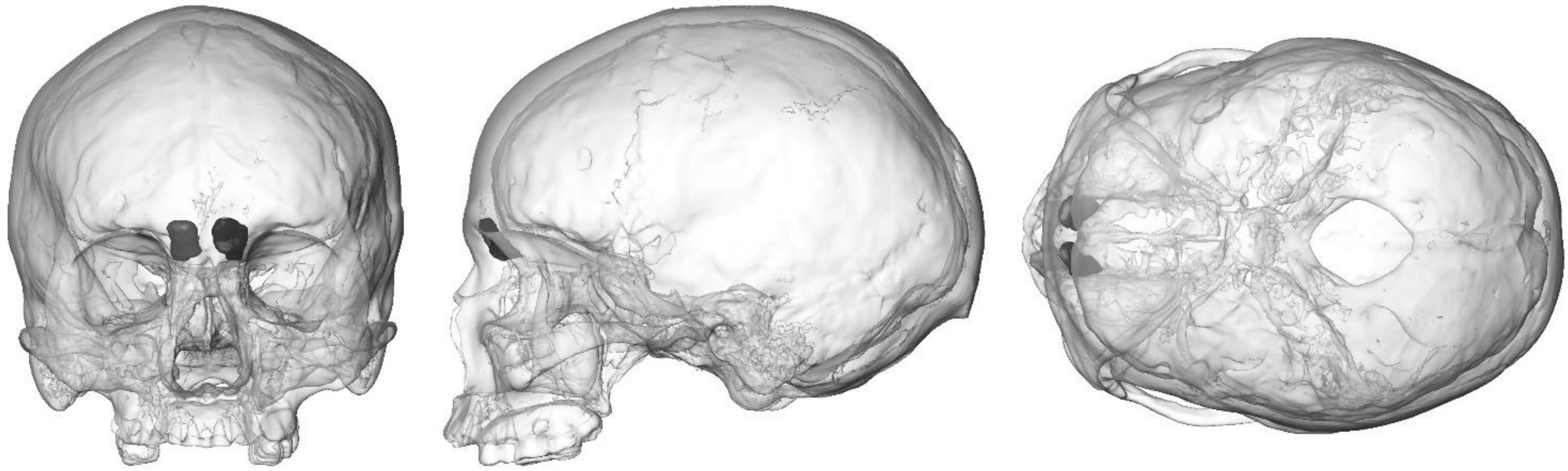

Fig. S70. Pataud 1, *Homo sapiens*

Table S1. Definitions of the linear measurements taken of frontal sinuses (Fig. S1) and of the combined measurements in the perspective of multivariate comparisons (see also 9).

Measurement	Abbreviation	Definition	View
Width	W	Maximum lateral extension of pneumatisation	Anterior
Height	H	Maximum superoinferior height of pneumatisation	Anterior
Anterior length, left	ALl	Maximum infero-medial to supero-lateral length of left sinus	Anterior
Anterior length, right	ALr	Maximum infero-medial to supero-lateral length of right sinus	Anterior
Combined anterior length	2AL	ALr + ALl	
Superior length, left	SLl	Maximum medio-lateral extension of left sinus	Superior
Superior length, right	SLr	Maximum medio-lateral extension of right sinus	Superior
Combined superior length	2SL	SLr + SLl	
Anterior projection	AP	Anteroposterior length	Left lateral
Anterior projection 2	AP2	Most anterior point to most supero-posterior point	Left lateral
Combined anterior dimension	2AP	AP + 2AP	

Table S2. Detailed information for each fossil hominin (individual), including the specific attribution (species), resolution of the imaging dataset (resolution), comment on the preservation of the relevant anatomical area and of the sinuses (preservation), inclusion of the individual in the list of anatomical descriptions (description), inclusion of each individual in the morphometric analyses (morphometric analysis) and corresponding figure in different orientations for each individual (Figure).

Individual	Species	Resolution	Preservation	Description	Morphometric analysis	Figure
TM 266-01-060-1 Toumaï	*Sahelanthropus tchadensis*	0.092 mm^3	Internal structure of bone is altered, bone fragments are mixed with sedimentary filling. However, overall shape and dimensions of pneumatization are preserved.	yes	yes	S2
Taung	*Australopithecus africanus*	0.0555 mm^3	Perfect, but no frontal sinus due to young age.	yes	NA	NA
Sts 5	"	0.5 mm^3	Sinus cavities quite clear, partly filled with sediment. Separation between two sides not preserved. Overall shape and dimensions preserved.	yes	yes	S3
Sts 71	"	0.5 mm^3	Sinusal cavities quite clear, partly filled with sediment, left side of specimen not present, affecting lateral extension of sinuses.<br>Overall shape and dimensions preserved, except for left lateral expansion, but estimation is possible.	yes	yes	S4
StW 505	*A. africanus*	0.391*0.391*1 mm	Good overall preservation. Overall shape and dimensions preserved.	yes	yes	S5
StW 573	*A. prometheus*?	0.08382 mm^3	Area of interest preserved but crushed, with some bone fragments displaced and filled with sediment. Overall shape and dimensions mostly preserved, estimation is possible.	yes	yes	S6

BOU-VP-12/130	*A. garhi*	0.140 mm^3	Medial part of glabellar area and torus well-preserved, but endocranial bone layer incomplete posteriorly and inferiorly. The maximal extension of the sinuses in the different analyzed orientations is preserved and quantifiable. Only the volume is affected by incomplete preservation but can be estimated.	yes	yes	S7
U.W. 88-50	*A. sediba*	0.09142 mm^3	Frontal bone complete but internal structure of bone altered and completely filled with sediment. Overall shape and dimensions mostly preserved.	yes	yes	S8
KNM-WT 17000	*Paranthropus aethiopicus*	0.332*0.332*0.5 mm	Internal structure of bone is altered. Overall shape of right sinus visible. Left sinus apparently limited to an ethmoid cell.	yes	yes	S9
SK 46	*P. robustus*	0.07266 mm^3	Internal structure of bone heavily mineralised and altered. Bone is broken. Only left side of sinuses is preserved.	yes	NA	S10
SK 48	"	0.085 mm^3	Internal structure of bone is heavily mineralised and altered. Overall shape mostly preserved.Some uncertainty in reconstruction of sinuses from available data.	yes	yes	S11
DNH 7	"	surface scan	Global shape of the sinuses preserved but not their posterior extension due to the fragmentation of the fossil between the face and the vault.	yes	NA	S12
DNH 155	"	surface scan	External surface of the frontal torus is mostly missing but allows to estimate the extension of the sinuses in all directions.	yes	yes	S13
KNM-ER 406	*P. boisei*	0.391*0.391*1 mm	CT data do not show information about bone structure. Impossible to evaluate position and extension of sinuses.	NA	NA	NA

OH 5	"	0.488282*0.488282 *1 mm	Overall, good preservation of sinuses. Some infilling of material and sediment, some fragmentation of pneumatic cells, but very minimal compared to many fossils.	yes	yes	S14
StW 53	*Homo gautengensis*?	0.0821 mm^3	Frontal bone broken but sinuses clearly visible. Overall shape and dimensions mostly preserved. Only the volume is affected by incomplete preservation but can be estimated.	yes	yes	S15
SK 847	*H. habilis*	0.3906*0.3906*1 mm	Only left side of skull preserved.	yes	NA	S16
KNM-ER 1805	"	0.254*0.254*0.5 mm	Frontal sinuses do not extend into preserved parts of specimen, namely area of frontal squama just posterior to supraorbital torus.	NA	NA	NA
KNM-ER 1813	"	0.254*0.254*0.5 mm	Bone structure altered, appears homogeneous on CT images. No trace of sinuses, but may be result of taphonomic alteration.	NA	NA	NA
KNM-ER 3833	*H. ergaster / H. erectus*	0.303*0.303*0.5 mm	Overall extension of sinuses is visible but frontal torus is filled with sediment and bone is altered with some fragmentation. 3D reconstruction is not very precise.	yes	yes	S17
KNM-WT 15000	"	0.303*0.303*0.5 mm	Anterior surface of frontal bone absent exposing part of posterior surface of sinuses.	yes	NA	NA
KNM-ER 3733	"	0.303*0.303*0.5 mm	Bone very mineralised, a lot of sedimentary infiltration. Bone structure not well preserved. Frontal sinuses identifiable inferiorly but most of extension not clearly discernible.	NA	NA	NA
OH 9	*H. erectus*	0.5 mm^3	Frontal bone is incomplete, only uppermost extension of sinuses is preserved. Inferior extension and volume estimated.	yes	yes	S18
D2280	*H. erectus / H. georgicus*	0.34375*0.34375*0 .2 mm	Good preservation of internal structure of bone. Sinuses fully preserved.	yes	yes	S19

D2282	"	0.3125*0.3125*0.2 mm	Good preservation of internal structure of bone. Inferior extension of sinuses is lacking but can be estimated.	yes	yes	S20
D2700	"	0.3125*0.3125*0.1 mm	Excellent preservation of internal structure of bone. No sinuses.	yes	yes	S21
D3444	"	0.351*0.351*1 mm	Good preservation of internal structure of bone. Sinuses fully preserved.	yes	yes	S22
D4500	"	0.332*0.332*0.2 mm	Good preservation of internal structure of bone. Sinuses fully preserved.	yes	yes	S23
Trinil 2	*H. erectus*	surface scan	Frontal bone incomplete inferiorly, exposing upper extension of sinuses. Inferior extension of sinuses is lacking but can be estimated. Volume was not estimated because of too incomplete preservation.	yes	yes	S24
Sangiran 17	"	0.5*0.5*0.25 mm	Nearly complete frontal sinus, no alteration of measured dimensions. Shape well preserved.	yes	yes	S25
Skull IX	"	0.34 mm^3	Inferior extension of sinuses not preserved, dimensions including inferior extension slightly underestimated.	yes	yes	S26
Sangiran 27	"	0.49*0.49*1 mm	Left supraorbital torus does not show any cavity, as with glabellar region, but latter is strongly deformed. Presence of sediment and impossibility of differentiating it from fossilized bone elements makes structures of face appear very homogeneous.	NA	NA	NA
Mojokerto	"	0.49*0.49*1 mm	Inferior part of glabellar area is not preserved but frontal sinus visible on left side.	yes	NA	NA
Sambungmacan 1	"	0.49*0.49*1 mm	Bone structure altered, possibly restricted pneumatisation.	yes	NA	NA
Sambungmacan 3	"	0.34 mm^3	Good preservation of bone structure, but inferior extension of the frontal torus not preserved. No sinus.	yes	NA	NA

Sambungmacan 4	"	0.34 mm^3	Very good preservation. Sinuses fully preserved.	yes	yes	S27
Ngandong 1	"	0.49*0.49*1 mm	Well preserved. Inferior extension of the left sinus is exposed. Very small cell on the right side.	yes	yes	S28
Ngandong 2	"	0.49*0.49*1 mm	Frontal bone well preserved, no sinus. Immature individual.	yes	NA	NA
Ngandong 7	"	0.49*0.49*1 mm	Inferior extension exposed but sinuses completely preserved. No impact on measurements.	yes	yes	S29
Ngandong 12	"	0.34 mm^3	Inferior extension exposed but sinuses completely preserved. No impact on measurements.	yes	yes	S30
Ngawi 1	"	0.338*0.338*0.625 mm	Inferior extension exposed but sinuses completely preserved. No impact on measurements. Presence of sediment inside the cavities.	yes	yes	S31
DH 3	*H. naledi*	Surface scan	Only the left part of the frontal bone is preserved.	yes	NA	S32
Lesedi 1	"	0.09652 mm^3	Good preservation of the complete extension of the sinuses.	yes	yes	S33
LB1	*H. floresiensis*	NA	Good preservation of frontal bone but no sinuses present.	yes	NA	NA
Broken Hill NHMUK PA E 686	*H. rhodesiensis*	0.1254 mm^3	Complete and very well preserved.	yes	yes	S34
TD6-15	*H. antecessor*	0.05010006 mm^3	Frontal bone fragmented but global extension of sinuses is visible.	yes	yes	S37
Bilzinglseben 7573	Middle Pleistocene *Homo*	0.03513086 mm^3	Incomplete frontal bone with only postero-medial extension of sinus.	yes	NA	S38
HK75 199	"	0.03513086 mm^3	Medial third of frontal bone only with well preserved sinuses, except for their left lateral extension.	yes	NA	S39
HK 87	"	0.02509851 mm^3	Only most lateral border of sinus is preserved on this small fragment.	yes	yes	S38

Bodo	"	1 mm^3	Nearly complete, extensive and well preserved sinuses.	yes	yes	S35
Ehringsdorf H1024	"	0.081994 mm^3	Medial part of frontal bone with incomplete anterior surface. Overall shape and dimensions mostly preserved. Only the volume is affected by incomplete preservation but can be estimated.	yes	yes	S41
Ehringsdorf H1025	"	0.14806 mm^3	Lateral part of the frontal with no sinus.	yes	NA	NA
Petralona	"	0.53125*0.53125*0.625 mm	Nearly complete. Presence of some sediment inside the cavities and breakage of the fine septa.	yes	yes	S36
Ceprano	"	0.4*1*1 mm	Missing inferior part of sinus and frontal but limited influence on the measurements.	yes	yes	S40
Aroeira	"	0.153*0.153*9.3 mm	Good preservation, complete extension of the sinuses is visible.	yes	yes	S44
SHS 5 (for Sima de los Huesos Skull 5)	"	0.217076*0.217076*0.2 mm	Sinuses fully preserved.	yes	yes	S45
SHS 12	"	0.217076*0.217076*0.2 mm	Nearly complete, except for right medial part of frontal. The volume is affected by incomplete preservation but can be estimated.	yes	yes	S46
SHS 13	"	0.217076*0.217076*0.2 mm	Good preservation.	yes	yes	S47
SHS 15	"	0.217076*0.217076*0.2 mm	Preserves left half of frontal bone and sinuses.	yes	NA	S48
SHS 17	"	0.21965332*0.21965332*0.2 mm	Good preservation, only the inferior extension of the sinuses is exposed. No impact on measurements.	yes	yes	S49

Steinheim	“	0.091407 mm^3	Internal structure affected by post-mortem deformation, extension of sinuses not clear. Some degree of estimation in the measurements but much better appreciation of the global extension compared with previous work.	yes	yes	S42
Zuttiyeh	“	0.09117 mm^3	Very well preserved for the whole extension of the sinuses.	yes	yes	S43
Feldhofer	*H. neanderthalensis*	O.4*1*1 mm	Good preservation, sinuses exposed at their inferior border. No impact on measurements.	yes	yes	S50
La Ferrassie 1	“	0.1316 mm^3	Good preservation, left sinus is exposed endo- and exocranially and its inferior extension is not present. Only the volume is slightly affected by incomplete preservation but can be easily estimated.	yes	yes	S53
La Quina H5	“	0.1092 mm^3	The overall sinus dimensions are preserved but medial third of left frontal sinus is not preserved. Only the volume is affected by incomplete preservation but can be estimated.	yes	yes	S54
Guattari	“	0.4863 mm^3	Sinus are complete and well preserved.	yes	yes	S56
Forbes’ Quarry 1 NHMUK PA EM 3811	“	0.4297 mm^3	Sinus are complete and well preserved.	yes	yes	S55
Krapina 3	“	0.414*0.414*0.5 mm	Sinus are complete and well preserved.	yes	yes	S57
Krapina 6	“	0.44922*0. 44922*0.5 mm	Sinus are complete and well preserved.	yes	yes	S58
La Chapelle aux Saints	“	0.122274 mm^3	Sinus are complete and well preserved.	yes	yes	S59

Spy 1	"	0.444648*0. 444648*0.3 mm	Not completely preserved, sinuses exposed endocranially. Only limited impacts on measurements.	yes	yes	S60
Spy 10	"	0.444648*0. 444648*0.3 mm	Incomplete, medial and inferior extension of sinuses not preserved. The volume had to be be estimated.	yes	yes	S61
Amud	"	0.2 mm^3	Damaged, but extension and shape visible, volume is underestimated.	yes	yes	S51
Apidima 2	"	0.3242*0. 3242*0.4 mm	Incomplete, structure damaged, sinuses crashed, filled with sediment. Global shape preserved.	yes	yes	S52
Tabun C1 NHMUK PA EM 3640	"	0.1271 mm^3	Incomplete, structure damaged by sediment.	yes	yes	S62
LH 18	*H. sapiens?*	0.5156*0.5156*1	Incomplete inferiorly, but rest is well preserved.	yes	yes	S64
Jebel Irhoud 2	*H. sapiens?*	0.0877113 mm^3	Good preservation of the bone structure. No frontal sinus.	yes	NA	NA
Jebel Irhoud 1	*H. sapiens?*	0.091629 mm^3	Quite good preservation, some cracks, some sediment, but overall sinuses are well preserved.	yes	yes	S63
Qafzeh 9	*H. sapiens*	0.2 mm^3	Partial preservation of anteroinferior and posterior extension. Some details of surface are preserved, as is overall extension, some data missing for inferior extension.	yes	yes	S65
Hofmeyr	*H. sapiens*		Very small sinuses, presence of a trace of a metopic suture.	yes	yes	S66
Skhul 5	*H. sapiens*	0.488*0.488*0.5 mm	Sinuses are partly visible but several different filling materials have been used to reconstruct the individual. Not possible to identify extension of the sinuses with confidence.	yes	NA	NA

Cro Magnon 1	*H. sapiens*	0.115 mm^3	Well preserved.	yes	yes	S67
Cro Magnon 2	*H. sapiens*	0.110 mm^3	Well preserved.	yes	yes	S68
Cro Magnon 3	*H. sapiens*	0.1099 mm^3	Well preserved.	yes	yes	S69
Pataud 1	*H. sapiens*	0.1104 mm^3	Well preserved.	yes	yes	S70
Mladeč	*H. sapiens*	0.4668 mm^3	No frontal sinus.	yes	NA	NA
Afalou 2, 30, 34	*H. sapiens*	0.49*0.49*0.63 mm	Well preserved.	yes	yes	Not shown
Afalou 13, 28	*H. sapiens*	0.49*0.49*0.63 mm	No frontal sinus.	yes	NA	Not shown
Taforalt XI C1, XV C4, XV C5	*H. sapiens*	0.49*0.49*0.63 mm	Well preserved.	yes	yes	Not shown
Taforalt XVII C1	*H. sapiens*	0.49*0.49*0.63 mm	No frontal sinus.	yes	NA	Not shown

Table S3. Morphometric data for the pneumatization of the frontal bone for each fossil hominin (when sinuses were measurable). Dimensions are measured as 2D projections in different orientations, the measurements of the extension of the sinuses (as defined in Table S1 and illustrated in Fig. S1) are: maximal lateral extension (W), maximal height (H), maximal length of the left and right sinuses (Anterior Length: ALl and ALr) measured from the most medial and inferior point of the sinus to the more distant point of the extension of the sinus vertically and laterally measured in anterior view; maximal medio-lateral extension of the left and right sinus (Superior Length: SLl and SLr) measured in superior view; length from the most anteriorly protruding point of the sinus to the most posterior point in an horizontal direction (AP) and length from the most anterior point to the maximal supero-posterior extension of the sinuses (AP2) measured in left lateral view as well as the volume. All linear dimensions are in mm, volume is in mm^3. Cells highlighted in yellow indicate individual with a reduced alteration of the extension of the sinuses due to post-mortem alteration, cells highlighted in orange indicate individual with a more important alteration of the sinuses, which necessitated an estimation of partial dimensions (see Table S2 for more information on the preservation of the fossils and altered areas of the sinuses). [a] Sinuses preserved only one side, this specimen is not included in the multivariate analyses. [b] While the maximal extension of the sinuses is preserved, the measurement of sinus volume is affected by incomplete preservation. An estimation was proposed, this parameter being only used as its cube-root as a size-correction factor.

	Anterior view				Superior view		Lateral view		
	W	H	ALl	ALr	SLl	SLr	AP	AP2	Volume
Toumaï	44.9	28.1	23.7	31.5	22.2	30.6	22.2	34.8	18350
Sts 5	45.4	27.4	28.3	30.0	22.3	28.7	24.0	40.7	6100
StW 505	58.4	33.7	31.1	38.8	32.4	40.1	24.1	33.4	9725
Sts 71	29.1	28.1	28.5	26.8	18.5	16.9	11.3	17.5	4530
StW 573	44.5	22.6	21.8	28.8	24.1	26.8	19.8	27.8	6000
U.W. 88-50	41.0	22.2	27.4	23.9	28.2	21.2	12.4	23.1	4790
BOU-VP-12/130	43.3	20.1	29.0	24.3	25.6	19.9	13.0	21.9	4000[b]
KNM-WT 17000	20.6	23.9	0.0	23.9	0.0	21.2	16.9	21.0	3365
SK 48	37.1	24.0	25.5	26.2	25.6	27.0	20.0	24.5	6307
OH 5	63.1	28.7	30.9	28.3	34.3	40.2	28.3	42.8	16600
DNH 155	56.8	32.1	35.1	34.2	38.1	38.6	32.1	32.4	16000
StW 53	46.7	25.6	29.1	28.2	26.7	31.1	22.2	27.6	7500[b]
KNM-ER 3883	80.2	34.2	37.7	33.4	43.7	40.1	22.6	24.6	14000
OH 9	66.4	30.8	33.6	42.4	35.9	40.7	27.1	29.8	12000
D2280	28.1	24.6	29.0	29.5	28.6	27.0	21.5	26.6	8375
D2282	36.4	15.6	18.1	21.9	18.0	21.1	13.3	17.2	3000
D2700	0.0	0.0	0.0	0.0	0.0	0.0	0.0	0.0	0
D3444	33.7	20.3	22.2	19.8	17.0	20.3	18.7	16.2	4100
D4500	50.3	27.6	25.4	36.7	18.0	45.4	22.4	30.6	10450
Trinil 2	55.7	26.0	29.0	32.7	27.5	32.7	24.1	25.3	
Sambungmacan 3	0.0	0.0	0.0	0.0	0.0	0.0	0.0	0.0	0
Sambungmacan 4	58.4	28.8	37.2	27.8	35.6	22.2	21.3	23.1	9600
Sangiran 17	55.7	30.3	35.2	27.6	34.6	26.3	26.6	25.1	13000
Skull IX	65.4	32.5	38.6	28.8	37.3	28.1	23.4	31.2	5000

Ngandong 1	39.6	20.8	22.5	9.0	18.8	8.8	13.5	14.6	2500
Ngandong 7	38.0	19.8	21.4	17.4	19.2	21.0	15.8	13.9	4530
Ngandong 12	42.5	17.6	21.2	20.1	22.9	21.8	19.6	16.5	5200
Ngawi 1	38.8	24.2	27.6	18.4	24.4	17.9	19.5	19.9	6900
LB1	0.0	0.0	0.0	0.0	0.0	0.0	0.0	0.0	0
Lesedi 1	60.6	31.4	37.3	35.8	37.5	36.2	22.2	28.4	12000
La Ferrassie 1	84.4	38.6	38.6	38.1	52.7	36.0	26.1	30.0	21000
La Quina 5	62.7	32.3	31.7	30.4	38.1	29.7	21.3	23.9	10800
Guattari	64.0	29.1	26.5	33.0	28.4	34.3	20.7	25.5	9500
Gilbraltar 1 NHMUK PA EM 3811	53.5	29.8	26.5	34.6	24.2	33.2	19.1	23.1	6900
Krapina 3	57.5	35.6	36.5	32.5	37.4	27.8	14.5	21.3	10200
Krapina 6	60.0	29.1	22.9	34.9	18.6	53.5	22.0	22.3	9370
La Chapelle aux Saints	63.9	28.8	35.7	32.4	31.1	34.1	21.3	24.2	11500
Spy 1	78.4	36.5	39.9	46.1	45.4	42.7	27.5	29.6	18500
Spy 10	65.3	27.2	30.7	33.6	40.0	33.6	22.1	21.8	11000 [b]
Feldhofer	64.9	30.0	31.8	34.8	31.8	35.2	20.8	23.2	12900
Amud	66.8	32.2	31.2	37.0	29.1	35.3	17.1	26.7	9000
Apidima 2	45.1	28.4	32.7	26.6	33.2	21.2	18.3	20.5	7851
Tabun C1 NHMUK PA EM 3640	59.5	31.7	36.4	27.8	27.8	23.8	13.1	21.6	6000
TD6-15	47.0	22.2	25.6	32.5	18.8	29.1	12.0	19.3	5000
Bilzinglseben HK75 199	57.2	31.7	41.4	17.0	43.8	9.7	21.9	23.4	11700
Ehringsdorf H1024	72.5	40.1	36.8	47.8	34.8	47.1	21.7	31.1	18200[b]
Aroeira	63.5	36.5	32.6	42.6	34.8	39.0	25.5	26.2	14485
SHS5	46.9	31.7	30.6	29.8	25.1	24.3	14.1	15.2	7000
SHS12	45.5	30.0	35.7	28.7	33.6	13.7	17.6	21.5	7000[b]
SHS13	39.9	24.2	19.5	27.1	11.7	12.9	11.0	10.2	1880
SHS15[a]	19.7	20.7	20.5	0.0	24.1	0.0	17.9	15.7	2720
SHS17	35.8	16.9	16.2	16.9	12.3	15.6	13.8	8.8	2090
Ceprano	53.3	23.2	29.6	23.7	25.1	19.9	16.2	15.6	6000
Petralona	115.0	60.6	60.6	65.0	74.4	73.1	55.6	58.1	84370
Broken Hill 1 NHMUK PA E 686	91.6	51.3	52.9	42.8	63.4	40.3	31.2	55.9	34900
Bodo	103.2	58.2	60.6	46.0	70.4	43.5	52.3	60.1	64455
Zuttiyeh	53.0	30.8	32.1	28.2	23.4	28.9	18.3	19.8	8665
Steinheim	35.2	19.7	23.9	20.6	23.9	21.1	18.3	22.0	4000
Afalou 2	33.4	27.1	27.8	10.8	23.9	14.4	13.3	16.0	3100
Afalou 12	26.9	23.4	11.7	26.4	10.5	14.5	8.3	7.3	1970
Afalou 30	58.0	37.5	31.3	37.8	25.0	36.5	16.7	14.9	9450

Afalou 34	52.7	39.3	37.0	29.0	30.7	22.0	17.7	23.3	8410
Afalou 13	0.0	0.0	0.0	0.0	0.0	0.0	0.0	0.0	0
Afalou 28	0.0	0.0	0.0	0.0	0.0	0.0	0.0	0.0	0
Taforalt XI C1	31.5	15.2	15.2	12.7	14.1	7.9	7.9	7.9	700
Taforalt XVIIC1	0.0	0.0	0.0	0.0	0.0	0.0	0.0	0.0	0
Taforalt XVC4	30.6	14.1	16.5	8.4	14.0	6.7	5.9	5.2	430
Taforalt XVC5	62.9	28.3	32.5	34.3	29.7	36.0	17.8	23.4	9850
LH 18	49.0	22.8	22.8	24.3	17.5	23.0	17.0	16.0	5000
Jebel Irhoud 1	58.0	27.5	33.4	23.5	47.7	19.4	21.3	22.8	8730
Jebel Irhoud 2	0	0	0	0	0	0	0	0	0
Qafzeh 9	67.9	29.1	31.0	34.6	37.2	40.1	19.1	19.7	7500
Mladec 1	0	0	0	0	0	0	0	0	0
Cro Magnon 2	33.9	17.7	14.8	17.0	9.9	18.8	5.8	6.1	1130
Cro Magnon 3	35.6	19.2	21.8	18.0	18.9	17.4	9.8	10.5	1640
Cro Magnon 1	64.9	35.8	38.9	35.4	41.1	34.7	21.1	18.6	12300
Pataud	33.6	18.1	16.8	17.4	15.2	12.4	7.4	5.7	1230

Table S4. Morphometric data (mm) for the pneumatisation of the frontal bone among extant samples of *Pan*, *Gorilla* and *H. sapiens* and for fossil hominin groups. For definitions of linear dimensions and combined measurements, see Table S1 (results for extant species are from 9).

	W	*H*	*2AL*	*2SL*	*2AP*	*RC V*	*Wr*	*Hr*	*2ALr*	*2SLr*	*2APr*
	Pan paniscus (n = 32)										
Mean	***30.7***	***15.7***	***32.7***	***35.4***	***28.3***	***12.2***	***244.8***	***129.6***	***263.7***	***281.5***	***226.1***
*V**	*41.3*	*33.1*	*39.4*	*41.6*	*40.1*	*28.2*	*21.7*	*23.9*	*21.1*	*20.9*	*22.8*
	Pan troglodytes (n = 33)										
Mean	***40.8***	***18.4***	***43.1***	***51.7***	***41.3***	***16.8***	***241.3***	***110.3***	***258.0***	***303.5***	***243.8***
*V**	*27.4*	*24.3*	*22.7*	*30.2*	*30.1*	*19.8*	*12.8*	*18.1*	*13.5*	*15.2*	*15.3*
	Gorilla (n = 33)										
Mean	***64.9***	***24.1***	***61.1***	***81.5***	***73.2***	***23.7***	***275.8***	***101.7***	***259.2***	***344.2***	***307.7***
*V**	*20.8*	*26.0*	*21.5*	*23.8*	*26.1*	*21.8*	*9.6*	*13.6*	*11.2*	*14.4*	*11.8*
	Homo sapiens (n = 345)										
Mean	***32.9***	***18.4***	***34.1***	***32.3***	***19.9***	***11.6***	***247.3***	***138.1***	***248.4***	***229.2***	***145.7***
*V**	*59.8*	*59.8*	*63.3*	*68.9*	*68.6*	*56.9*	*47.2*	*42.3*	*43.9*	*46.0*	*44.7*
	Australopiths (n = 6)										
Mean	***42.3***	***27.3***	***52.7***	***49.8***	***49.6***	***19.4***	***212.4***	***144.3***	***272.0***	***248.4***	***255.2***
*V**	*40.9*	*15.1*	*30.7*	*45.2*	*31.9*	*22.2*	*24.9*	*17.7*	*26.8*	*30.7*	*23.9*
	Homo erectus (n = 10)										
Mean	***48.5***	***23.9***	***50.0***	***49.9***	***38.8***	**18.1**	**239.0**	**118.4**	**243.8**	**242.4**	**191.3**
*V**	*46.9*	*43.6*	*47.7*	*50.5*	*44.5*	41.1	39.4	38.0	38.9	40.7	37.5
	Homo heidelbergensis (n = 8)										
Mean	***43.9***	***25.6***	***51.1***	***40.5***	***32.9***	***17.1***	***256.7***	***150.7***	***295.3***	***229.2***	***191.6***
*V**	*30.2*	*26.2*	*35.3*	*43.1*	*30.2*	*24.3*	*21.7*	*15.7*	*24.1*	*19.2*	*15.0*
	Homo neanderthalensis (n = 11)										
Mean	***65.6***	***31.7***	***67.2***	***70.2***	***45.8***	***22.6***	***290.3***	***141.2***	***298.2***	***310.6***	***203.3***
*V**	*13.8*	*11.8*	*12.5*	*14.5*	*13.4*	*11.3*	*5.5*	*9.5*	*7.2*	*6.2*	*8.0*
	Homo rhodesiensis (n = 3)										
Mean	***103.0***	***56.5***	***109.8***	***131.1***	***104.0***	***38.9***	***266.1***	***146.3***	***282.8***	***335.9***	***267.8***
*V**	*12.3*	*9.1*	*14.9*	*19.7*	*15.4*	*15.9*	*5.2*	*7.2*	*4.6*	*6.0*	*3.7*

Table S5. Squared Mahalanobis distances obtained after a MANOVA computed on the complete database of absolute dimensions for the frontal sinuses in different geographic samples of *Homo sapiens*.

	Greenland	Pacific	Spain	Alaska	Poland	Continental Asia	Central and South America	West Africa
Greenland		0.67815	3.505	0.22409	1.9146	2.9012	1.6391	3.3646
Pacific	0.67815		1.8101	0.92586	0.68329	2.0272	1.5449	1.835
Spain	3.505	1.8101		3.287	0.5142	1.111	1.4993	0.3152
Alaska	0.22409	0.92586	3.287		1.872	2.7405	1.2323	3.5017
Poland	1.9146	0.68329	0.5142	1.872		1.8273	1.5318	0.89841
Continental Asia	2.9012	2.0272	1.111	2.7405	1.8273		1.2489	0.64335
Central and South America	1.6391	1.5449	1.4993	1.2323	1.5318	1.2489		1.8914
West Africa	3.3646	1.835	0.3152	3.5017	0.89841	0.64335	1.8914	

Table S6. Results from the generalized linear model calculated for PC1 and regions for the different samples of extant *Homo sapiens*.

Effect	Estimate	SE	T	p
Intercept	-1.3835	0.2416	-5.726	2.61e-08 ***
Australia	1.3123	0.4610	2.847	0.004739 **
Busuango Island	2.0547	1.9778	1.039	0.299719
China	2.8024	0.6975	4.018	7.52e-05 ***
Greenland	-0.2575	0.3997	-0.644	0.519951
India	3.1717	0. 6393	4.961	1.21e-06 ***
Java	2.8810	0.7803	3.692	0.000266 ***
Liberia	3.1358	0.6393	4.905	1.57e-06 ***
Mexico	1.6624	0.7803	2.131	0.033981 *
New Britain	3.5160	1.0108	3.478	0.000583 ***
New Zealand	-0.2503	0.5339	-0.469	0.639536
Poland	2.0719	0.3486	5.943	8.15e-09 ***
Spain	3.2046	0.4031	7.949	4.42e-14 ***
Peru	1.7509	1.0108	1.732	0.084320
Philippines	4.0978	1.9778	2.072	0.039169 *
Solomon Islands	-0.4586	1.4089	-0.326	0.745040

Signif. codes: 0 '***' 0.001 '**' 0.01 '*' 0.05 '.' 0.1 ' ' 1, AIC: 1277.8, R^2=0.3386

REFERENCES AND NOTES

1. T. C. Rae, The maxillary sinus in primate paleontology and systematics in *The paranasal sinuses of higher primates*. (Quintessence Books Co., 1999), pp. 177–189.

2. A. A. Farke, Evolution and functional morphology of the frontal sinuses in Bovidae (Mammalia: Artiodactyla), and implications for the evolution of cranial pneumaticity. *Zool. J. Linn. Soc.* **159**, 988–1014 (2010).

3. H. Seidler, D. Falk, C. Stringer, H. Wilfing, G. B. Muller, D. zur Neddenf, G. W. Weberg, W. Reicheish, J.-L. Arsuagai. A comparative study of stereolithographically modelled skulls of Petralona and Broken Hill: Implications for future studies of middle Pleistocene hominid evolution. *J. Hum. Evol.* **33**, 691–703 (1997).

4. H. Prossinger, H. Seidler, L. Wicke, D. Weaver, W. Recheis, C. Stringer, G. Müller, Electronic removal of encrustations inside the Steinheim cranium reveals paranasal sinus features and deformations, and provides a revised endocranial volume estimate. *Anat. Rec.* **273B**, 132–142 (2003).

5. C. Stringer, The status of *Homo heidelbergensis* (Schoetensack 1908). *Evol. Anthropol.* **21**, 101–107 (2012).

6. L. T. Buck, C. B. Stringer, A. M. MacLarnon and T. C. Rae, Variation in paranasal pneumatisation between Mid-Late Pleistocene hominins. *Bull. Mém. Soc. Anthropol. Paris* **31**, 14–33 (2019).

7. T. C. Rae, T. Koppe, Holes in the head: Evolutionary interpretations of the paranasal sinuses in catarrhines. *Evol. Anthropol.* **13**, 211–223 (2004).

8. T. C. Rae, Paranasal pneumatization in extant and fossil Cercopithecoidea. *J. Hum. Evol.* **54**, 279–286 (2008).

9. A. Balzeau, L. Albessard-Ball, A. M. Kubicka, C. Noûs, L. T. Buck, Frontal sinus variation in extant humans and other hominids. *Bulletins et mémoires de la Société d'Anthropologie de Paris* **33**, https://doi.org/10.4000/bmsap.7840 (2021).

10. C. P. E. Zollikofer, M. S. Ponce de León, R. W. Schmitz, C. B. Stringer, New insights into Mid-Late Pleistocene fossil hominin paranasal sinus morphology. *Anat. Rec.* **291**, 1506–1516 (2008).

11. A. Balzeau, L. Buck, L. Albessard, G. Becam, D. Grimmaud-Herve, T. C. Rae, C. B. Stringer, The internal cranial anatomy of the Middle Pleistocene Broken Hill 1 cranium. *PaleoAnthropology* **2017** 107–138 (2017).

12. S. P. Blaney, Why paranasal sinuses? *J. Laryngol. Otol.* **104**, 690–693 (1990).

13. S. Márquez, The paranasal sinuses: The last frontier in craniofacial biology. *Anat. Rec.* **291**, 1350–1361 (2008).

14. J. Keir, Why do we have paranasal sinuses? *J. Laryngol. Otol.* **123**, 4–8 (2009).

15. P. Rhŷs Evans, The paranasal sinuses and other enigmas: An aquatic evolutionary theory. *J. Laryngol. Otol.* **106**, 214–225 (1992).

16. H. P. Howell, XL. Voice production from the standpoint of the laryngologist. *Ann. Otol. Rhinol. Laryngol.* **26**, 643–655 (1917).

17. M. H. Wolpoff, *Paleoanthropology* (McGraw-Hill, 1999).

18. H. Prossinger, F. Bookstein, K. Schafer, H. Seidler, Reemerging stress: Supraorbital torus morphology in the mid-sagittal plane? *Anat. Rec.* **261**, 170–172 (2000).

19. S. J. Gould, R. C. Lewontin, The spandrels of San Marco and the Panglossian paradigm: A critique of the adaptationist programme. *Proc. R. Soc. Lond. B.* **205**, 581–598 (1979).

20. A. M. Tillier, "Les sinus crâniens chez les hommes actuels et fossiles: Essai d'interprétation," thesis, Université de Paris VI, Paris (1975).

21. A. Balzeau, E. Gilissen, Endocranial shape asymmetries in *Pan paniscus*, *Pan troglodytes* and *Gorilla gorilla* assessed via skull based landmark analysis. *J. Hum. Evol.* **59**, 54–69 (2010).

22. D. Curnoe, A review of early *Homo* in southern Africa focusing on cranial, mandibular and dental remains, with the description of a new species (*Homo gautengensis* sp. nov.). *Homo* **61**, 151–177 (2010).

23. J. F. Thackeray, S. R. Loth, M. Laing, E. Swanepoel, M. R. Dayal, K. Lubbe, Comparison of Sts 5 ('Mrs Ples') and Stw 53 ('Early *Homo*') from Sterkfontein, South Africa. *S. Afr. J. Sci.* **96**, 21 (2000).

24. A. R. Hughes, P. V. Tobias. A fossil skull probably of the genus *Homo* from Sterkfontein, Transvaal. *Nature* **265**, 310–312 (1977).

25. F. L'Engle Williams, L. Schroeder, R. R. Ackermann, The mid-face of lower Pleistocene hominins and its bearing on the attribution of SK 847 and StW 53. *Homo* **63**, 245–257 (2012).

26. L. R. Berger, J. Hawks, D. J. de Ruiter, S. E. Churchill, P. Schmid, L. K. Delezene, T. L. Kivell, H. M. Garvin, S. A. Williams, J. M. De Silva, M. M. Skinner, C. M. Musiba, N. Cameron, T. W. Holliday, W. Harcourt-Smith, R. R. Ackermann, M. Bastir, B. Bogin, D. Bolter, J. Brophy, Z. D. Cofran, K. A. Congdon, A. S. Deane, M. Dembo, M. Drapeau, M. C. Elliott, E. M. Feuerriegel, D. Garcia-Martinez, D. J. Green, A. Gurtov, J. D. Irish, A. Kruger, M. F. Laird, D. Marchi, M. R. Meyer, S. Nalla, E. W. Negash, C. M. Orr, D. Radovcic, L. Schroeder, J. E. Scott, Z. Throckmorton, M. W. Tocheri, C. Van Sickle, C. S. Walker, P. Wei, B. Zipfel, *Homo naledi*, a new species of the genus *Homo* from the Dinaledi Chamber, South Africa. *eLife* **4**, e09560 (2015).

27. G. P. Rightmire, A. Margvelashvili, D. Lordkipanidze, Variation among the Dmanisi hominins: Multiple taxa or one species? *Am. J. Phys. Anthropol.* **168**, 481–495 (2019).

28. G. P. Rightmire, *Homo* in the Middle Pleistocene: Hypodigms, variation, and species recognition. *Evol. Anthropol.* **17**, 8–21 (2008).

29. S. C. Antón, H. H. G. Taboada, E. R. Middleton, C. W. Rainwater, A. B. Taylor, T. R. Turner, J. E. Turnquist, K. J. Weinstein, S. A. Williams, Morphological variation in *Homo erectus* and the origins of developmental plasticity. *Phil. Trans. R. Soc. B* **371**, 20150236 (2016).

30. I. Tattersall, Neanderthals, *Homo sapiens*, and the question of species in paleoanthropology. *J. Anthropol. Sci.* **85**, 139–146 (2007).

31. J. H. Schwartz, I. Tattersall, Z. Chi, Comment on "a complete skull from Dmanisi, Georgia, and the evolutionary biology of EarlyHomo". *Science* **344**, 360 (2014).

32. K. L. Baab, The taxonomic implications of cranial shape variation in *Homo erectus*. *J. Hum. Evol.* **54**, 827–847 (2008).

33. V. Zeitoun, The taxinomical position of the skull of Zuttiyeh. *C. R. Acad. Sci. Ser. IIA Earth Planet. Sci.* **332**, 521–525 (2001).

34. S. E. Freidline, P. Gunz, I. Janković, K. Harvati, J. J. Hublin, A comprehensive morphometric analysis of the frontal and zygomatic bone of the Zuttiyeh fossil from Israel. *J. Hum. Evol.* **62(2)**, 225–41 (2012).

35. T. C. Rae, T. Koppe, C. B. Stringer, The Neanderthal face is not cold adapted. *J. Hum. Evol.* **60**, 234–239 (2011).

36. J. L. Arsuaga, I. Martínez, L. J. Arnold, A. Aranburu, A. Gracia-Téllez, W. D. Sharp, R. M. Quam, C. Falguères, A. Pantoja-Pérez, J. Bischoff, E. Poza-Rey, J. M. Parés, J. M. Carretero, M. Demuro, C. Lorenzo, N. Sala, M. Martinón-Torres, N. García, A. Alcázar de Velasco, G. Cuenca-Bescós, A. Gómez-Olivencia, D. Moreno, A. Pablos, C.-C. Shen, L. Rodríguez, A. I. Ortega, R. García, A. Bonmatí, J. M. Bermúdez de Castro, E. Carbonell, Neandertal roots: Cranial and chronological evidence from Sima de los Huesos. *Science* **344**, 1358–1363 (2014)

37. L. T. Buck, C. B. Stringer, *Homo heidelbergensis*. *Curr. Biol.* **24**, R214–R215 (2014).

38. M. Kuhlwilm, I. Gronau, M. J. Hubisz, C. de Filippo, J. Prado-Martinez, M. Kircher, Q. Fu, H. A. Burbano, C. Lalueza-Fox, M. de la Rasilla, A. Rosas, P. Rudan, D. Brajkovic, Ž. Kucan, I. Gušic, T. Marques-Bonet, A. M. Andrés, B. Viola, S. Pääbo, M. Meyer, A. Siepel, S. Castellano, Ancient gene flow from early modern humans into Eastern Neanderthals. *Nature* **530**, 429–33 (2016).

39. A. Balzeau, J. Radovcic, Variation and modalities of growth and development of the temporal bone pneumatization in Neandertals. *J. Hum. Evol.* **54**, 546–567 (2008).

40. K. Prüfer, C. de Filippo, S. Grote, F. Mafessoni, P. Korlević, M. Hajdinjak, B. Vernot, L. Skov, P. Hsieh, S. Peyrégne, D. Reher, C. Hopfe, S. Nagel, T. Maricic, Q. Fu, C. Theunert, R. Rogers, P.

Skoglund, M. Chintalapati, M. Dannemann, B. J. Nelson, F. M. Key, P. Rudan, Željko Kućan, I. Gušić, L. V. Golovanova, V. B. Doronichev, N. Patterson, D. Reich, E. E. Eichler, M. Slatkin, M. H. Schierup, A. M. Andrés, J. Kelso, M. Meyer, S. Pääbo, A high-coverage Neandertal genome from Vindija cave in Croatia. *Science* **358**, 655–658 (2017).

41. A. Balzeau, J. Badawi-Fayad, La morphologie externe et interne de la région supra-orbitaire est-elle corrélée à des contraintes biomécaniques ? Analyses structurales des populations d'*Homo sapiens* d'Afalou Bou Rhummel (Algérie) et de Taforalt (Maroc). *BMSAP* **17**, 185–197 (2005).

42. R. M. Godinho, P. O'Higgins, The biomechanical significance of the frontal sinus in Kabwe 1 (*Homo heidelbergensis*). *J. Hum. Evol.* **114**, 141–153 (2018).

43. M. L. Noback, E. Samo, C. H. A van Leeuwen, N. Lynnerup, K. Harvati, Paranasal sinuses: A problematic proxy for climate adaptation in Neanderthals. *J. Hum. Evol.* **97**, 176–179 (2016).

44. C. S. Coon, *The Origin of Races* (Alfred A. Knopf, 1962).

45. S. E. Churchill, Cold adaptation, heterochrony and Neandertals. *Evol. Anthropol.* **7**, 46–60 (1998).

46. A. Balzeau, R. L. Holloway, D. Grimaud-Hervé, Variations and asymmetries in regional brain surface in the genus *Homo*. *J. Hum. Evol.* **62**, 696–706 (2012).

47. G. W. Weber, I. Hershkovitz, P. Gunz, S. Neubauer, A. Ayalon, B. Latimer, M. Bar-Matthews, G. Yasur, O. Barzilai, H. May, Before the massive modern human dispersal into Eurasia: A 55,000-year-old partial cranium from Manot Cave, Israel. *Quat.* **551**, 29–39 (2020).

48. A. W. Toga, K. L. Narr, P. M. Thompson, E. Luders, Brain Asymmetry: Evolution, in *Encyclopedia of Neuroscience* (Academic Press, 2009), pp. 303–311.

49. E. Zaidel, Brain asymmetry, in *International Encyclopedia of the Social & Behavioral Sciences* (Elsevier, 2001), pp. 1321–1329.

50. S. D. Christman, A history of brain asymmetry studies, in *Reference Module in Neuroscience and Biobehavioral Psychology* (Elsevier, 2018).

51. L. E. Copes, “Comparative and Experimental Investigations of Cranial Robusticity in Mid-Pleistocene Hominins,” thesis, Arizona State University (2012).

52. A. Balzeau, E. Gilissen, W. Wendelen, W. Coudyzer, Internal cranial anatomy of the type specimen of *Pan paniscus* and available data for study. *J. Hum. Evol.* **56**, 205–208 (2009).

53. A. Balzeau, P. Charlier, What do cranial bones of LB1 tell us about *Homo floresiensis*? *J. Hum. Evol.* **93**, 12–24 (2016).

54. O. Hammer, D. A. T. Harper, P. D. Ryan, PAST: Paleontological statistics software package for education and data analysis. *Palaeontol. Electron.* **4**, 9 (2001).

55. V. Amrhein, S. Greenland, B. McShane, Scientists rise up against statistical significance. *Nature* **567**, 305–307 (2019).

56. R. R. Sokal, C. A. Braumann, Significance tests for coefficients of variation and variability profiles. *Syst. Biol.* **29**, 50–66 (1980).

57. B. Wood, D. E. Lieberman, Craniodental variation in *Paranthropus boisei*: A developmental and functional perspective. *Am. J. Phys. Anthropol.* **116**, 13–25 (2001).

58. R. L. Miller, J. S. Kahn, *Statistical Analysis in the Geological Sciences* (Wiley, 1962)

59. F. E. Grine, H. F. Smith, C. P. Heesy, E. J. Smith, Phenetic affinities of Plio-Pleistocene *Homo* fossils from South Africa: Molar cusp proportions, in *The First Humans: Origin and Early Evolution of the Genus Homo* (Springer, 2009), pp. 49–62.

60. P. V. Tobias, *Olduuai Gorge*, *The Cranium and Maxillary Dentition of Australopithecus (ZinJanthropus) boisei* (Cambridge Univ. Press, 1967), vol. 2, pp. 264.

61. B. Asfaw, The Belohdelie frontal: New evidence of early hominid cranial morphology from the Afar of Ethiopia. *J. Hum. Evol.* **16**, 611–624 (1987).

62. F. Weidenreich, *The Skull of Sinanthropus Pekinensis; a Comparative Study on a New Primitive Hominid Skull* (Geological Survey of China, 1943).

63. F. Weidenreich, G. H. R. von Koenigswald, *Morphology of Solo Man. Anthropological papers of the AMNH* (The American Museum of Natural History, 1951), vol. 43, pp. 205–290.

64. A. M. Tillier, La pneumatisation du massif cranio-facial chez les hommes actuels et fossiles (suite). *Bull. Mém. Soc. Anthropol. Paris* **4**, 287–316 (1977).

65. X. Wu, F. E. Poirier, *Human Evolution in China: A Metric Description of the Fossils and a Review of the Sites* (Oxford Univ. Press, 1995).

66. A. Balzeau, "Spécificités des caractères morphologiques internes du squelette céphalique chez *Homo erectus*," thesis, Département de Préhistoire, Muséum national d'Histoire naturelle, Paris (2005).

67. W. H. Gilbert, R. L. Holloway, D. Kubo, R. T. Kono, G. Suwa, *Tomographic Analysis of the Daka calvaria. Homo erectus: Pleistocene Evidence From the Middle Awash, Ethiopia.* (University of California Press, 2008), pp. 329–347.

68. A. Vialet, G. Guipert, H. Jianing, F. Xiaobo, L. Zune, W. Youping, L. Tianyuan, M.-A. de Lumley, H. de Lumley, *Homo erectus* from the Yunxian and Nankin Chinese sites: Anthropological insights using 3D virtual imaging techniques. *C. R. Palevol* **9**, 331–339 (2010).

69. G. Busk, Observations on a Systematic Mode of Craniometry, in *Transactions of the Ethnological Society of London* (Royal Anthropological Institute of Great Britain and Ireland, 1861), vol. 1, pp. 341–348.

70. C. C. Blake, On the alleged peculiar characters, and assumed antiquity of the human cranium from the Neanderthal, in *Journal of the Anthropological Society of London* (1864), vol. 2, pp. cxxxix–clvii.

71. D. S. Brose, M. H. Wolpoff, Early Upper Paleolithic man and Late Middle Paleolithic tools. *Am. Anthropol.* **73**, 1156–1194 (1971).

72. T. C. Rae, T. Koppe, Isometric scaling of maxillary sinus volume in hominoids. *J. Hum. Evol.* **38**, 411–423 (2000).

73. D. L. Daniels, M. F. Mafee, M. M. Smith, T. L. Smith, T. P. Naidich, W. D. Brown, W. E. Bolger, L. P. Mark, J. L. Ulmer, L. Hacein-Bey, J. M. Strottmann, The frontal sinus drainage pathway and related structures. *Am. J. Neuroradiol.* **24**, 1618–1627 (2003).

74. R. L. Holloway, M. C. de la Costelareymondie, Brain endocast asymmetry in pongids and hominids: Some preliminary findings on the paleontology of cerebral dominance. *Am. J. Phys. Anthropol.* **58**, 101–110 (1982).

75. N. Mantel, The detection of disease clustering and a generalized regression approach. *Cancer Res.* **27**, 209–220 (1967).

76. M. G. Leakey, F. Spoor, F. H. Brown, P. N. Gathogo, C. Kiarie, L. N. Leakey, I. McDougall, New hominin genus from eastern Africa shows diverse middle Pliocene lineages. *Nature* **410**, 433–440 (2001).

77. R. S. Lacruz, F. R. Rozzi, T. G. Bromage, Dental enamel hypoplasia, age at death, and weaning in the Taung child: Research letter. *S. Afr. J. Sci.* **101**, 567–569 (2005).

78. K. Moore, A. Ross, Frontal sinus development and juvenile age estimation. *Anat. Rec.* **300**, 1609–1617 (2017).

79. L. R. Berger, D. J. De Ruiter, S. E. Churchill, P. Schmid, K. J. Carlson, P. H. G. M. Dirks, J. M. Kibii, *Australopithecus sediba*: A new species of *Homo*-like australopith from south Africa. *Science* **328**, 195–204 (2010).

80. S. C. Antón, Developmental age and taxonomic affinity of the Mojokerto child, Java, Indonesia. *Am. J. Phys. Anthropol.* **102**, 497–514 (1997).

81. N. B. Gadekar, V. S. Kotrashetti, J. Hosmani, R. Nayak, Forensic application of frontal sinus measurement among the Indian population. *J. Oral Maxillofac. Pathol.* **23**, 147–151 (2019).

82. M. Čechová, J. Dupej, Jaroslav Brůžek, Š. Bejdová, M. Horák, J. Velemínská, Sex estimation using external morphology of the frontal bone and frontal sinuses in a contemporary Czech population. *Int. J. Leg. Med.* **133**, 1285–1294 (2019).

83. A. P. Kumar, N. Doggalli, K. Patil, Frontal sinus as a tool in identification. *J. Forensic Odontol.* **3**, 55–58 (2018).

84. S. S. Nikam, R. M. Gadgil, A. R. Bhoosreddy, K. R. Shah, V. U. Shirsekar, Personal identification in forensic science using uniqueness of radiographic image of frontal sinus. *J. Forensic Odontol.* **33**, 1–7 (2015).

85. J. Cvrček, P. Veleminsky, Contribution to the study of frontal sinus familial similarity based on genealogically documented individuals (Bohemia, 19th to 20th centuries). *J. Nat. Mus.* **189**, 21–30 (2020).

86. G. Suwa, B. Asfaw, R. T. Kono, D. Kubo, C. O. Lovejoy, T. D. White, The *Ardipithecus ramidus* skull and its implications for hominid origins. *Science* **326**, 68–68e7 (2009).

87. Y. Haile-Selassie, S. M. Melillo, A. Vazzana, S. Benazzi, T. M. Ryan, A 3.8-million-year-old hominin cranium from Woranso-Mille, Ethiopia. *Nature* **573**, 214–219 (2019).

88. W. H. Kimbel, Y. Rak, D. C. Johanson, The Skull of *Australopithecus Afarensis*, in *Human Evolution Series* (Oxford Univ. Press, 2004).

89. B. Brown, A. Walker, C. V. Ward, R. E. Leakey, New *Australopithecus boisei* calvaria from East Lake Turkana, Kenya. *Am. J. Phys. Anthropol.* **91**, 137–159 (1993).

90. R. E. F. Leakey, A. C. Walker, New australopithecines from east Rudolf, Kenya (III). *Am. J. Phys. Anthropol.* **39**, 205–221 (1973).

91. B. Wood, *Koobi Fora Research Project*, *Volume 4*, *Hominid Cranial Remains* (Clarendon Press, 1991).

92. R. E. F. Leakey, A. Walker, New *Australopithecus boisei* specimens from East and West lake Turkana, Kenya. *Am. J. Phys. Anthropol.* **76**, 1–24 (1988).

93. P. V. Tobias, *Olduvai Gorge 2 Part Set: Volume 4, The Skulls, Endocasts and Teeth of Homo habilis* (Cambridge Univ. Press, 1991).

94. P. Brown, LB1 and LB6 *Homo floresiensis* are not modern human (*Homo sapiens*) cretins. *J. Hum. Evol.* **62**, 201–224 (2012).

95. D. Falk, C. Hildebolt, K. Smith, M. J. Morwood, T. Sutikna, Jatmiko, E. Wayhu Saptomo, H. Imhof, H. Seidler, F. Prior, Brain shape in human microcephalics and *Homo floresiensis*. *Proc. Natl. Acad. Sci.* **104**, 2513–2518 (2007).

96. C. Buzi, A. Profico, F. Di Vincenzo, K. Harvati, M. Melchionna, P. Raia, G. Manzi, Retrodeformation of the Steinheim cranium: Insights into the evolution of Neanderthals. *Symmetry* **13**, 1611 (2021).

97. E. Stansfield, P. Mitteroecker, S. Y. Vasilyev, L. N. Butaric, Respiratory adaptation to climate in modern humans and Upper Palaeolithic individuals from Sungir and Mladeč. *Sci. Rep.* **11**, 7997 (2021).

98. A. Lipphaus, U. Witzel, Three-dimensional finite element analysis of the dural folds and the human skull under head acceleration. *Anat. Rec.*, **304**(2), 384–392 (2021).

99. D. Falk, C. P. E. Zollikofer, N. Morimoto, M. S. Ponce de Leon, Metopic suture of Taung (*Australopithecus africanus*) and its implications for hominin brain evolution. *Proc. Natl. Acad. Sci.* **109**, 8467–8470 (2012).

100. A. Guerram, J. M. Le Minor, S. Renger, G. Bierry, Brief communication: The size of the human frontal sinuses in adults presenting complete persistence of the metopic suture. *Am. J. Phys. Anthropol.* **154**(4), 621–627 (2014).

101. A. Balzeau, I. Crevecoeur, H. Rougier, A. Froment, E. Gilissen, D. Grimaud-Hervé, P. Mercier, P. Semal, Applications of imaging methodologies to paleoanthropology: Beneficial results relating to the preservation, management and development of collections. *C. R. Palevol* **9**, 265–275 (2010).

102. M. S. Ponce de León, T. Koesbardiati, J. D. Weissmann, M. Milella, C. S. Reyna-Blanco, G. Suwa, O. Kondo, A. S. Malaspinas, T. D. White, C. P. E. Zollikofer, Human bony labyrinth is an indicator of population history and dispersal from Africa. *Proc. Natl. Acad. Sci.* **115**, 4128–4133 (2018).

103. T. Koertvelyessy, Relationships between the frontal sinus and climatic conditions: A skeletal approach to cold adaptation. *Am. J. Phys. Anthropol.* **37**, 161–172 (1972).

104. C. L. Hanson, D. W. Owsley, Frontal sinus size in Eskimo populations. *Am. J. Phys. Anthropol.* **53**, 251–255 (1980).

105. A. Beaudet, E. Bruner, A frontal lobe surface analysis in three archaic African human fossils: OH 9, Buia, and Bodo. *C. R. Palevol* **16**, 499–507 (2017).

106. A. S. Pereira-Pedro, J. K. Rilling, X. Chen, T. M. Preuss, E. Bruner, Midsagittal brain variation among non-human primates: Insights into evolutionary expansion of the human precuneus. *Brain Behav. Evol.* **90**, 255–263 (2017).
